# Cognitive Remediation Works But How Should We Provide It? An Adaptive Randomized Controlled Trial of Delivery Methods Using a Patient Nominated Recovery Outcome in First-Episode Participants

**DOI:** 10.1093/schbul/sbac214

**Published:** 2023-03-03

**Authors:** Til Wykes, Dominic Stringer, Janette Boadu, Rose Tinch-Taylor, Emese Csipke, Matteo Cella, Andrew Pickles, Paul McCrone, Clare Reeder, Max Birchwood, David Fowler, Kathryn Greenwood, Sonia Johnson, Jesus Perez, Rosa Ritunnano, Andrew Thompson, Rachel Upthegrove, Jon Wilson, Alex Kenny, Iris Isok, Eileen M Joyce

**Affiliations:** Institute of Psychiatry, Psychology and Neuroscience, King’s College London, London, UK; South London and Maudsley NHS Foundation Trust, London, UK; Institute of Psychiatry, Psychology and Neuroscience, King’s College London, London, UK; Institute of Psychiatry, Psychology and Neuroscience, King’s College London, London, UK; Institute of Psychiatry, Psychology and Neuroscience, King’s College London, London, UK; Institute of Psychiatry, Psychology and Neuroscience, King’s College London, London, UK; Institute of Psychiatry, Psychology and Neuroscience, King’s College London, London, UK; South London and Maudsley NHS Foundation Trust, London, UK; Institute of Psychiatry, Psychology and Neuroscience, King’s College London, London, UK; School of Health Sciences, University of Greenwich, London, UK; Institute of Psychiatry, Psychology and Neuroscience, King’s College London, London, UK; Warwick Medical School, University of Warwick, Coventry, UK; School of Psychology, University of Sussex, Brighton, UK; School of Psychology, University of Sussex, Brighton, UK; Faculty of Brain Sciences, University College London, London, UK; Cambridgeshire and Peterborough NHS Foundation Trust, Cambridge, UK; Warwick Medical School, University of Warwick, Coventry, UK; Warwick Medical School, University of Warwick, Coventry, UK; School of Psychology, University of Birmingham, Birmingham, UK; Norfolk and Suffolk NHS Foundation Trust, Norwich, UK; Patient Advisory Board, King’s College London, London, UK; Patient Advisory Board, King’s College London, London, UK; UCL Queen Square Institute of Neurology, University College London, London, UK

**Keywords:** therapist support, early intervention, goal achievement, functioning, cognitive training

## Abstract

**Background and Hypothesis:**

Cognitive remediation (CR) benefits cognition and functioning in psychosis but we do not know the optimal level of therapist contact, so we evaluated the potential benefits of different CR modes.

**Study Design:**

A multi-arm, multi-center, single-blinded, adaptive trial of therapist-supported CR. Participants from 11 NHS early intervention psychosis services were independently randomized to Independent, Group, One-to-One, or Treatment-as-usual (TAU). The primary outcome was functional recovery (Goal Attainment Scale [GAS]) at 15-weeks post randomization. Independent and TAU arms were closed after an interim analysis, and three informative contrasts tested (Group vs One-to-One, Independent vs TAU, Group + One-to-One vs TAU). Health economic analyses considered the cost per Quality Adjusted Life Year (QALY). All analyses used intention-to-treat principles.

**Study Results:**

We analyzed 377 participants (65 Independent, 134 Group, 112 One-to-One, 66 TAU). GAS did not differ for Group vs One-to-One: Cohen’s *d*: 0.07, −0.25 to 0.40 95% CI, *P* = .655; Independent vs TAU: Cohen’s *d*: 0.07, −0.41 to 0.55 95% CI, *P* = .777. GAS and the cognitive score improved for Group + One-to-One vs TAU favoring CR (GAS: Cohen’s *d*: 0.57, 0.19–0.96 95% CI, *P* = .003; Cognitive score: Cohens *d*: 0.28, 0.07–0.48 95% CI, *P* = .008). The QALY costs were £4306 for Group vs TAU and £3170 for One-to-One vs TAU. Adverse events did not differ between treatment methods and no serious adverse events were related to treatment.

**Conclusions:**

Both active therapist methods provided cost-effective treatment benefiting functional recovery in early psychosis and should be adopted within services. Some individuals benefited more than others so needs further investigation.

**Trial registration:**

ISRCTN14678860 https://doi.org/10.1186/ISRCTN14678860*Now closed*.

## Introduction

Cognitive function is the strongest predictor of social and occupational functioning 4 years later^1–3^ and limits opportunities offered by evidence-based rehabilitation.^4^ Cognitive remediation (CR) was developed using the simple model that boosting cognition benefits functioning. Although studies show only partial mediation, meta-analyses have shown durable benefits of CR^5–9^ and some national guidelines now recommend it.^10–13^ The CR White Paper^14^ highlighted four effective elements: cognitive exercise, developing problem-solving strategies, an active therapist, and facilitating transfer to real-world functioning. A recent meta-analysis demonstrated that CRs with all these elements improved cognitive and functioning benefits.^9^ One programme has all elements, Cognitive Interactive Remediation of Cognition and Thinking Skills or “CIRCuiTS”, and uniquely facilitates the link between cognitive functioning and everyday life by incorporating metacognitive training into the software and therapy interactions.^15–17^

Service users have positive views about CR therapists,^18,19^ and they facilitate therapeutic benefit,^8,9^ but the White Paper does not define an “active therapist”. Therapist access provided either in a group or one-to-one is usual, but a CR embedding the three other key elements (cognitive exercise, strategy training, and transfer to real-life functioning) might reduce the importance of a therapist.

This study was designed to identify how much therapist time would provide an efficient and cost-effective CR service within UK NHS Early Intervention Services (EIS) to inform implementation. We tested 3 widely used therapy modalities with different therapist involvement.^16,20–23^ EIS was chosen as an early benefit that may alleviate future problems and improve life opportunities. Although we know CR is effective in EIS,^24–27^ the chosen methods have different costs, and the balance between costs and outcomes is important for large-scale roll-out. EIS has comprehensive case management that includes regular contact with a care coordinator, medication management, psychiatric consultation, crisis management, physical health assessment, and psychological therapies so it offers a stringent test of the extra CR benefit. CR trials assess functional outcomes using self-report, clinician observation, or tests of functional capacity.^28^ We consulted clinical staff and service users about the outcomes that would persuade them that CR was worth investing in and they said it was whether CR helped patients to attain their personal goals. We therefore, chose a valid and psychometrically sound functional outcome scale that is sensitive to change in clinical trials and has greater face validity than global measures (Goal Attainment Scale [GAS]^29,30^). This choice has the benefit of capturing the heterogeneous personal goals and aspirations of EIS patients with some wishing to return to education, others aiming to start employment or wanting more social activities.

## Methods

### Study Design

A 4-arm multi-center, single-blinded, adaptive, randomized controlled trial comparing 3 CR implementation methods compared to treatment-as-usual (TAU) in people presenting with non-affective psychosis in UK NHS EIS. Outcomes were measured at weeks 0, 15, and 39. Treatment was provided independently (at home with phone contact and drop-in clinics), in groups or one-to-one within a 12-week time window. Camden and Kings Cross NHS Research Ethics Committee (ref. number 15/LO/1960) provided a favorable review. An Independent Data Monitoring Committee (IDMC) oversaw study progression, adverse events, and statistical analyses.

### Study Sample

The inclusion criteria were: EIS care for at least 3 months, clinical stability judged by the clinical team, 16–45 years, a research diagnosis of non-affective psychosis assessed by the MINI.^31^ Exclusion criteria were communication difficulties in completing assessments, an organic condition affecting cognition, a learning disability or a definitive bipolar disorder diagnosis. Six sites (North London; South London; Cambridge; Warwick; Sussex; and Birmingham—see Supplementary) ensured a wide-ranging community backdrop of urbanicity and ethnicity.

### Randomization and Masking

Consented participants were initially randomized in blocks of 15, stratified by the site in proportions 4:4:3:4 (Group: Independent: One-to-One: TAU) using a concealed sequence on an independent web-based King’s Clinical Trials Unit system following baseline assessment. We changed following slow recruitment to allow 11–15 participant blocks and later individual randomization with equal allocation, first to 4 and then 2 arms (Group, One-to-One) following an interim analysis. The outcome assessors, trial manager, and investigators were blind to the trial arm, including the senior trial statistician until primary analysis completion.

### Intervention

The therapist-supported CR computerized CR CIRCuiTS programme was used. It was co-developed with service users and therapists^15,32^ and is based on cognitive practice, strategy use and metacognition engagement, a pedagogical factor that allows skill transfer to other situations. Cognitive tasks and exercises (modeling community skills such as traveling or texting) are graduated with movement to higher levels depending on performance. Therapists encourage participants to regulate and monitor their cognitive performance through improved metacognitive awareness using strategies and cognitive skills learned through the programme^16^ (see Supplementary p5s–6s). Therapy plans are based on the participant’s goals to facilitate therapeutic engagement. Treatment arms differed in therapist contact hours (see Supplementary p6s for detail). The arms were:

- ***One-to-One*** (a single participant) receives 10.5 weeks of twice weekly therapy, up to 42 h in total, with sessions lasting 60 to 180 min, split into 3 parts: (1) 20–60 min of CR with a therapist; (2) 20–60 min of in vivo transfer work (ie, putting CR strategies into real life); (3) 20–60 min of independent CR, with (2) and (3) depending on the stage of independence
- ***Group*** (max 4 participants) receives 14 weeks of 3 times weekly CR with a single shared therapist. Sessions last up to 90 min and begin and end with group activities related to goal setting and metacognition.
- ***Independent*** participants receive one therapist session for orientation and up to 41 independent sessions. Therapists offer telephone contact or drop-in sessions on an as-needed basis not exceeding 1 h contact time per fortnight.

The therapy window was constrained to 12 weeks and missing sessions were not replaced. Therapists were trained graduate-level psychologists (25–30 h training for up to 12 weeks). They delivered all 3 treatment arms and were supervised weekly by an experienced clinical psychologist. Trial participants also received TAU (comprehensive case management).

### Outcomes

The primary outcome was self-reported personal recovery goals at 15-weeks post randomization, measured in a structured way with the GAS weighted *T*-score;^33–35^ following a recent review.^36^ GAS is sensitive to change, has been the primary outcome for both pharmacological and psychological interventions in psychiatric disorders, and measures cognitive rehabilitation outcomes^37^ including CR.^38^ A baseline participant interview to establish up to 3 goals weighted on importance and difficulty following the scoring manual. Secondary outcomes were: Social and Occupational Functioning (SOFAS^39^); Total hours in structured activity (Time Use Survey;^40^); Negative symptoms (CAINS total score;^41,42^); a composite cognitive score (CANTAB tests:^43^ attention switching, paired visual information processing, reaction time, one touch stocking (testing spatial planning and working memory), spatial working memory, paired associate learning), as well as the Rey auditory verbal learning task, Wisconsin card sorting test, and the digit span task from the Wechsler Adult Intelligence Scale (see Supplementary p3s-4s); Self-esteem total score (Rosenberg Self Esteem Scale^44^); We also collected context information including socio-demographic information and symptoms (Positive and Negative Symptom Scale, PANSS^45^). Cost-effectiveness was estimated with both Quality Adjusted Life Years (QALYs^46^) derived from the EuroQOL-5D-5L and the use of services from the Client Service Receipt Inventory^47^ (see Supplementary for further detail).

### Adverse Events

Adverse and serious events (see Supplementary for definitions) were reported to the trial clinician who assessed their importance and association with the trial and sent a report to the chair of the IDMC for final categorization.

### Service User Involvement

Patient involvement is associated with study success^48^ and so we consulted people with experience of using mental health services at every trial stage, including the study question, primary outcome, design, protocol wording, information sheet, and consent form, in addition to consulting clinicians and carers. We ran focus groups to develop study leaflets to address the sensitive issue of explaining cognitive difficulties. We continued to involve service users as advisors (Patient Advisory Board) who were also critical reviewers, with some being authors of this publication.

### Statistical Analysis

We analyzed the characteristics of people finally entering the trial and compared them descriptively with those from large observational studies of EIS to understand whether the sample was representative of those attending EIS.

### Statistical Power

Following the interim analysis, power was recalculated using an expected 438 participants (see Supplementary p7s for initial calculation). Conventional 2-tailed significance, with 80% endpoint and follow-up data, a plausible correlation structure (0.5 correlation between follow-up measures, 0.2 between baseline and follow-up), but making no allowance for clustering, produced 79% power for an effect size of 0.3 for the Group vs One-to-One arm comparison. Since the focus of therapy was narrowly based on CR activity, both therapist and group interaction variance were expected to be negligible. Site, as a randomization stratifier, was included within the analyses.

An interim intention-to-treat (ITT) analysis, overseen by the IDMC, considered closing trial arms based on: (1) Treatment engagement: > 50% of individuals receiving less than 5 therapy hours, (2) Cost-effectiveness: > £500 for a one-point increase in cognition or one hour of structured activity (a reasonable cost adopted from a previous trial^49^), (3) Participant satisfaction: <25% of participants satisfied with therapy. Designed for 175 participants, it was carried out early with 100, to maximize power for informative contrasts.

#### Primary Analysis.

ITT analyses included all randomized participants, irrespective of the amount of therapy received, using Stata version 15. IDMC recommended three informative contrasts for future clinical service design prioritized to minimize false positive results. The sequence was: (1) determine any difference in the therapist-supported arms (Group vs One-to-One) and if no difference then arms are combined, (2) test whether independent CR improved the primary outcome (Independent vs TAU), and finally (3) a comparison of the combined therapist arms with TAU to consider the overall treatment effect. These pre-specified analyses (see Supplementary statistical analysis plan) were applied to the primary and all secondary outcomes with no adjustment for multiple contrasts. The contrasts specified show whether one of the therapist-supported modes was likely to be more beneficial and whether the least expensive treatment (contrast 2) could also be considered.

A linear mixed model estimated the mean weighted GAS T-score difference between arms at 15 weeks. Independent variables were treatment arm, time (post therapy or follow-up), time by treatment arm interaction, recruitment period (pre- or post-interim analysis), baseline GAS T-score, recruitment site, and a random patient-specific intercept. A dummy indicator for baseline missingness^50^ was included as a covariate. Standardized effect sizes were reported using a standard deviation of 10 in the GAS scoring guide.

Sensitivity analyses assessed, by simulation, the effect of closing arms following the interim analysis, under 2 scenarios (1) no treatment difference and (2) naïve effect estimates for the primary outcome. Additional analyses examined: (1) assumed lack of clustering by site/group, (2) missing at random assumption, and (3) effect of noncompliance to visit windows (see Supplementary).

An additional analysis estimated the effect of receiving treatment (CR hours) on the primary outcome. As those who received more CR were likely to be different from those who did little, we used random treatment assignment as an instrumental variable^51^ and assumed a common effect per hour of CR across the 3 active treatment arms. Estimated using the *sem* command in Stata, the model included site and baseline GAS as covariates in both stages of the instrumental variable regression to estimate the effect of an hour of CR.

#### Secondary Analysis.

These mirrored the primary analyses, but standardized effect sizes (Cohen’s *d*) were calculated by dividing mean differences by the pooled baseline sample standard deviation. The number of hours of structured activity was first log-transformed.

### Health Economic Analyses

The health economic analyses followed standard procedures. EuroQOL-5D-5L ratings were converted to an EQ-5D-3L tariff using the established crosswalk method.^52^ QALYs were calculated using the area under the curve methods and were compared between groups while controlling for baseline utility.^53^ Unit costs were based on the Personal Social Services Research Unit 2020 costs and NHS Improvement 2018–2019 reference costs.^46,54^ As cost data are skewed, the analysis used non-parametric bootstrapping (1000 replications) to generate 95% confidence intervals around the mean differences in costs and outcomes between the groups at each time point. For the main analysis, NHS/PSS costs and QALYs were adjusted for baseline costs/baseline EQ5D-3L scores, trial arm, site, and period. The secondary analyses were the same except included a dummy indicator for baseline missingness.^50^ The 3 cost-effectiveness analyses were Group CR vs TAU, One-to-One CR vs TAU; and Group CR vs One-to-One with QALYs as the primary and GAS scores as the secondary outcome and included all randomized participants. Decisions about cost-effectiveness are based on cost-effectiveness acceptability curves (CEAC) with the key issue being how likely a treatment is to be cost-effective not standard significant differences between arms.

### Role of the Funding Source

The funder of the study had no role in study design, data collection, data analysis, data interpretation, or writing of the report.

## Results

**Fig. 1.**
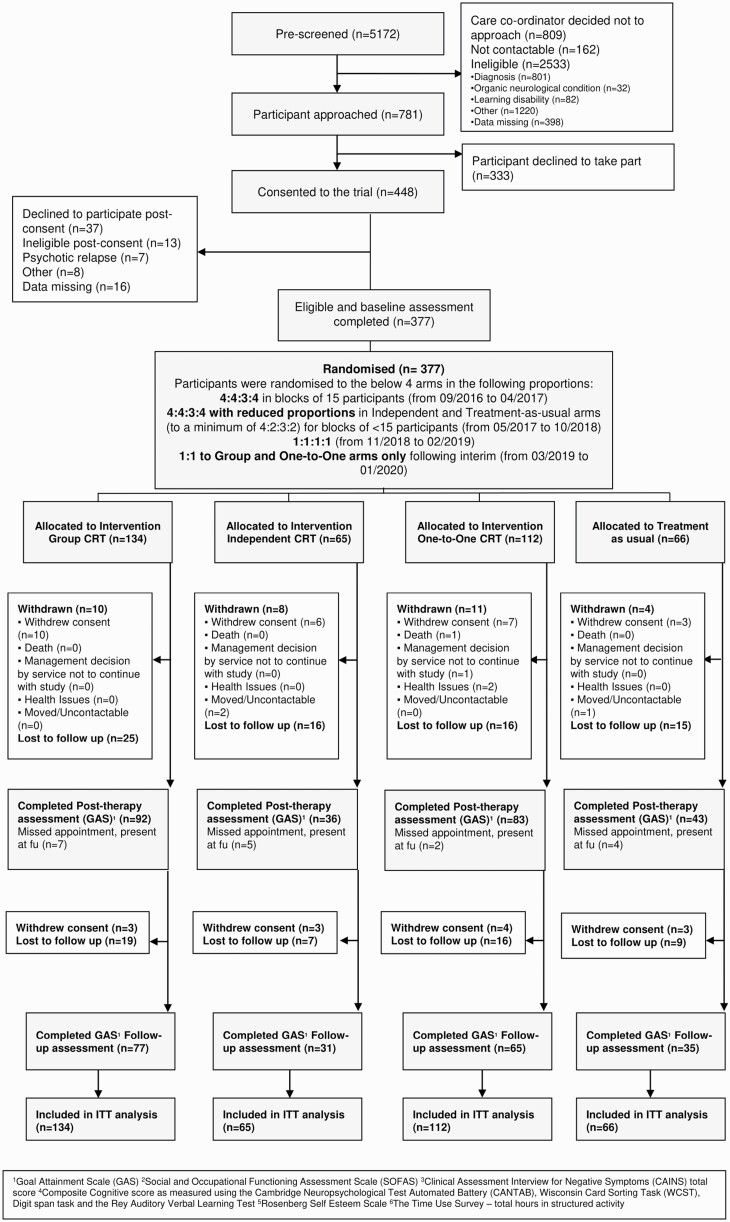
Consort flow chart.

The Consort Flow diagram (figure 1) shows 448 eligible participants; 71 were excluded before randomization, leaving 377 participants (65 Independent, 134 Group, 112 One-to-One, 66 TAU). All those with at least one post therapy or follow-up GAS score were included in the primary analysis (Independent-41, Group-99, One-to-One-85, TAU-47).

The first randomization was on September 14, 2016 and last on January 9, 2020 with the last assessment on October 26, 2020. The Independent arm failed the engagement criterion (50% received less than 5 h) and was the poorest for cost-effectiveness in the interim analysis. The IDMC recommended dropping it and TAU was also dropped because CR had been added to UK NICE guidance.

The sample was predominantly male with a mean age of 26 years. Most were single and largely unemployed. Symptom scores (PANSS and CAINS) were in line with those presenting to EIS.^55,56^ Their pre morbid IQ, current IQ, and duration of untreated psychosis were almost identical to previous large UK EIS studies.^57,58^ Our study sample showed a decrease from pre morbid (98.09) to current IQ (88.18) which is almost identical to changes noted in UK EI services^57^ (pre morbid IQ 95.78, current IQ 88.17,^59^ mean current IQ 84.16, pre morbid IQ 95.82), but also from an international sample of people with schizophrenia^60^ (pre morbid IQ 95.82, current IQ 84.16).

Baseline socio-demographic and clinical data (see table 1) were balanced across treatment arms as expected from randomization. Nine hundred and sixty-four GAS personal goals were identified at baseline, falling into seven categories (Daily Life Skills N285, Employment N107, Education N97, Health and Wellbeing N133, Relationships N133, Recreation/Hobbies N206 and Other eg, spiritual N3: see Supplementary table 1s for examples). The median dose of antipsychotic medication (converted to chlorpromazine equivalents following Leucht et al^61^) was 200 mg post therapy, with 31% of participants reducing and 18% increasing their total dose during the trial (Supplementary table 2s).

**Table 1.** Baseline Characteristics

	Group (*n* = 134)	Independent (*n* = 65)	One-to-One (*n* = 112)	TAU (*n* = 66)	All Participants (*n* = 377)
Age at consent Mean (SD)	25.19 (5.91)	25.92 (5.56)	26.39 (6.72)	25.14 (5.55)	25.67 (6.05)
Sex Female *N* (%)	44 (32.8%)	18 (27.7%)	29 (25.9%)	11 (16.7%)	102 (27.1%)
Ethnicity White *N* (%)	59 (44.0%)	32 (49.2%)	57 (50.9%)	37 (56.1%)	185 (49.1%)
Employment status Full-time education or employed *N* (%)	47 (35.0%)	18 (27.7%)	35 (31.2%)	23 (34.8%)	123 (32.6%)
Living situation Own property (private, rented) or parental home *N* (%)	119 (88.9%)	53 (81.6%)	92 (82.2%)	57 (86.3%)	321 (85.2%)
Relationship status Single *N* (%)	120 (89.6%)	54 (83.1%)	98 (87.5%)	59 (89.4%)	331 (87.8%)
WTAR Standard score Mean (SD)	99.19 (17.05)	97.19 (17.55)	99.00 (16.80)	95.22 (18.22)	98.09 (17.27)
WASI II Estimate IQ (FSIQ) score Mean (SD)	88.81 (15.24)	88.51 (18.95)	87.79 (16.98)	87.23 (17.72)	88.18 (16.82)
PANSS Total score Mean (SD)	55.24 (14.19)	59.66 (19.18)	57.35 (16.45)	55.64 (14.14)	56.70 (15.84)
CAINS Total score Mean (SD)	17.42 (9.31)	18.65 (9.68)	18.62 (9.56)	17.25 (8.44)	17.95 (9.29)
Antipsychotic dosage (converted to chlorpromazine equivalents) Median (Upper–Lower quartiles)	200 (100–301)	240 (120–350)	300·00 (150–450)	180 (59–300)	200 (100–370)

### Which treatment arms provide the most benefit?

**Fig. 2.**
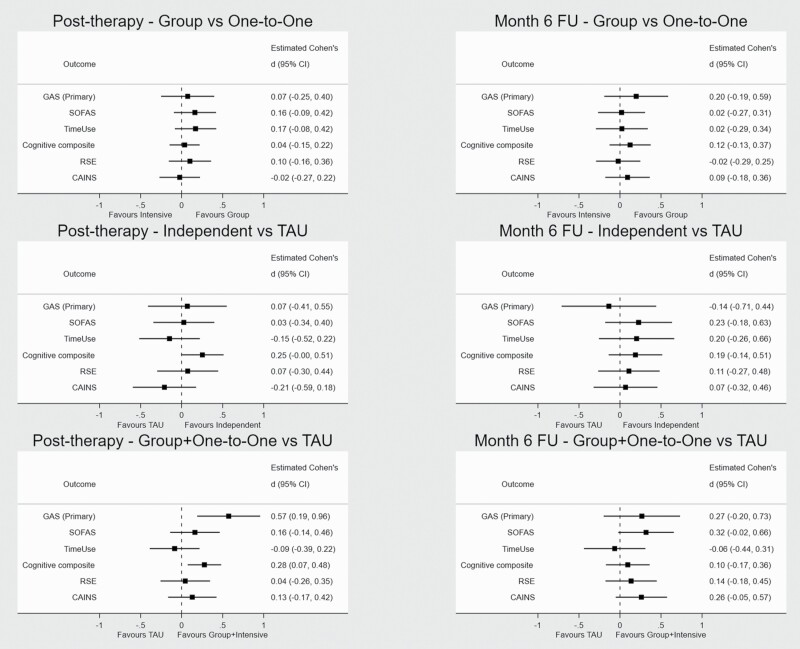
Forest plots for primary and secondary results at each of the contrasts for the 3-month post therapy and 6-month follow-up time points.

Supplementary tables 7s–16s provide summary information for all primary and secondary outcomes. Table 2 shows the results for the primary and secondary outcomes using the pre-specified contrasts at post therapy and at 6-month (post therapy) follow-up. Higher scores indicate a better outcome, except for the CAINS. Forest plots show standardized effects for primary and secondary outcomes for each contrast (figure 2). There was no difference between Group and One-to-One arm, or between TAU and the Independent arm for the GAS-T score. The pooled Group + One-to-One arms showed more benefit than TAU at post therapy (mean pooled GAS T-scores 5.7 points higher, 1.9–9.6 95% CI, Cohen’s *d* 0.57, 0.19–0.96 95% CI, *P* = .003).

**Table 2.** Primary and Secondary Outcome Results

Outcome	Contrast	Post therapy			6-month (post therapy) Follow-up		
		Estimated Mean Difference	*P*-value	95% CI	Estimated Mean Difference	*P*-value	95% CI
GAS T-score^1^	Group vs One-to-One	0.737	.655	−2.500, 3.975	1.975	.319	−1.913, 5.863
	Independent vs TAU	0.695	.777	−4.104, 5.493	−1.353	.645	−7.112, 4.407
	**Group + One-to-One vs TAU**	**5**.**734**	**.003**	**1.898, 9·571**	2.665	.262	−1.988, 7.319
Global cognition composite score^2^	Group vs One-to-One	0.192	.699	−0.785, 1.170	0.659	.333	−0.675, 1.992
	Independent vs TAU	1.348	.054	−0.026, 2.722	1.005	.257	−0.733, 2.744
	**Group + One-to-One vs TAU**	**1**.**479**	**.008**	**0.395, 2.564**	0.507	.484	−0.912, 1.926
SOFAS score	Group vs One-to-One	2.395	.217	−1.405, 6.194	0.287	.894	−3.952, 4.526
	Independent vs TAU	0.400	.887	−5.100, 5.901	3.363	.274	−2.657, 9.384
	Group + One-to-One vs TAU	2.397	.293	−2.073, 6.867	4.701	.066	−0.314, 9.716
Time Use (hours per week)	Group vs One-to-One	0.103	.188	−0.051, 0·257	0.014	.887	−0.179, 0.207
	Independent vs TAU	−0.090	.433	−0.315, 0.135	0.123	.387	−0.156, 0.403
	Group + One-to-One vs TAU	−0.052	.581	−0.237, 0.133	−0.039	.735	−0.267, 0.189
CAINS score	Group vs One-to-One	0.218	.852	−2.072, 2.508	−0.855	.505	−3.368, 1.658
	Independent vs TAU	1.945	.287	−1.633, 5.523	−0.630	.732	−4.240, 2.981
	Group + One-to-One vs TAU	−1.205	.389	−3.946, 1.536	−2.422	.102	−5.327, 0.483
RSE score	Group vs One-to-One	0.571	.440	−0.879, 2.021	−0.122	.875	−1.643, 1.398
	Independent vs TAU	0.415	.698	−1.680, 2.510	0.614	.568	−1.496, 2.725
	Group + One-to-One vs TAU	0.249	.774	−1.452, 1.949	0.766	.393	−0.992, 2.524

*Note*: Results in bold indicate treatment estimates that were statistically significant using a *P* < .05 threshold.

GAS, Goal Attainment Scale; SOFAS, Social and Occupational Functioning Assessment Scale; CAINS, Clinical Assessment Interview for Negative Symptoms; CANTAB, Cambridge Neuropsychological Test Automated Battery; RSE, Rosenberg Self Esteem Scale.

^1^GAS T-score calculated using formula $50\;+\;\frac{10\overset{}{\sum }w_{i}x_{i}}{{\left[(1-\rho )\overset{}{\sum }w_{i}^{2}+\;\rho (\overset{}{\sum }{\left(w_{i}\right)}^{2}\right]}^{1/2}}$with *w*_*i*_ = the weight assigned to the ith goal; a product of participants perceived goal importance (rated 1–3) and difficulty (1–3) *x*_*i*_ = the numerical rating achieved for the *i*th goal (between –2 and + 2) and *ρ* = 0.3 as recommended by the GAS guide. The score was calculated optimally for 3 goals but also for 1 or 2.

^2^The global cognition composite score includes CANTAB tests (Attention switching, Rapid visual information processing continuous performance, Simple and 5 choice reaction time, “One touch Stockings of Cambridge” Test of Planning, Spatial Working Memory) and the Rey Auditory Verbal Learning Test (RAVLT), Wisconsin Card Sorting Task (WCST) and Wechsler Adult Intelligence Scale (WAIS) Digit span. Some components were reverse scored and/or transformed to be approximately normally distributed. *Z*-scores were calculated, and these were then trimmed, to 3 or −3 before summing to give a composite score.

A preliminary homogeneity test indicated no difference in GAS benefit per hour of CR by delivery method. The instrumental variable analysis (ignoring method of delivery) estimated that the endpoint GAS T-score increased by 2.81 (0.90–4.71 95% CI, *P* = .004) post therapy or a 0.28 effect size (Cohens *d*, 0.09–0.47 95% CI) for each 10 h of CR. This reduces to 1.73 (−0.29 to 3.76 95% CI, Cohens *d* 0.17, −0.03 to 0.38 95% CI, *P* = .093) at follow-up.

The cognition composite score for the Group + One-to-One vs TAU comparison at post therapy also showed a benefit, with a small to medium-sized effect (mean increase of 1.48 points, 0.40–2.56 95% CI, Cohen’s *d* 0.28, 0.07–0.48 95% CI, *P* = .008).

### Safety Assessment

Ninety-five adverse events (AE) and 59 serious adverse events (SAEs) were reported. Two AEs were related to the intervention (hearing voices from the CR computer; sending abusive texts to the therapist about compensation) but no SAE was trial related. SAEs were: 2 deaths, 55 mental state deteriorations requiring urgent assessment, and 2 medical hospital admissions (Supplementary tables 5s and 6s). AEs and SAEs were relatively balanced across trial arms with an average of 0.16 SAEs per participant (range 0.10–0.19).

**Adherence:**

Therapy dropout was considered as less than 6 sessions, and although many received 1 therapy session (92.6%), a substantial number failed the 6-session threshold (45% Independent, 37.6% Group, 21.6% One-to-One). When dropouts were removed there was little difference between arms in the proportion receiving a 20-session minimum dose defined in the White Paper (42.9% Independent, 48.2% Group, and 47.1% One-to-One, see Supplementary table 4s).

Sensitivity analyses demonstrated little to no bias introduced by dropping arms or other potential sources of bias (see Supplementary tables 19s–22s and figure 1s).

Resource use, EQ5D-3L and GAS scores for the health economic analyses are in Supplementary tables 23s and 24s. Table 3 shows the NHS costs for each arm at each timepoint and the complete case analysis including the outcomes (QALYs; GAS score), costs (NHS PSS perspective), and cost-effectiveness of Group vs One-to-One, Group vs TAU and One-to-One vs TAU).

**Table 3.** Total Costs (NHS Perspective) and Complete Case Analysis: NHS PSS Costs, Outcome, and Cost-Effectiveness Results

Total costs (NHS perspective)								
Variables: costs (£)	Group		Independent		One-to-One		TAU	
	*N*	Mean (sd)	*N*	Mean (sd)	*N*	Mean (sd)	*N*	Mean (sd)
Intervention	133	266 (989)	63	24 (11)	111	740 (375)	66	0 (0)
Baseline: NHS PSS costs^1^	134	5542 (12 274)	64	5095 (10 139)	110	4943 (10 749)	66	3365 (6648)
Post Therapy: NHS PSS costs^1^	104	839 (1984)	48	1433 (3199)	92	1070 (2866)	54	1875 (5818)
Post Therapy: NHS PSS costs^1^ + Intervention Costs	104	1125 (1975)	48	1456 (3201)	92	1854 (2838)	54	1875 (5818)
Follow-up: NHS PSS costs^1^	89	2201 (6689)	45	2391 (4980)	82	1686 (4305)	51	2928 (7770)
Complete case analysis: NHS PSS costs, QALY outcome, and cost-effectiveness results								
Group vs One-to-One	(*n* = 64)		(*n* = 58)		Incremental cost/QALY gained (95% CI)^2^			
NHS PSS costs^1^	2960 (6626)		2564 (2487)		150 (−1132 to 1905)			
QALYs^3^	0.5998 (0.1059)		0.5601 (0.1661)		0.0057 (−0.023 to 0.0392)			
NHS/PSS perspective: costs (£) per QALY gain (ICER)^4^					26 383			
Group vs TAU	(*n* = 64)		(*n* = 30)		Incremental cost/QALY gained (95% CI)^2^			
NHS PSS costs^1^	2960 (6626)		2724 (4974)		257 (−1694 to 2615)			
QALYs^3^	0.5998 (0.1059)		0.5046 (0.1954)		0.0597 (0.0122–0.1005)			
NHS/PSS perspective: costs (£) per QALY gain (ICER)^4^					4306			
One-to-One vs TAU	(*n* = 58)		(*n* = 30)		Incremental cost/QALY gained (95% CI)^2^			
NHS PSS costs^1^	2564 (2487)		2724 (4974)		260 (−1654 to 2239)			
QALYs^3^	0.5601 (0.1661)		0.5046 (0.1954)		0.0821 (0.0421–0.1219)			
NHS/PSS perspective: costs (£) per QALY gain (ICER)^4^					3170			
Complete case analysis: NHS PSS costs, GAS Score outcome, and cost-effectiveness results								
Group vs One-to-One	(*n* = 70)		(*n* = 62)		Incremental cost/GAS score unit gained^2^			
NHS PSS costs^1^	3012 (6478)		2578 (2582)		195 (−1026 to 1751)			
GAS Score	54.54 (11.91)		53.09 (11.01)		1.59 (−2.26 to 5.17)			
NHS/PSS perspective: costs (£) per additional unit on the GAS (ICER)^4^					123			
Group vs TAU	(*n* = 70)		(*n* = 32)		Incremental cost/GAS score unit gained^2^			
NHS PSS costs^1^	3012 (6478)		2916 (5088)		328 (−1770 to 2870)			
GAS Score	54.54 (11.91)		51.12 (16.28)		4·29 (−1.93 to 10.36)			
NHS/PSS perspective: costs (£) per additional unit on the GAS (ICER)^4^					76			
One-to-One vs TAU	(*n* = 62)		(*n* = 32)		Incremental cost/GAS score unit gained^2^			
NHS PSS costs^1^	2578 (2582)		2916 (5088)		157 (−1556 to 2155)			
GAS Score	53.09 (11.01)		51.12 (16.28)		2.33 (−3.91 to 9.18)			
NHS/PSS perspective: costs (£) per additional unit on the GAS (ICER)^4^					67			

The costs per QALY for Group or One-to-One vs TAU were £4306 and £3170, respectively. There was uncertainty around the results (ie, the probability of cost-effectiveness at £20 000 per QALY (National Institute for Health and Care Excellence (NICE)^62^), shown in CEAC (ICER; see Supplementary figure 3s). The ICER for Group vs One-to-One was £26 383 per QALY gained. Despite less cost for the group intervention, this large difference is mainly accounted for by inpatient and other costs in the group arm.

## Discussion

Cognitive remediation provided either in a Group or One-to-One was more beneficial at post treatment than TAU, but the benefits were reduced at follow-up. There were few differences between these CR methods in terms of costs or cost-effectiveness, and the treatment costs (therapist time) in comparison to the overall health service costs. Both types of provision are therefore recommended. The interventions may entail extra initial investment compared to usual care. However, the health economic results show that overall costs do not differ much from usual care due to cost offsets elsewhere in the system. This is crucial to consider when investigating potentially expensive interventions. QALYs were significantly higher compared to usual care and the probability of either being more cost-effective than usual care was high.

Anecdotal evidence while we were recruiting suggested that the group option was less popular, and there were more people who dropped out of this condition suggesting that more encouragement might be needed to engage in this intervention method. After removing dropouts there were few adherence differences or meeting the minimal 20-session dose between group and one-to-one. This suggests that, once people were engaged, group treatment was equally acceptable (Supplementary table 4s). The choice should therefore be made by the patient themselves if both are offered.

Fewer therapy hours may have contributed to the poor results in the independent arm as they received fewer sessions and hours of therapy than the other CR arms (Supplementary table 4s). As suggested in the White Paper and in meta-analyses^8,9,14^ encouragement by therapists may help adherence and the transfer of gains to functional outcomes. More formal therapist input was associated with treatment adherence, and this clearly affects therapeutic benefit. Therapy hours were constrained by our 12-week intervention window as sessions missed were not reinstated which might explain the lower number of therapy hours compared to other studies.

While the cost and QALY differences were not statistically significant between the treatment arms, the approach used in the economic analyses focuses on the probability that one intervention is more cost-effective than another. Here we found that both Group and One-to-One had a high probability of being more cost-effective than TAU, and the corresponding ICERs were below the lower threshold used by UK NICE (£20 000) for the adoption of a new NHS therapy. The ICER for Group CR vs One-to-one was substantially higher although below the upper threshold of £30 000.

The CR benefits wane, and although present, are no longer statistically significant at 6-month follow-up. We embedded the transfer of skills to real-world activities within the CIRCuiTS software with exercises for using a bus, cooking, or shopping as well as homework activities to aid transfer. However, COVID-19 might have had an impact on outcomes for a minority as opportunities to fulfill some GAS-defined recovery goals were unavailable when social distancing and lockdown were implemented in the United Kingdom. We had 25 post therapy (17 Group, 11 One-to-One) and 40 (18 Group and 22 One-to-One) follow-up assessments that occurred after the beginning of the pandemic (March 16, 2020). These results are therefore likely to be the minimum rather than the maximum of what might have been achieved. In addition we only included a handover note to the local EIS team on CR outcomes, and perhaps if we had included joint sessions with other healthcare professionals (like the Thinking Skills for Work programme^63^) or provided intermittent sessions over the follow-up (as suggested by our Patient Advisory Board), then the benefits might have been maintained.

### Strengths and Limitations

This is the largest study of CR in people with a diagnosis of first-episode non-affective psychosis. Individuals were excluded at the prescreen or screening stages that might have affected confidence in the results, although the sample characteristics were remarkably similar to those of previous observational studies. As this study considered the therapist contact needed for wider implementation we chose a personalized measure of outcome—the GAS—favored by clinical staff and service users to record an individual’s functional recovery targets. This outcome requires rater consistency, although not more so than symptom measures such as PANSS. To ensure consistency we trained and supervised all these ratings that were made blind to group allocation. Missing outcome data could be a potential bias, but our broad sensitivity analyses suggest that our results are robust. While early slow recruitment required changes to randomization, all recruits were randomly allocated and balanced in characteristics suggesting no bias was introduced. We carried out the study in Early Intervention Services which have access to a wide range of recovery services and so the results may not be applicable to those in longer-stay services although this was a stringent test of any additional therapy added to case management.

## Conclusions

The results suggest that providing therapist-led Group and One-to-One CR can improve the prospects for personal functional recovery in early psychosis, and both types of provision are cost-effective. Therapists seem to increase adherence which then increases the CR benefits. Future studies should investigate whether patient characteristics can inform the choice of group or one-to-one therapy.

## Supplementary Material

Supplementary material is available at https://academic.oup.com/schizophreniabulletin/.

sbac214_suppl_Supplementary_Material
