# Supplementary material for: Cognitive Remediation Works But How Should We Provide It? An Adaptive Randomized Controlled Trial of Delivery Methods Using a Patient Nominated Recovery Outcome in First-Episode Participants
Source: Schizophr Bull. 2023 Mar 3;49(3):614–25. doi: 10.1093/schbul/sbac214 (PMC10154711; doi:10.1093/schbul/sbac214)
Supplement: sbac214_suppl_Supplementary_Material [file sbac214_suppl_supplementary_material.docx]

**Supplementary information**

**CONTENTS**

**Page**

**Procedure**

1. Recruitment and consent 2

2. Assessments 2

3. Intervention modes 5

4. Randomisation changes 6

5. Participant Inclusion Criteria Changes 7

6. Statistical Supplementary Data 7

7. Health economic analyses 9

**Results**

8. Basic information 10

9. Statistical output supplementary information 23

10. Health economic supplementary data 27

11. e-references 49

**Statistical Analysis Plan** 54

**Data Sharing Statement** 80

**Procedure**

**1. Recruitment and consent**

Recruitment process: (i) Early psychosis clinicians were asked to identify patients with a non-affective psychosis (i.e. not bipolar disorder or major depression with psychosis); (ii) Early psychosis psychiatrists confirmed whether identified patients were likely to have a diagnosis of schizophrenia, schizo-affective or schizophreniform disorder; (iii) Early psychosis clinicians approached patients individually to ascertain permission for the research team to approach them unless other systems were in place e.g. consent for contact agreements but even in these situations clinicians provided permission to approach; (iv) Written informed consent was obtained by the research team; (v) The Mini International Neuropsychiatric Interview, sections A ‘Major depressive episode’, D ‘Manic/Hypomanic episode’ and L ‘Psychotic disorders’(Sheehan et al. 1998) were applied to confirm the diagnosis (completed by a trained research worker) . (vi) Where there was ambiguity over the diagnosis, researchers looked at participants medical records and spoke with their team doctor (or another member of the clinical team); (vii) Where there was still ambiguity about the eligibility, a final decision was made by the trial team (lead investigators). We mainly recruited within EIS but could include individuals who were within five years of their psychosis onset. However, during the trial this was not necessary as EIS provided enough participants to be included.

Recruitment was carried out in 6 sites each containing different numbers of NHS Trusts that represent varied populations including different ethnicities. We had 7 inner city, 5 mixed urban/suburban and 2 suburban/rural communities. All participants were recruited from Early Intervention Services within the Trusts.

**NHS Trusts involved**: South London and Maudsley NHS Foundation Trust, Camden and Islington NHS Foundation Trust, South West London and St George’s Mental Health NHS Foundation Trust, Barnet Enfield Haringey Mental Health NHS Trust, North East London NHS Foundation Trust, East London NHS Foundation Trust, Cambridgeshire and Peterborough NHS Foundation Trust, Norfolk and Suffolk Mental Health NHS Foundation Trust, Coventry and Warwickshire Partnerships NHS Trust, Birmingham and Solihull Mental Health NHS Foundation Trust, Sussex Partnership NHS Foundation Trust, Birmingham Women’s and Children’s NHS Foundation Trust

**2. Assessments**

**Background contextual measures**

(i) ***Wechsler Test of Adult Reading*** (WTAR; Wechsler 2001) estimates premorbid intellectual functioning. It measures a participant’s ability to read words with atypical pronunciations. WTAR scores are highly correlated with measures of verbal IQ, comprehension and full-scale IQ (Spreen and Strauss, 2006).

(ii) ***Wechsler Abbreviated Scale of Intelligence II*** (WASI– II (Wechsler 1997)) is a brief measure of current intellectual function which uses the verbal comprehension index (Vocabulary/Similarities subtests) and perceptual reasoning index (Matrix Reasoning/Block Design subtests) to estimate full-scale IQ.

**Primary Outcome**

***The Goal Attainment Scale (GAS)*** is a self-report measure which assesses the extent to which an individual’s goals are achieved following an intervention. It is widely used in psychosocial interventions (Bartle et al, 1966; Kiresuk and Sherman, 1968; Rockwood,1997), is reliable and comparable to researcher reports (Kiresuk, Smith et al. 1994, Turner-Stokes 2009). GAS scores are calculated by first identifying goals through an interview with the participant at baseline and establishing an agreed set of priority goal areas. The goals are weighted based on importance and difficulty using the procedure identified in the scoring manual.

**Rationale for using goal attainment scaling (GAS) as primary outcome.**

Clinical trials of cognitive remediation in psychosis assess functional outcome with scales that use self-report, clinician observation or functional capacity (Lejeune et al 2021). Functional outcome derived from personal aspirations of individual participants has been tested widely, including in cognitive remediation studies(Tabak et al. 2015; Shefler, Canetti, and Wiseman 2001; Thomas and Rusten 2019; de Beurs et al. 1993; Strawbridge et al. 2021). We adopted this approach using goal attainment scaling (GAS) as the primary outcome measure based on evidence that it is reliable and sensitive to change in clinical trials and has greater face validity than more global assessments for establishing improvement in function (Stolee et al 1999).

Goal attainment scaling has been traditionally used in physical and cognitive rehabilitation programmes (Rockwood et al., 1997; Turner-Stokes, 2009). It has also been shown to be a sensitive outcome measure in observational studies of patients with cognitive impairment such as mild dementia, acquired brain injury and multiple sclerosis (Bouwens et al. 2008; Chew et al., 2015; Evans, 2012; Rannisto et al., 2015). In randomised controlled trials, GAS has been shown to be sensitive to change and more so than other measures of daily activity (Rockwood et al., 2003) and has been used as the primary outcome measure of both pharmacological and psychological interventions in psychiatric disorders (Burton et al., 2021; Rockwood et al, 2006; McCue et al., 2021)

**Secondary outcomes**

**Cognition**

**Tests from the *Cambridge Neuropsychological Test Automated Battery*** (CANTAB, Barnett et al., 2010) are presented on a touchscreen, and mirror the seven domains evaluated by MATRICS (attention/vigilance, working memory spatial, visual learning, speed of processing, verbal learning, social cognition and reasoning/problem solving). Studies find good levels of test-retest reliability: r>0.8 for some measures of visual learning and r>0.7 for spatial working memory and executive functioning (Lowe and Rabbitt 1998).

**A global cognitive score** was calculated from the following cognitive measures from CANTAB subtests and other measures previously shown to be sensitive to CIRCuiTS. Some Items (in italics below) were reverse scored so that lower was the better for all, and some items were transformed to be approximately normally distributed (see below). All items were transformed to Z scores (to give items equal weight). These Z-scores were then trimmed to 3 or -3 (if they exceeded these values) before summing to get a composite score. The composite score was pro-rated (the missing item replaced by mean of other Z-transformed trimmed items) if one of the items was missing and missing otherwise (if 2 or more items missing).

**CANTAB tests and measures used for composite score**

- Attention Switching Task - Total correct responses for congruent and incongruent trials.
- Rapid Visual Information Processing continuous performance test - A prime sensitivity t to the target sequence. This was reverse scored, and log transformed.
- Simple and 5 choice Reaction Time - Median 5 choice reaction time correct. This was reverse scored.
- One Touch Stockings of Cambridge test of planning - Number of problems solved on first choice.
- Spatial Working Memory - Total between search errors. This was reverse scored.
- Paired Associate Learning - Total adjusted errors. This test was reverse scored.

**Individual neuropsychological tests and measures used for composite score**

- Rey Auditory Verbal Learning Test (Rey and Osterreith 1993) – Sum of 1-5 immediate recall trials.
- Wisconsin Card Sorting Task (Heaton RK 1993) – Perseverative errors. This was reverse scored and log transformed.
- Wechsler Adult Intelligence Scale (Wechsler 1997) - Digit span backward raw score

**Symptoms**

**(i) *Positive and Negative Symptom Scale*** (PANSS) is a widely used measure of symptom severity with 30 items each rated on a 7-point scale and is administered as a semi-structured interview. It has high inter-rater, reliability and validity (Bell et al., 1992), criterion and construct validity (Kay, Fiszbein et al. 1987). All raters were trained, and performance monitored approximately every 3 months using patient/actor videos.

**(ii) *Clinical Assessment Interview for Negative Symptoms*** (Blanchard, Gur et al. 2012) was designed for use in trials, to measure negative symptoms. It is administered in an interview. It has seen high convergent and divergent validity, and high internal consistency (Blanchard, Bradshaw et al. 2017).

**Social Function**

(i) ***Time Use Survey*** (Lader, Short et al. 2006) assesses time spent in employment and education, and leisure activities. It is sensitive to change, has good reliability with quality of life but is less confounded with symptoms (Fowler et al, 2009)

**(ii) *Social and Occupational Functioning Assessment Scale*** (Morosini, Magliano et al. 2000))

**(iii) *The Rosenberg Self-Esteem Scale*** (Rosenberg 1965) is a self-report measure of self-esteem for adolescents and young people with good reliability.

**Health Economics**

**(i) *EuroQol Five Dimensions*** The primary outcome measure used in the cost-effectiveness analysis was the quality adjusted life year (QALY) gained, calculated from the EQ-5D. (EQ-5D; EuroQol Group 1990) which is a self-complete measure of five domains: mobility, self-care, usual activities, pain/discomfort, anxiety/depression. Each of these will receive a score of 1, 2, 3, 4, 5 corresponding to no problems, slight problems, moderate problems, severe problems, and extreme problems. Utility scores will be attached to each health state based on these scores which can then be converted into a health state used to calculate utility in terms of a QALY.

**(ii)** ***Client Service Receipt Inventory*** (Beecham and Knapp 1995) records service use over a retrospective period. Services include specific interventions received and inputs from primary and secondary care as well as social care agencies.

**Adverse events**

**An Adverse Event** (AE) is any untoward medical occurrence in a subject taking part in research study, including occurrences which do not necessarily have a causal relationship with the research.

**A Serious Adverse Event** (SAE) is any untoward and unintended response adverse reaction, respectively that:

- results in death
- is life-threatening
- requires hospitalisation or prolongation or existing hospitalisation
- results in persistent or significant disability or incapacity
- consists of a congenital anomaly or birth defect or
- is otherwise considered medically significant by the investigator
- A Suspected Unexpected Serious Adverse Reaction (SUSAR). If an SAE is related and unexpected it is a SUSAR. Related means it resulted from administration of any of the research procedures. Unexpected is when he type of even is not listed in the protocol as an expected outcome.

**3. Intervention modes**

More information on CIRCuiTS can be found on <https://www.circuitstherapyinfo.com/> It was developed by Til Wykes and Clare Reeder based on pedagogical theory that it is important to teach new skills by supporting metacognition as this allows the transfer of cognitive skills to new problems and situations (see Wykes and Reeder, 2005). Previous studies have shown that metacognition is improved with CIRCuiTS (see (Cella et al. 2019; Hyde et al. 2020; Hiekkala-Tiusanen et al. 2019; Van Duin et al. 2021; Thomas and Rusten 2019; Palumbo et al. 2019; Drake et al. 2014)). CIRCuiTS has been tested previously both in studies by the developers and others and have shown improvements in cognition and functioning (e.g(Hyde et al. 2020; Hiekkala-Tiusanen et al. 2019; Van Duin et al. 2021; Thomas and Rusten 2019; Palumbo et al. 2019; Drake et al. 2014).

The chosen intervention modes are those traditionally adopted in different cognitive remediation studies but not compared in a single study.

CIRCuiTS engages cognition, metacognition, and the transfer of these skills to functioning both within the therapy and with the help of a therapist. The therapy includes cognitive tasks (as in other software programmes) but also includes exercises where the cognitive skills (planning, strategy use and metacognitive awareness and regulation) are used to engage with real life activities such as shopping, cooking, and travelling on public transport to aid transfer. It also allows therapeutic conversations about how strategies and planning (metacognition) will help complete the exercises, but as the software has built-in transfer opportunities to daily life activities and toward personal goals it may not need a therapist.

**(a) *One-to-one CR***. Participants receive 10.5 weeks of twice weekly therapy, up to 42 h in total, with sessions lasting between 60 and 180 min, split into three parts: (1) 20–60 min of CR with a therapist; (2) 20–60 min of in-vivo transfer work (i.e., putting CR strategies into real life) with a therapist; (3) 20–60 min of independent CR, set up by the therapist on-site, or off-site in the service user’s own time. A session is ‘valid’ if it lasts a minimum of 20 min.

**(b) *Group CR***. Participants are offered 14 weeks of 3 times weekly group therapy (up to 42 h of CR). Sessions last up to 90 min, with attendance for at least 20 min considered as a completed session. Groups have four participants and one therapist. The sessions begin and end with group activities related to goal setting and metacognition. For the remainder of the session, service users work independently, with the therapist offering help and support on an as-needed basis.

**(c) *Independent CR****.* Participants receive one individual session with the therapist for orientation followed by up to 41 sessions when they work independently (up to 42 h of CR in total). To support the independent sessions, the therapist will offer telephone contact or attendance at drop-in sessions on an as-needed basis to address any questions or problems (but not exceeding 1 h contact time per fortnight). A session is ‘valid’ if it lasts a minimum of 20 min.

**(d) *Treatment as usual.*** This is the set of standard interventions offered by the treating team without restrictions. It involves clinical contact on a daily, weekly or monthly basis depending on recovery as well as educational or employment programmes, other psychological therapies, e.g., cognitive behaviour therapy for psychosis and medical treatments, including drug therapies.

**4. Randomisation changes**

Four different randomisation systems/algorithms were used during the trial:

1. (Version 1, September 2016-April 2017) The initial randomisation system was implemented using the KCTU (King’s Clinical Trials Unit) bespoke web-based randomisation service with randomisation to all 4 arms in blocks of 15 with allocation ratio 4:3:4:4 to the Group CR, One-to-One CR, Independent CR and TAU arms respectively. Randomisation was in blocks of 15 stratified by site, and randomisation only occurred once complete blocks of 15 were randomised. This was to ensure that 4 participants were in the Group CR at each interval; and only 3 participants in the One-to-One CR arm (to maximise use of therapist resources).
2. (Version 2, May 2017 - July 2018) The initial randomisation procedure proved too restrictive as some sites could not recruit 15 participants in a timely fashion. It was decided to allow blocks of 11-15 participants, with numbers being reduced in the Independent CR and TAU arms as necessary (ranging from 4:3:4:4 ratio to a 4:3:2:2 ratio). This was not possible with the KCTU bespoke web-based randomisation service and so the algorithm was programmed by the trial statistician (in Stata v14.0), with random lists being pre-generated for each of the different block sizes. These lists were then administered by the KCTU, who provided the allocations to the sites when randomisation was requested. Only the trial statistician and KCTU had access to these lists, and lists were used sequentially. This ensured allocation concealment. As before these lists were administered within and therefore stratified by site.
3. (Version 3, Nov 2018 - Feb 2019) As there were still difficulties at some sites in recruiting 11 participants at once, the block design was abandoned, and participants were instead randomised individually (with 1:1:1:1 allocation ratio) using the KCTU bespoke web-based randomisation service, with randomly varying blocks and stratified by site.
4. (Version 4, Feb 2019 - Jan 2020) Following the interim analysis, the Independent CR and TAU arms were dropped. The randomisation system was amended to randomise the Group and One-to-One arms only (in 1:1 ratio), with randomly varying blocks and stratified by site.

**5. Participant Inclusion Criteria Changes**:

The original inclusion criteria were: (i) aged 16-35 (ii) using EIS for at least 6 months. With the approval of the Steering Group and DMEC, this was modified to: (i) aged up to 45, (ii) attending an EIS for at least 3 months or within 5 years of their first episode of psychosis. An exclusion criterion was added for clarification purposes: a definitive diagnosis of bipolar disorder.

5172 patients were identified from 6 research sites (covering 11 NHS Trusts) as having non-affective psychosis and a likely diagnosis of schizophrenia, schizo-affective or schizophreniform disorder. 4724 of these patients were excluded prior to being approached based on: Not wanting to take part - care coordinator (n=809), Not wanting to take part - participant (n=333), Not contactable (n=162), and being ineligible (n=2533). 448 patients consented to participate in the trial. Of these, 71 were excluded with 37 declining to participate post consent, 13 being ineligible post-consent, 7 having a psychotic relapse, 8 for other reasons, and 16 due to missing data.

**6. Statistical Supplementary Data**

***a. Initial sample size calculation***

While testing against TAU would be expected to require only a modest sample size, the effect size differences among the active arms are likely to be less substantial. (Freidlin, Korn et al. 2008) suggest no great advantage in accounting for multiple testing in a multi-arm trial, and also that the advantages of a larger TAU arm are more than commonly assumed. Interaction among patients in group delivery is very slight so no allowance for clustering was thought necessary. We have the capacity to recruit 900 patients (from 1500 attending 10 services for 3 years) and have allowed for a 20% drop-out. Using a design with parallel arms of equal size with 180 patients per arm provides approximately 80% power for a simple group ES difference of 0.3. This increases to 91% for outcomes that correlate 0.5 with baseline (both calculated using *sampsi* in Stata).

***b. Interim analysis***

Criteria for dropping a treatment arm in the interim analysis were as follows: 1. Treatment engagement – an arm that has more than 50% of individuals receiving therapy for less than 5 hours; 2. Participant satisfaction –25% of participants disagree with the statement, “Overall I was satisfied with the CIRCuiTS therapy”; 3. Cost-effectiveness –more than £500 for a one-point increase in cognition (visual and verbal memory), one hour of structured activity.

The interim analysis occurred using data up to 09/10/2018, at which point 213 participants had been randomised, and 164 participants (53,38,37,36 respectively for Group, Independent, One-to-One and TAU) had reached the post-therapy timepoint. Of these 100 participants (61%) had follow up data (33,22,25,20 respectively). The DMC reviewed this data at a teleconference on 14/11/2018.

The DMC reported that It was clear that the independent treatment arm failed the criteria for the engagement of participants and performed worst in the interim cost-effectiveness analyses. Therefore, the DMC independent members were unanimous in their support for dropping this treatment arm.

In order to ensure target recruitment, it was decided by the study team and with the agreement of the oversight committee and the patient advisory board to also drop the treatment-as-usual arm. This was due to there already being a good number of participants recruited to this arm. Given the known efficacy of the intervention, the most important comparison was between active treatments (i.e., different modes of delivering CR). As such the TAU arm was dropped and the comparisons between arms amended as presented in the primary paper.

**7. Health economic analyses**

Secondary cost-effectiveness analyses considered a broader perspective including the time spent in employment and contacts with the criminal justice system. We compared costs from this perspective in turn and combined them with QALYs/ GAS scores over both stages.

Cost-effectiveness planes were produced for the primary cost-effectiveness analysis, using 1000 cost outcome pairs (from bootstrapped regression models) to present uncertainty around the ICER estimates in the main analysis. Cost-effectiveness acceptability curves (CEACs) were produced using the bootstrapped results to present the probability of bootstrapped replicates that are cost effective at a range of threshold values.

Deterministic sensitivity analysis was conducted by varying the intervention costs upwards and downwards by 10, 25, and 50%. Unit costs were varied where there was uncertainty around initial estimates, for example using a higher and lower rate for an admission taken from PSSRU unit costs. The following additional sensitivity analyses were considered:

- Intervention costs: Increasing the unit cost of a Band 4 therapist to a Band 5 therapist (£36).
- Training: £500 online training fee plus 23 hours of online training time (varying 20-25 in sensitivity analyses)
- Considering a Senior clinical psychologist 8a level at £69 per hour.
- The cost of licensing will be varied between £25 and £19 per patient.
- Sensitivity analysis will also use the 5L tariffs for the EQ-5D-5L to establish whether there is a substantial difference between the two value sets.

**Results**

**8. Basic information**

**Table 1s Examples of SMART Goals in the GAS scaling**

| **SMART goal** | **Importance** | **Difficulty** |
| --- | --- | --- |
| Plan, prepare and practice keyboard for 30 minutes every other day | 2. Moderately | 1. A little |
| Read psychology books online for 30 minutes per week | 1. A little | 3. Very |
| Plan, prepare or read for homework/classes for 1.5 hours everyday | 3. Very | 2. Moderately |
| Learn to drive by planning and arranging lessons and practice theory test | 3. Very | 2. Moderately |
| Spend 30 minutes 3x per week to plan clothes and to do hair in the morning | 2. Moderately | 1. A little |
| Eat a healthy breakfast 2-3 times per week and get up early | 3. Very | 2. Moderately |
| Contact friends 2x per week for 30 minutes | 2. Moderately | 1. A little |
| Look for plumbing courses for 1 hour per week and update CV | 2. Moderately | 2. Moderately |
| Look for jobs online three times a week for one hour each time. | 1. A little | 2. Moderately |
| Write down distractions and go through a mental ''walking checklist'' | 2. Moderately | 2. Moderately |
| Going to the shops alone | 3. Very | 3. Very |
| Save £100 per month | 3. Very | 2. Moderately |
| Look for work 3x per week for 1 hour | 2. Moderately | 3. Very |
| Plan time to go out 2x per week to look for work and hand out CV | 1. A little | 2. Moderately |
| Eat 3 meals per day and have 5 home cooked meals per week | 3. Very | 2. Moderately |
| 1x a week look online to find events | 2. Moderately | 1. A little |
| Twice a week go to the gym for 2 hours and twice a week read about fitness and nutrition for 2 hours | 3. Very | 2. Moderately |
| Play the guitar 1x a week for 1 hour | 1. A little | 2. Moderately |
| To organise & cook at least 1 healthy meal a day | 3. Very | 2. Moderately |
| To exercise at least 3 times a week | 2. Moderately | 2. Moderately |

**Table 2s Medication data**

| Table 2s Medication Information | | | | |
| --- | --- | --- | --- | --- |
|  | **Independent**  **N = 65** | **Group**  **N = 134** | **One-to-One**  **N = 112** | **TAU**  **N = 66** |
| **Baseline AP converted dose** |  |  |  |  |
| N | 62 | 125 | 106 | 64 |
| Mean (SD) | 247·06 (184·71) | 268·01 (319·08) | 319·87 (233·95) | 284·95 (531·13) |
| Median (LQ-UQ) | 239·52  (119·76-350·15) | 200·00  (100·00-301·20) | 300·00  (150·15-450·45) | 179·64  (58·98-300·00) |
| **Post-therapy AP converted dose** |  |  |  |  |
| N | 33 | 87 | 71 | 39 |
| Mean (SD) | 213·28 (187·84) | 201·72 (228·00) | 287·64 (223·69) | 318·93 (444·13) |
| Median (LQ-UQ) | 200·00  (6·67-300·00) | 160·00  (8·89-300·15) | 225·06  (119·76-450·45) | 266·67  (8·89-364·64) |
| **Change in dose** |  |  |  |  |
| N | 32 | 81 | 70 | 38 |
| Mean (SD) | -54·38 (167·57) | -50·64 (307·32) | -49·94 (190·07) | 57·85 (205·75) |
| Median (LQ-UQ) | 0·00  (-123·15-0·00) | 0·00  (-75·02-0·00) | 0·00  (-89·97-0·00) | 0·00  (0·00-93·75) |

##### **Duration of Untreated Psychosis (DUP)**

Duration of Untreated Psychosis (DUP) was assessed using the relevant sections of the Nottingham Onset Schedule (NOS) and calculated in months. The baseline duration shows some imbalance between treatment arms, with the mean duration for the One-to-One arm being lower than for the Independent arm and with a smaller standard deviation, due to a lower range of data. However, the data is skewed by outliers, with the median being similar throughout the trial arms.

There are a total of 89 missing values across all arms, with similar numbers in the Group and One-to-One arms, which were continued after the interim.

**Table 3s - Duration of untreated psychosis (in months) by trial arm**

| Duration of Untreated Psychosis (months) | Group,  N = 134 | Independent,  N = 65 | One-to-One  N = 112 | TAU,  N = 66 | Overall,  N = 377 |
| --- | --- | --- | --- | --- | --- |
| N (%) | 103 (76.87%) | 49 (75.38%) | 81 (72.32%) | 55 (83.33%) | 288 (76.39%) |
| Mean (SD) | 10.66 (21.21) | 11.14 (22.06) | 5.22 (10.05) | 7.68 (18.83) | 8.64 (18.50) |
| Median (IQR) | 2.07 (0.00, 12.67) | 1.03 (0.00, 8.13) | 1.00 (0.00, 5.03) | 1.03 (0.00, 5.07) | 1.03 (0.00, 7.13) |
| Range | 0.00, 143.03 | 0.00, 93.40 | 0.00, 63.93 | 0.00, 103.53 | 0.00, 143.03 |
| (Missing) | 31 | 16 | 31 | 11 | 89 |

**Table 4s Adherence**

| Table 4s Therapy adherence | | | | |
| --- | --- | --- | --- | --- |
|  | **Independent** | **Group** | **One-to-One** | **Total** |
| **N (%)** | 65 (20·9%) | 134 (43·1%) | 112 (36·0%) | 311 (100·0%) |
| **Valid sessions of therapy attended** Mean (SD) | 11·59 (12·33) | 12·49 (10·87) | 15·59 (10·41) | 13·4 (11·1%) |
| **Number of hours of therapy attended** Mean (SD) | 8·84 (9·90) | 14·45 (12·72) | 19·38 (12·82) | 15·1 (12·8%) |
| **Received at least one therapy session** Yes N (%) | 63 (96·9%) | 122 (91·0%) | 103 (92·0%) | 288 (92·6%) |
| **Received minimum dose of 20 therapy sessions** Yes N (%) | 15 (23·1%) | 40 (29·9%) | 41 (36·6%) | 96 (30·9%) |
| **Drop-out - 5 sessions or less (%)** | 29 (45·3%) | 50 (37·6%) | 24 (21·6%) | 108 (33·4%) |
| **Received 20 session minimum dose after drop-out** | 15 (42·9%) | 40 (48·2%) | 41 (47·1%) | 96 (46·8%) |

**Table 5s Adverse event reporting**

| Table 5s Adverse Events and Serious Adverse Events reporting | | | | | | |
| --- | --- | --- | --- | --- | --- | --- |
| **Trial arm** | **Total AEs** | **Number (%) participants who experienced an AE** | **Mean AEs per participant** | **Total SAEs** | **Number (%) participants experienced an SAE** | **Mean SAEs per participant** |
| Independent (N=65) | 14 | 12 (18·5%) | 0·22 | 10 | 9 (14·1%) | 0·15 |
| Group  (N=134) | 44 | 23 (17·2%) | 0·33 | 26 | 17 (12·7%) | 0·19 |
| One-to-One (N=112) | 21 | 16 (14·3%) | 0·19 | 11 | 9 (8·0%) | 0·10 |
| TAU  (N=66) | 16 | 9 (13·6%) | 0·24 | 12 | 9 (13·6%) | 0·18 |
| Total  (N=377) | 95 | 60 (15·9%) | 0·25 | 59 | 44 (11·7%) | 0·16 |

**Table 6s – Serious Adverse Events (SAE)**

| **Table 6s Serious Adverse Events** | | | | |
| --- | --- | --- | --- | --- |
| **SAE** | **Independent (n=10)** | **Group (n=26)** | **One-to-One (n=11)** | **TAU (n=12)** |
| Deterioration of mental state requiring hospital admission | 5 | 8 | 4 | 5 |
| Deterioration of mental state necessitating urgent assessment | 4 | 18 | 5 | 6 |
| Death | 0 | 0 | 2 | 0 |
| Hospital admission for physical health reasons | 1 | 0 | 0 | 1 |

**Table 7s** – all primary and secondary outcomes by time of assessment

| Table 7s Primary and secondary outcomes by time of assessment  Mean, (SD), n | | | | | |
| --- | --- | --- | --- | --- | --- |
|  | Group  N = 134 | Independent  N = 65 | One-to-One  N = 112 | TAU  N = 66 | Overall  N = 377 |
| **GAS Weighted T-score** | | | | | |
| Baseline | 33·46 (4·58)  n=130 | 33·93, (4·37)  n=64 | 32·48, (5·11)  n=110 | 33·78 (4·39)  n=66 | 33·31 (4·69) n=370 |
| Post-therapy | 52·14 (11·06)  n=92 | 46·74, (9·64)  n=36 | 50·87 (12·01), n=83 | 46·60 (12·20) n=43 | 50·02 (11·56) n=254 |
| Follow up | 53·73 (12·06)  n=77 | 49·97, (11·52)  n=31 | 52·96 (11·43) n=65 | 50·67 (16·28) n=35 | 52·41 (12·60), n=208 |
| **SOFAS score** | | | | | |
| Baseline | 64·14, (15·01)  n=134 | 61·14, (14·88)  n=65 | 64·53 (14·11) n=112 | 63·74 (15·54) n=66 | 63·67 (14·81) n=377 |
| Post-therapy | 68·91, (15·46)  n=86 | 64·31, (15·96)  n=35 | 65·32 (17·69) n=76 | 66·71 (16·27) n=41 | 66·71 (16·41) n=238 |
| Follow up | 69·60, (17·57)  n=70 | 68·52, (15·75)  n=33 | 68·48 (17·63) n=63 | 65·97 (14·69) n=34 | 68·45 (16·77) n=200 |
| **Time Use total hours of structured activity (log hours per week)** | | | | | |
| Baseline | 4·66, (0·54)  n=134 | 4·63, (0·66)  n=65 | 4·56 (0·64) n=112 | 4·75, (0·63)  n=66 | 4·64 (0·61) n=377 |
| Post-therapy | 4·62, (0·52)  n=91 | 4·61, (0·39)  n=37 | 4·49 (0·74)  n=78 | 4·74, (0·48)  n=42 | 4·60, (0·58) n=248 |
| Follow up | 4·63 (0·51),  n=74 | 4·83 (0·72), n=31 | 4·64 (0·67), n=64 | 4·71 (0·53), n=35 | 4·67 (0·60), n=204 |
| **CAINS score** | | | | | |
| Baseline | 17·42 (9·31),  n=134 | 18·65 (9·68), n=65 | 18·62 (9·56), n=109 | 17·25 (8·44), n=65 | 17·95 (9·29), n=373 |
| Post-therapy | 16·78 (9·53),  n=63 | 20·11 (10·21), n=27 | 17·36 (9·85), n=75 | 16·61 (9·18), n=38 | 17·35 (9·66), n=223 |
| Follow up | 14·82 (9·44),  n=65 | 17·38 (10·13), n=29 | 16·54 (9·78), n=59 | 16·82 (9·30), n=34 | 16·12 (9·61), n=187 |
| **Rosenberg Self Esteem score** | | | | | |
| Baseline | 17·22 (5·78),  n=134 | 16·14 (5·68), n=65 | 16·88 (5·09), n=111 | 16·31 (6·22), n=66 | 16·77 (5·64), n=376 |
| Post-therapy | 18·79 (6·12),  n=87 | 16·86 (5·29), n=34 | 17·99 (6·04), n=76 | 17·26 (5·88), n=42 | 17·99 (5·95), n=239 |
| Follow up | 18·82 (5·44),  n=71 | 17·50 (6·02), n=32 | 18·51 (5·89), n=60 | 16·57 (6·15), n=37 | 18·10 (5·82), n=200 |
| **Composite Cognitive Score (composite of 9 Z-transformed items)** | | | | | |
| Baseline | 0·11 (4·92),  n=129 | -0·34 (5·77), n=63 | 0·94 (5·09), n=102 | -0·40 (6·01), n=61 | 0·18 (5·33), n=355 |
| Post-therapy | -0·18 (5·50),  n=66 | 0·84 (5·60), n=29 | 0·61 (5·19), n=66 | -0·53 (4·73), n=36 | 0·17 (5·26), n=197 |
| Follow up | 0·70 (5·04),  n=49 | 0·01 (6·26), n=27 | 0·63 (5·58), n=38 | -0·95 (6·10), n=28 | 0·22 (5·62), n=142 |

##### **Descriptive statistics for the baseline, post-therapy and follow-up scores for all variables which make up the CANTAB composite score**

The CANTAB/cognitive outcome is a composite score of the following 9 cognitive measures, shown in Tables 43 to 52.

Attention Switching Task Total Correct (ASTTC) is the total number of congruent and incongruent trials for which the outcome was a correct response (the participant pressed the correct button within the response window) and is calculated across all assessed trials. Higher scores can be interpreted as better.

| Table 8s Details for the Attention Switching cognitive test from the CANTAB | | | | | |
| --- | --- | --- | --- | --- | --- |
|  | Group  N = 134 | Independent  N = 65 | One-to-One  N = 112 | TAU  N = 66 | Overall, N = 377 |
| **Baseline ASTTC score** | | | | | |
| N | 132 (98.5%) | 63 (96.9%) | 106 (94.6%) | 64 (96.9%) | 365 (96.8%) |
| Mean (SD) | 140.73 (17.38) | 141.27 (21.04) | 145.55 (15.87) | 139.84 (23.65) | 142.07 (18.94) |
| Median (IQR) | 146.00(133.00, 155.00) | 148.00(133.50, 157.00) | 153.00 (136.50, 157.75) | 151.00(127.75, 157.00) | 149.00 (134.00, 156.00) |
| Range | 78.00, 160.00 | 56.00, 160.00 | 98.00, 160.00 | 56.00, 159.00 | 56.00, 160.00 |
| (Missing) | 2 | 2 | 6 | 2 | 12 |
| **Post-therapy ASTTC score** | | | | | |
| N | 71 (52.9%) | 32 (49.2%) | 66 (58.9%) | 38 (57.6%) | 207 (54.9%) |
| Mean (SD) | 142.85 (19.36) | 145.50 (15.63) | 147.36 (16.80) | 145.87 (15.23) | 145.25 (17.27) |
| Median (IQR) | 151.00(137.50, 155.00) | 154.00(137.25, 156.25) | 154.50 (142.25, 158.00) | 150.00(136.75, 158.00) | 152.00 (138.00, 157.00) |
| Range | 68.00, 160.00 | 104.00, 160.00 | 82.00, 160.00 | 92.00, 160.00 | 68.00, 160.00 |
| (Missing) | 63 | 33 | 46 | 28 | 170 |
| **Follow-up ASTTC score** | | | | | |
| N | 50 (37.3%) | 29 (44.6%) | 40 (35.7%) | 33 (50.0%) | 152 (40.3%) |
| Mean (SD) | 146.46 (17.97) | 147.07 (14.60) | 144.72 (18.74) | 140.94 (21.63) | 144.92 (18.42) |
| Median (IQR) | 154.00(146.75, 157.00) | 153.00(140.00, 158.00) | 152.00 (143.75, 157.25) | 148.00(139.00, 155.00) | 152.00 (140.00, 157.00) |
| Range | 82.00, 160.00 | 108.00, 160.00 | 85.00, 160.00 | 68.00, 160.00 | 68.00, 160.00 |
| (Missing) | 84 | 36 | 72 | 33 | 225 |

Rapid Visual Processing A’ (RVPA) is the signal detection measure of a participants sensitivity to the target sequence (string of three numbers), regardless of response tendency (the expected range is 0.00 to 1.00). In essence, this metric is a measure of how good the participant is at detecting target sequences. Higher scores can be interpreted as better.

**Table 9s - Rapid visual information processing (RVIP)**

| Variable | Group, N = 134 | Independent, N = 65 | One-to-One, N = 112 | TAU, N = 66 | Overall, N = 377 |
| --- | --- | --- | --- | --- | --- |
| **Baseline RVPA score** | | | | | |
| N | 130 (97.0%) | 63 (96.9%) | 104 (92.9%) | 62 (93.9%) | 359 (95.2%) |
| Mean (SD) | 0.95 (0.06) | 0.94 (0.08) | 0.96 (0.06) | 0.95 (0.06) | 0.95 (0.06) |
| Median (IQR) | 0.97 (0.93, 0.99) | 0.97 (0.94, 0.99) | 0.98 (0.94, 0.99) | 0.97 (0.93, 0.99) | 0.97 (0.93, 0.99) |
| Range | 0.60, 1.00 | 0.47, 1.00 | 0.61, 1.00 | 0.68, 1.00 | 0.47, 1.00 |
| (Missing) | 4 | 2 | 8 | 4 | 18 |
| **Post-therapy RVPA score** | | | | | |
| N | 70 (52.2%) | 31 (47.7%) | 67 (59.7%) | 38 (57.6%) | 206 (54.6%) |
| Mean (SD) | 0.95 (0.06) | 0.97 (0.03) | 0.96 (0.07) | 0.96 (0.05) | 0.96 (0.06) |
| Median (IQR) | 0.98 (0.94, 0.99) | 0.97 (0.95, 0.99) | 0.98 (0.96, 0.99) | 0.98 (0.96, 0.99) | 0.98 (0.96, 0.99) |
| Range | 0.67, 1.00 | 0.91, 1.00 | 0.47, 1.00 | 0.78, 1.00 | 0.47, 1.00 |
| (Missing) | 64 | 34 | 45 | 28 | 171 |
| **Follow-up RVPA score** | | | | | |
| N | 50 (37.3%) | 29 (44.6%) | 40 (35.7%) | 32 (48.5%) | 151 (40.1%) |
| Mean (SD) | 0.96 (0.06) | 0.97 (0.03) | 0.96 (0.05) | 0.95 (0.06) | 0.96 (0.05) |
| Median (IQR) | 0.98 (0.96, 0.99) | 0.98 (0.95, 0.99) | 0.99 (0.95, 0.99) | 0.97 (0.92, 0.99) | 0.98 (0.95, 0.99) |
| Range | 0.68, 1.00 | 0.86, 1.00 | 0.83, 1.00 | 0.79, 1.00 | 0.68, 1.00 |
| (Missing) | 84 | 36 | 72 | 34 | 226 |

Reaction Time Median Five-Choice Reaction Time is the median duration it took for a participant to release the response button after the presentation of a target stimulus. It is calculated across correct, assessed trials in which the stimulus could appear in any one of five locations. It is measured in milliseconds and a lower score is better.

**Table 10s – Reaction Time Median five-choice reaction time correct (RTIFMDRT)**

| Variable | Group,  N = 134 | Independent,  N = 65 | One-to-One,  N = 112 | TAU,  N = 66 | Overall,  N = 377 |
| --- | --- | --- | --- | --- | --- |
| **Baseline RTIFMDRT score** | | | | | |
| N | 133 (99.3%) | 65 (100.0%) | 107 (95.5%) | 64 (97.0%) | 369 (97.9%) |
| Mean (SD) | 412.92 (76.12) | 411.23 (61.29) | 407.07 (69.04) | 420.34 (124.14) | 412.22 (82.23) |
| Median (IQR) | 397.50 (366.00, 437.00) | 403.00(370.00, 436.00) | 394.00 (366.50, 430.50) | 399.00 (373.12, 435.12) | 398.50 (368.00, 435.00) |
| Range | 306.00, 801.00 | 331.00, 657.00 | 318.00, 849.00 | 304.00, 1,249.00 | 304.00, 1,249.00 |
| (Missing) | 1 | 0 | 5 | 2 | 8 |
| **Post-therapy RTIFMDRT score** | | | | | |
| N | 70 (52.2%) | 32 (49.2%) | 67 (59.8%) | 39 (59.1%) | 208 (55.2%) |
| Mean (SD) | 404.11 (57.22) | 404.58 (56.96) | 403.34 (44.16) | 406.79 (55.08) | 404.44 (52.51) |
| Median (IQR) | 398.25 (361.62, 431.00) | 386.75 (364.88, 428.00) | 391.00(373.75, 427.75) | 398.50 (366.25, 444.50) | 392.75 (367.00, 432.25) |
| Range | 318.00, 585.00 | 325.00, 560.50 | 318.50, 536.00 | 312.00, 579.50 | 312.00, 585.00 |
| (Missing) | 64 | 33 | 45 | 27 | 169 |
| **Follow-up RTIFMDRT score** | | | | | |
| N | 52 (38.8%) | 32 (49.2%) | 39 (34.8%) | 32 (48.5%) | 155 (41.1%) |
| Mean (SD) | 394.14 (51.65) | 394.12 (37.27) | 396.00 (47.94) | 411.47 (85.41) | 398.18 (56.97) |
| Median (IQR) | 380.75 (351.00, 432.50) | 388.75 (368.38, 421.12) | 392.50(364.25, 415.00) | 391.75 (358.25, 439.25) | 388.00 (359.75, 426.25) |
| Range | 303.00, 517.00 | 324.00, 482.00 | 306.50, 550.00 | 316.00, 655.00 | 303.00, 655.00 |
| (Missing) | 82 | 33 | 73 | 34 | 222 |

One touch Stockings of Cambridge (OTSPSFC) is the total number of assessed trials where the participant chose the correct answer on their first attempt. Calculated across all assessed trials. Higher scores can be interpreted as better.

**Table 11s - One touch Stockings of Cambridge (OTSPSFC)**

| Variable | Group,  N = 134 | Independent,  N = 65 | One-to-One,  N = 112 | TAU,  N = 66 | Overall,  N = 377 |
| --- | --- | --- | --- | --- | --- |
| **Baseline OTSPSFC score** | | | | | |
| N | 133 (99.3%) | 64 (98.5%) | 105 (93.8%) | 63 (95.5%) | 133 (35.3%) |
| Mean (SD) | 8.94 (3.39) | 8.34 (3.78) | 9.27 (3.10) | 8.48 (3.57) | 8.94 (3.39) |
| Median (IQR) | 10.00(7.00, 11.00) | 9.00(5.75, 11.00) | 10.00 (8.00, 12.00) | 9.00(6.00, 11.00) | 10.00(7.00, 11.00) |
| Range | 1.00, 15.00 | 0.00, 15.00 | 1.00, 15.00 | 0.00, 14.00 | 1.00, 15.00 |
| (Missing) | 1 | 1 | 7 | 3 | 1 |
| **Post-therapy OTSPSFC score** | | | | | |
| N | 71 (52.9%) | 32 (49.2%) | 67 (59.8%) | 39 (59.1%) | 209 (55.4%) |
| Mean (SD) | 9.59 (3.34) | 9.00 (4.06) | 9.61 (3.34) | 9.28 (3.18) | 9.45 (3.41) |
| Median (IQR) | 10.00 (8.00, 12.00) | 10.00(5.00, 12.00) | 10.00 (8.00, 12.00) | 10.00 (7.00, 12.00) | 10.00(8.00, 12.00) |
| Range | 1.00, 14.00 | 2.00, 15.00 | 1.00, 15.00 | 1.00, 14.00 | 1.00, 15.00 |
| (Missing) | 63 | 33 | 45 | 27 | 168 |
| **Follow-up OTSPSFC score** | | | | | |
| N | 50 (37.3%) | 31 (47.7%) | 39 (34.8%) | 33 (50.0%) | 153 (40.6%) |
| Mean (SD) | 10.14 (2.88) | 8.52 (4.10) | 9.23 (2.68) | 8.58 (3.67) | 9.24 (3.33) |
| Median (IQR) | 11.00(9.00, 12.00) | 10.00(5.00, 12.00) | 9.00(7.00, 11.00) | 9.00(6.00, 11.00) | 10.00(7.00, 12.00) |
| Range | 3.00, 15.00 | 2.00, 14.00 | 4.00, 14.00 | 1.00, 15.00 | 1.00, 15.00 |
| (Missing) | 84 | 34 | 73 | 33 | 224 |

Spatial Working Memory Between Search Errors (SWMBE) is the number of times the participant incorrectly revisits a box in which a token has previously been found. Calculated across all assessed four, six and eight token trials. Lower scores can be interpreted as better.

**Table 12s - Spatial Working Memory (SWMBE). The number of errors across the whole.**

| Variable | Group,  N = 134 | Independent, N = 65 | One-to-One, N = 112 | TAU,  N = 66 | Overall,  N = 377 |
| --- | --- | --- | --- | --- | --- |
| **Baseline SWMBE score** | | | | | |
| N | 128 (95.5%) | 62 (95.4%) | 102 (91.1%) | 61 (92.4%) | 353 (93.6%) |
| Mean (SD) | 14.37 (9.19) | 14.90 (10.18) | 13.98 (9.97) | 13.87 (9.52) | 14.26 (9.62) |
| Median (IQR) | 15.00(7.00, 22.00) | 17.00 (6.00, 23.00) | 15.50 (4.25, 21.75) | 16.00 (5.00, 21.00) | 16.00 (5.00, 22.00) |
| Range | 0.00, 32.00 | 0.00, 33.00 | 0.00, 33.00 | 0.00, 32.00 | 0.00, 33.00 |
| (Missing) | 6 | 3 | 10 | 5 | 24 |
| **Post-therapy SWMBE score** | | | | | |
| N | 68 (50.7%) | 31 (47.7%) | 66 (58.9%) | 38 (57.6%) | 203 (53.8%) |
| Mean (SD) | 11.90 (9.69) | 11.55 (10.21) | 11.29 (10.88) | 13.03 (9.35) | 11.86 (10.06) |
| Median (IQR) | 12.00(0.75, 20.25) | 9.00(0.50, 21.00) | 9.50(0.00, 20.75) | 14.00 (5.00, 20.00) | 12.00 (0.50, 21.00) |
| Range | 0.00, 28.00 | 0.00, 28.00 | 0.00, 33.00 | 0.00, 33.00 | 0.00, 33.00 |
| (Missing) | 66 | 34 | 46 | 28 | 174 |
| **Follow-up SWMBE score** | | | | | |
| N | 50 (37.3%) | 29 (44.6%) | 40 (35.7%) | 33 (50.0%) | 152 (40.3%) |
| Mean (SD) | 10.06 (9.17) | 12.21 (11.02) | 9.72 (10.01) | 12.06 (10.14) | 10.82 (9.93) |
| Median (IQR) | 9.50 (0.00, 18.50) | 15.00 (0.00, 24.00) | 5.50 (0.00, 19.00) | 10.00 (2.00, 22.00) | 9.50 (0.00, 20.00) |
| Range | 0.00, 28.00 | 0.00, 29.00 | 0.00, 30.00 | 0.00, 26.00 | 0.00, 30.00 |
| (Missing) | 84 | 36 | 72 | 33 | 225 |

Paired Associate Learning Total Errors (Adjusted) (PALTEA) is the number of times the participant chose the incorrect box for a stimulus on assessment problems, plus an adjustment for the estimated number of errors they would have made on any problems, attempts, and recalls they did not reach. This measure allows performance to be compared on errors made across all participants regardless of those who terminated early versus those completing the final stage of the task. Lower scores can be interpreted as better.

**Table 13s - Paired Associate Learning Total Errors (Adjusted) (PALTEA)**

| Variable | Group, N = 134 | Independent, N = 65 | One-to-One, N = 112 | TAU, N = 66 | Overall, N = 377 |
| --- | --- | --- | --- | --- | --- |
| **Baseline PALTEA score** | | | | | |
| N | 128 (93.3%) | 65 (100.0%) | 106 (94.6%) | 62 (93.9%) | 361 (95.8%) |
| Mean (SD) | 21.17 (16.45) | 21.51 (17.17) | 17.70 (16.16) | 19.73 (14.67) | 19.96 (16.21) |
| Median (IQR) | 16.00 (8.75, 30.00) | 18.00 (8.00, 33.00) | 12.00 (6.25, 24.00) | 16.00 (8.00, 24.75) | 15.00 (8.00, 28.00) |
| Range | 0.00, 67.00 | 0.00, 62.00 | 0.00, 63.00 | 2.00, 61.00 | 0.00, 67.00 |
| (Missing) | 6 | 0 | 6 | 4 | 16 |
| **Post-therapy PALTEA score** | | | | | |
| N | 69 (51.5%) | 31 (47.7%) | 64 (57.1%) | 37 (56.1%) | 201 (53.3%) |
| Mean (SD) | 16.84 (16.43) | 15.35 (15.90) | 16.88 (16.09) | 18.70 (15.45) | 16.97 (15.97) |
| Median (IQR) | 11.00 (4.00, 25.00) | 9.00 (5.00, 20.50) | 11.00 (5.00, 23.00) | 14.00 (8.00, 23.00) | 11.00 (5.00, 23.00) |
| Range | 0.00, 65.00 | 0.00, 58.00 | 0.00, 59.00 | 2.00, 63.00 | 0.00, 65.00 |
| (Missing) | 65 | 34 | 48 | 29 | 176 |
| **Follow-up PALTEA score** | | | | | |
| N | 50 (37.3%) | 30 (46.2%) | 40 (35.7%) | 33 (50.0%) | 153 (40.6%) |
| Mean (SD) | 13.84 (13.45) | 17.13 (15.43) | 15.28 (14.80) | 22.12 (16.49) | 16.65 (15.06) |
| Median (IQR) | 9.00 (3.25, 21.75) | 14.00 (3.75, 22.25) | 12.00 (4.75, 21.00) | 16.00 (11.00, 25.00) | 12.00 (5.00, 22.00) |
| Range | 0.00, 48.00 | 0.00, 50.00 | 0.00, 69.00 | 2.00, 60.00 | 0.00, 69.00 |
| (Missing) | 84 | 35 | 72 | 33 | 224 |

Rey Auditory Verbal Learning Test (RAVLT) is the sum of all attempts. Higher scores can be interpreted as better.

**Table 14s - Rey Auditory Verbal Learning Test (RAVLT)**

| Variable | Group, N = 134 | Independent, N = 65 | One-to-One,  N = 112 | TAU,  N = 66 | Overall,  N = 377 |
| --- | --- | --- | --- | --- | --- |
| **Baseline RAVLT score** | | | | | |
| N | 133 (99.3%) | 64 (98.5%) | 109 (97.3%) | 65 (98.5%) | 371 (98.4%) |
| Mean (SD) | 42.93 (11.19) | 42.62 (12.18) | 44.15 (11.10) | 43.35 (11.24) | 43.31 (11.32) |
| Median (IQR) | 42.00 (36.00, 51.00) | 42.00 (33.00, 52.00) | 45.00 (38.00, 53.00) | 42.00 (38.00, 49.00) | 43.00(36.00, 52.00) |
| Range | 10.00, 67.00 | 17.00, 66.00 | 17.00, 64.00 | 15.00, 73.00 | 10.00, 73.00 |
| (Missing) | 1 | 1 | 3 | 1 | 6 |
| **Post-therapy RAVLT score** | | | | | |
| N | 86 (64.2%) | 32 (49.2%) | 77 (68.8%) | 39 (59.1%) | 234 (62.1%) |
| Mean (SD) | 44.01 (12.83) | 46.91 (12.71) | 46.55 (12.01) | 44.33 (10.06) | 45.29 (12.11) |
| Median (IQR) | 44.50 (34.25, 52.75) | 50.00 (38.50, 56.25) | 48.00 (36.00, 54.00) | 43.00 (35.50, 52.00) | 46.00 (36.00, 54.00) |
| Range | 12.00, 69.00 | 18.00, 72.00 | 20.00, 72.00 | 28.00, 66.00 | 12.00, 72.00 |
| (Missing) | 48 | 33 | 35 | 27 | 143 |
| **Follow-up RAVLT score** | | | | | |
| N | 70 (52.2%) | 33 (50.8%) | 60 (52.6%) | 35 (53.0%) | 198 (52.5%) |
| Mean (SD) | 45.53 (11.77) | 47.64 (14.01) | 48.30 (12.72) | 45.29 (11.30) | 46.68 (12.36) |
| Median (IQR) | 43.50 (35.25, 54.00) | 50.00 (35.00, 60.00) | 50.00 (42.00, 56.00) | 46.00 (36.00, 53.00) | 47.00 (36.25, 55.75) |
| Range | 26.00, 68.00 | 17.00, 68.00 | 9.00, 75.00 | 25.00, 68.00 | 9.00, 75.00 |
| (Missing) | 64 | 32 | 52 | 31 | 179 |

**Other cognitive tests contributing to the composite score**

Table 15s shows the perseverative errors for the Computerised Wisconsin Card Sorting Task (WCST). Lower scores can be interpreted as better.

**Table 15s - Wisconsin Card Sorting Task (WCST) - perseverative errors**

| Variable | Group, N = 134 | Independent, N = 65 | One-to-One, N = 112 | TAU, N = 66 | Overall, N = 377 |
| --- | --- | --- | --- | --- | --- |
| **Baseline WCST score** | | | | | |
| N | 126 (94.0%) | 63 (96.9%) | 105 (93.8%) | 63 (95.5%) | 357 (94.7%) |
| Mean (SD) | 6.55 (4.32) | 7.56 (5.98) | 7.47 (5.01) | 7.43 (5.89) | 7.15 (5.13) |
| Median (IQR) | 6.00 (3.00, 9.00) | 6.00 (4.50, 9.00) | 7.00 (5.00, 10.00) | 7.00 (4.50, 9.00) | 6.00 (4.00, 9.00) |
| Range | 0.00, 19.00 | 0.00, 36.00 | 0.00, 30.00 | 0.00, 40.00 | 0.00, 40.00 |
| (Missing) | 8 | 2 | 7 | 3 | 20 |
| **Post-therapy WCST score** | | | | | |
| N | 65 (48.5%) | 28 (43.1%) | 65 (58.0%) | 35 (53.0%) | 193 (51.2%) |
| Mean (SD) | 7.02 (4.19) | 6.82 (6.97) | 6.20 (3.75) | 8.57 (11.79) | 6.99 (6.53) |
| Median (IQR) | 7.00 (4.00, 9.00) | 6.00 (4.00, 7.00) | 6.00 (4.00, 8.00) | 6.00 (5.50, 8.00) | 6.00 (4.00, 8.00) |
| Range | 0.00, 21.00 | 0.00, 37.00 | 0.00, 16.00 | 0.00, 72.00 | 0.00, 72.00 |
| (Missing) | 69 | 37 | 47 | 31 | 184 |
| **Follow-up WCST score** | | | | | |
| N | 57 (42.5%) | 26 (40.0%) | 46 (41.1%) | 28 (42.4%) | 157 (41.6%) |
| Mean (SD) | 7.61 (11.21) | 10.04 (14.13) | 7.67 (9.82) | 5.50 (3.49) | 7.66 (10.43) |
| Median (IQR) | 6.00 (5.00, 8.00) | 6.00 (5.00, 9.75) | 6.00 (5.00, 7.00) | 6.00 (2.75, 7.00) | 6.00 (5.00, 8.00) |
| Range | 0.00, 88.00 | 1.00, 73.00 | 0.00, 66.00 | 0.00, 14.00 | 0.00, 88.00 |
| (Missing) | 77 | 39 | 66 | 38 | 220 |

The Digit Span Task from the Wechsler Adult Intelligence Scale (WAIS) is a measure of verbal working memory. This is the sum of the backward raw score for the verbal working memory. Higher scores can be interpreted as better.

**Table 16s - Digit span task from WAIS**

| *Variable* | *Group, N = 134* | *Independent, N = 65* | *One-to-One, N = 112* | *TAU, N = 66* | *Overall, N = 377* |
| --- | --- | --- | --- | --- | --- |
| ***Baseline WAIS score*** | | | | | |
| *N* | *134 (100.0%)* | *65 (100.0%)* | *112 (100.0%)* | *66 (100.0%)* | *377 (100.0%)* |
| *Mean (SD)* | *15.49 (3.73)* | *15.32 (4.23)* | *15.83 (3.74)* | *15.05 (4.16)* | *15.48 (3.89)* |
| *Median (IQR)* | *15.00 (13.00, 18.00)* | *15.00 (12.00, 18.00)* | *15.50 (13.00, 18.00)* | *15.00 (12.00, 18.00)* | *15.00 (13.00, 18.00)* |
| *Range* | *8.00, 25.00* | *9.00, 28.00* | *8.00, 27.00* | *6.00, 30.00* | *6.00, 30.00* |
| *(Missing)* | *0* | *0* | *0* | *0* | *0* |
| ***Post-therapy WAIS score*** | | | | | |
| *N* | *87 (64.9%)* | *32 (49.2%)* | *77 (68.8%)* | *40 (60.6%)* | *236 (62.6%)* |
| *Mean (SD)* | *15.83 (4.19)* | *15.84 (4.22)* | *16.74 (3.93)* | *15.75 (4.00)* | *16.11 (4.08)* |
| *Median (IQR)* | *15.00 (12.00, 18.50)* | *15.00 (13.00, 20.00)* | *16.00 (15.00, 19.00)* | *15.00 (13.00, 18.25)* | *15.00 (13.00, 19.00)* |
| *Range* | *9.00, 28.00* | *9.00, 25.00* | *10.00, 28.00* | *8.00, 28.00* | *8.00, 28.00* |
| *(Missing)* | *47* | *33* | *35* | *26* | *141* |
| ***Follow-up WAIS score*** | | | | | |
| *N* | *70 (52.2%)* | *33 (50.8%)* | *60 (53.6%)* | *36 (54.5%)* | *199 (52.8%)* |
| *Mean (SD)* | *15.57 (4.01)* | *16.00 (4.47)* | *17.53 (4.32)* | *15.69 (4.35)* | *16.26 (4.30)* |
| *Median (IQR)* | *14.00 (13.00, 18.75)* | *15.00 (13.00, 18.00)* | *17.00 (15.00, 20.00)* | *15.50 (13.75, 18.00)* | *16.00 (13.00, 19.00)* |
| *Range* | *9.00, 25.00* | *9.00, 25.00* | *10.00, 27.00* | *5.00, 29.00* | *5.00, 29.00* |
| *(Missing)* | *64* | *32* | *52* | *30* | *178* |

**Table 17s - Baseline characteristics of those with and without a composite cognitive score at post-treatment or follow-up**

|  | **Post-treatment** | | | **Follow-up** | | |
| --- | --- | --- | --- | --- | --- | --- |
|  | **Composite score** | **No composite score** | **Overall** | **Composite score** | **No composite score** | **Overall** |
| **Age Mean (SD)** | 26.6 (5.6) | 25.6 (6.5) | 26.1 (6.1) | 26.4 (6.1) | 25.9 (6.0) | 26.1 (6.1) |
| **Ethnicity N (%)** |  |  |  |  |  |  |
| 1. White | 92 (46.9) | 93 (51.7) | 185 (49.2) | 78 (55.3) | 107 (45.5) | 185 (49.2) |
| 2. Black (African, Caribbean) | 54 (27.6) | 51 (28.3) | 105 (27.9) | 31 (22.0) | 74 (31.5) | 105 (27.9) |
| 3. Asian (ie. Bangladeshi, Indian, Pakistani) | 27 (13.8) | 16 (8.9) | 43 (11.4) | 14 (9.9) | 29 (12.3) | 43 (11.4) |
| 4. Other (Other - mixed; Other - mixed White and Black Caribbean) | 23 (11.7) | 20 (11.1) | 43 (11.4) | 18 (12.8) | 25 (10.6) | 43 (11.4) |
| **Gender N (%)** |  |  |  |  |  |  |
| 1. Male | 152 (77.2) | 123 (68.3) | 275 (72.9) | 99 (69.7) | 176 (74.9) | 275 (72.9) |
| 2. Female | 45 (22.8) | 57 (31.7) | 102 (27.1) | 43 (30.3) | 59 (25.1) | 102 (27.1) |
| **Pretherapy current employment status?** **N (%)** |  |  |  |  |  |  |
| 1. Unemployed | 134 (68.0) | 116 (64.4) | 250 (66.3) | 100 (70.4) | 150 (63.8) | 250 (66.3) |
| 2. Primary childcare giver | 2 (1.0) | 2 (1.1) | 4 (1.1) | 2 (1.4) | 2 (0.9) | 4 (1.1) |
| 3. In full-time education | 24 (12.2) | 28 (15.6) | 52 (13.8) | 19 (13.4) | 33 (14.0) | 52 (13.8) |
| 4. Part-time employed | 21 (10.7) | 15 (8.3) | 36 (9.5) | 14 (9.9) | 22 (9.4) | 36 (9.5) |
| 5. Full-time employed | 16 (8.1) | 19 (10.6) | 35 (9.3) | 7 (4.9) | 28 (11.9) | 35 (9.3) |
| **Pretherapy currently living?** **N (%)** |  |  |  |  |  |  |
| 1. Own property (private, rented) | 53 (27.0) | 63 (35.0) | 116 (30.9) | 41 (28.9) | 75 (32.1) | 116 (30.9) |
| 2. Parental home | 117 (59.7) | 88 (48.9) | 205 (54.5) | 84 (59.2) | 121 (51.7) | 205 (54.5) |
| 3. Temporary accommodation (e.g., Bed and breakfast) | 8 (4.1) | 7 (3.9) | 15 (4.0) | 4 (2.8) | 11 (4.7) | 15 (4.0) |
| 4. Supervised Group Home | 10 (5.1) | 11 (6.1) | 21 (5.6) | 7 (4.9) | 14 (6.0) | 21 (5.6) |
| 5. Supervised Hostel | 8 (4.1) | 11 (6.1) | 19 (5.1) | 6 (4.2) | 13 (5.6) | 19 (5.1) |
| **Pretherapy relationship status?** **N (%)** |  |  |  |  |  |  |
| 1. Single | 175 (88.8) | 156 (87.2) | 331 (88.0) | 125 (88.0) | 206 (88.0) | 331 (88.0) |
| 2. Living with partner | 13 (6.6) | 15 (8.4) | 28 (7.4) | 12 (8.5) | 16 (6.8) | 28 (7.4) |
| 3. Married / In a same-sex civil partnership | 7 (3.6) | 5 (2.8) | 12 (3.2) | 4 (2.8) | 8 (3.4) | 12 (3.2) |
| 4. Separated / Divorced | 2 (1.0) | 3 (1.7) | 5 (1.3) | 1 (0.7) | 4 (1.7) | 5 (1.3) |
| **Duration of untreated psychosis in months** **Mean (SD)** | 6.8 (13.8) | 10.7 (22.6) | 8.6 (18.5) | 5.9 (11.9) | 10.5 (21.7) | 8.6 (18.5) |

**Post hoc analyses of Group vs TAU and One-to-One vs TAU contrasts**

For the GAS primary outcome the Group vs TAU contrast found a medium statistically significant mean difference (Cohen’s d: 0.61, 0.20 – 1.01 95% CI, p=0.003) in favour of CR at post-therapy. The One-to-One vs TAU contrast also found a medium statistically significant mean difference (Cohen’s d: 0.53, 0.11 – 0.96 95% CI, p=0.014) in favour of CR at post-therapy. There was no evidence of a mean difference for either of these contrasts at 6-month (post-therapy) follow-up.

**Table 18s: Primary outcome (GAS) post-hoc results**

|  | **post-therapy** | | | | **6 month (post-therapy) follow up** | | | | |
| --- | --- | --- | --- | --- | --- | --- | --- | --- | --- |
| **Contrast** | **Estimated Mean Difference** | **P-value** | **Lower 95% CI** | **Upper 95% CI** | **Estimated Mean Difference** | **P-value** | **Lower 95% CI** | **Upper 95% CI** |  |
| Group vs TAU | **6.070** | **0.003** | **1.998** | **10.142** | 3.564 | 0.157 | -1.370 | 8.500 |  |
| One-to-One vs TAU | **5.333** | **0.014** | **1.066** | **9.599** | 1.589 | 0.546 | -3.574 | 6.753 |  |
| Group + One-to-One vs Independent | **5.040** | **0.016** | **0.949** | **9.130** | 4.018 | 0.102 | -0.798 | 8.834 |  |

**9. Statistical output supplementary information**

*(1) Interim* analysis effects: We undertook simulations to examine the bias in the treatment effect estimates arising from the interim analysis decision to close the two less promising of the four trial arms. Matching the intended study design and the actual study participant attrition as closely as possible we simulated the trial recruitment, attrition, and baseline, endpoint, and follow-up measures.  We then compared estimates according to the analysis method of the SAP that were obtained from analyses that (i) used the data available with all four trial-arms open to recruitment till the end of the study and (ii) used the data available from the four-arms up to the date of the interim analysis, after which data from subsequent recruitment to the two arms with the least promising treatment effect estimates was removed. Two simulations were undertaken; a null scenario where all treatment effects were zero and a second scenario where the treatment effects corresponded to those from the SAP specified primary analysis, unadjusted for the interim recruitment closures. All were sufficiently small to allow us to present the results from the unadjusted analyses of the pre-specified SAP.

*(2)* ***Potential clustering within site:*** The primary analysis (GAS) was repeated but including a random effect for site (as we might expect a group effect within the Group CR arm; however, group membership was not recorded in the database), with results below (Table 6s). This gives much greater precision but with a consistent interpretation.

*(3****) Sensitivity to compliance to visit windows:*** A sensitivity analysis was also carried out on the primary outcome using only data from follow-up visits (post-therapy and 6M post therapy) that occurred within the protocol specified visit windows.

*(4)* ***Missing at random assumption*:** Two different approaches were used; first we examined whether key demographic variables predicted primary outcome missingness. Secondly, we used last observation carried forward for the 6-month (post-therapy) visit carrying forward observations from the post-therapy visit for missing observations, using a linear regression model for GAS T-score with the same covariates as above.

*(5)* ***Potential effects of departures from missing at random***: In the main primary analysis, we have assumed that the missing data is missing at random; chiefly that missingness of values of GAS only depends on data collected/variables included in the model. For this sensitivity analysis, we used the rctmiss package in Stata 15 to carry out the primary analysis under a range of assumptions about the missing data, where the data and missingness are modelled jointly using a pattern-mixture model. Regression models with outcomes modelled separately were used instead of the *gsem* command (in order for rctmiss to work), with the same covariates included. The scenarios show the estimated treatment effect (with confidence intervals) for different values of delta, where delta is defined as the difference in the mean of the unobserved values of the outcome from the mean of the adjusted observed values. We chose values of delta from -12 to +0 on the GAS weighted T-score, based on 12 being approximately 1 standard deviation in GAS weighted T-score, and the expectation that GAS scores most likely to be lower in unobserved measurements (than higher). Scenarios are presented for each of the three contrasts.

**Results for sensitivity analyses**

(1) ***Interim effects:*** Table 6s uses simulations and simulated data to give a bound for potential bias in the results following the dropping of the Independent and TAU arms, under a global null hypothesis (assumption of no difference between any arms) and under a global alternative hypothesis (using values from the naïve analysis). The results show that there was little to no bias introduced in either scenario, and so we can be reasonably certain that the dropping of the independent and TAU arms does not bias the primary results.

| Table 19s Simulation results for sensitivity to interim analyses from 1000 simulations | | | | | | | | | |
| --- | --- | --- | --- | --- | --- | --- | --- | --- | --- |
|  | | **Global Null Hypothesis** | | | | **Global Alternative Hypothesis** | | | |
| Contrast | **week** | **Mean**  **Bias** | **SD of Bias** | **Min**  **Bias** | **Max**  **Bias** | **Mean**  **Bias** | **SD of Bias** | **Min**  **Bias** | **Max**  **Bias** |
| Group vs One-to-One | 15 | 0·00085 | 0·03169 | -0·11013 | 0·10703 | -0·00139 | 0·03203 | -0·11358 | 0·12447 |
|  | 39 | -0·00140 | 0·03627 | -0·11287 | 0·12034 | -0·00100 | 0·03664 | -0·12119 | 0·11838 |
| Independent vs TAU | 15 | 0·01030 | 0·66916 | -1·78488 | 2·00931 | -0·03510 | 0·66885 | -2·18093 | 2·15679 |
|  | 39 | -0·01249 | 0·73514 | -2·16923 | 2·31994 | -0·00831 | 0·73717 | -2·13506 | 2·25477 |
| Group + One-to-One vs TAU | 15 | -0·01210 | 0·45974 | -1·67115 | 1·28465 | -0·00693 | 0·47356 | -1·49734 | 1·57357 |
|  | 39 | -0·01388 | 0·51898 | -1·46914 | 1·48719 | 0·01852 | 0·50866 | -1·94850 | 1·53276 |

*(2)* ***Potential clustering within site***

| Table 20s Potential clustering within site | | | | | | | | |
| --- | --- | --- | --- | --- | --- | --- | --- | --- |
|  | **Post-therapy** | | | | **6M Follow-up** | | | |
| Contrast | **Mean Difference** | **SE** | **P-value** | **95% CI** | **Mean Difference** | **SE** | **P-value** | **95% CI** |
| Group vs One-to-One | 0·737 | 1·400 | 0·598 | -2·006, 3·481 | 1·975 | 2·134 | 0·355 | -2·208, 6·158 |
| Independent vs TAU | 0·695 | 1·960 | 0·723 | -3·147, 4·536 | -1·353 | 1·305 | 0·300 | -3·911, 1·206 |
| Group + One-to-One vs TAU | 5·734 | 1·757 | 0·001 | 2·291, 9·177 | 2·665 | 1·090 | 0·014 | 0·530, 4·801 |

*(3****) Sensitivity to compliance to visit windows****:* Results below (Table 21s) are broadly similar, especially for Group + One-to-One vs TAU at post-therapy although this difference no longer statistically significant at a p<0.05 threshold, although likely due to lower power with less participants. There is a considerably higher point estimate for the Group + One-to-One vs TAU difference at 6-month post-therapy FU (although not statistically significant), suggesting the variation in time for this visit may be affecting the primary analysis results at this timepoint, although not so much as to have changed the overall conclusion.

| Table 21s Sensitivity to compliance to visit windows | | | | | | | | |
| --- | --- | --- | --- | --- | --- | --- | --- | --- |
|  | **Post-therapy** | | | | **6M Follow-up** | | | |
| Contrast | **Mean Difference** | **SE** | **P-value** | **95% CI** | **Mean Difference** | **SE** | **P-value** | **95% CI** |
| Group vs One-to-One | 0·486 | 2·196 | 0·825 | -3·852, 4·824 | 2·180 | 3·389 | 0·522 | -4·566, 8·926 |
| Independent vs TAU | -0·706 | 3·320 | 0·832 | -7·266, 5·854 | 2·522 | 5·037 | 0·618 | -7·504, 12·548 |
| Group + One-to-One vs TAU | 5·074 | 2·599 | 0·053 | -0·061, 10·208 | 6·741 | 4·423 | 0·131 | -2·062, 15·544 |

*(4)* ***Missing at random assumption:*** (Table 8s) Demographic predictors of missing primary outcome data were investigated: ethnicity, gender, employment, living situation and relationship status. None of these were found to be predictive of outcome missingness.

An analysis of the 6-month post-therapy follow-up visit for the primary outcome using last observation carried forward for missing observations (where not missing at post-therapy) gives similar results as the main analysis. There is a greater difference in the Group+ One-to-One vs TAU comparison though, although this may just be a consequence of the difference found at post-therapy being carried forward for the missing observations.

| Table 22s Missing at random assumption | | | | | |
| --- | --- | --- | --- | --- | --- |
| Contrast | **Mean Difference** | **SE** | **P-value** | **Lower 95% CI** | **Upper 95% CI** |
| Group vs One-to-One | 2·448 | 1·749 | 0·162 | -0·980 | 5·876 |
| Independent vs TAU | -0·428 | 2·529 | 0·866 | -5·385 | 4·528 |
| Group + One-to-One vs TAU | 3·883 | 2·053 | 0·059 | -0·140 | 7·906 |

*(5)* ***Potential effects of departures from missing at random:***

Scenario 1 (Group (1) vs One-to-One (0)) shows that even with a large mean difference in the unobserved observations by arm, we still would not have seen strong evidence of a difference between arms at post-therapy (as confidence intervals do not cross zero). The figure at 6 months post-therapy shows that a large difference in unobserved values in favour of the Group arm (with mean of the unobserved values in the One-to-One arm >8 than in the Group arm) would give evidence of a difference in arms in GAS T-score at 6M follow-up, but such a large difference is highly implausible.

Scenario 2 (Independent (1) versus TAU (0)) show that even with a very large delta of 12 (which is highly implausible) in favour of the Independent arm, the treatment effect for Independent vs TAU would still not be statistically significant (at p<0.05)

Scenario 3 (Group + One-to-One (1) versus TAU (0)) If the mean of the unobserved values in Group and One-to-One were lower than the unobserved values in TAU, then evidence of the treatment effect tends to be reduced and may be not statistically significant (using p<0.05 threshold) even for small values of delta. This is true for both visits. This should be considered a limitation of the analysis.

**Figure 1s: Sensitivity to non-ignorable missing data (missing not-at-random)**
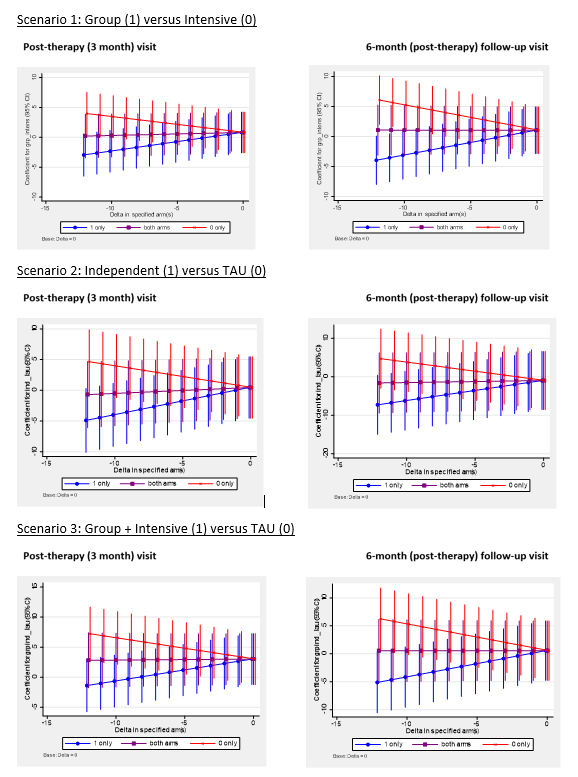


**10. Health economic supplementary data**

**Table** 23s - all service use data for each trial arm: Baseline Resource Use: Inpatient hospital services and outpatient hospital services

| **Inpatient hospital services** | **Group CR (n=134)** | | | | **Independent CR (n=65)** | | | | **One-to-One CR (n=112)** | | | | **Treatment as Usual (n=66)** | | | |
| --- | --- | --- | --- | --- | --- | --- | --- | --- | --- | --- | --- | --- | --- | --- | --- | --- |
|  | **N (%) using** | **Mean**  **Days/**  **Contacts^1^** | **Mean Costs (SD)^2^** | **Mean Costs of total sample (SD)^3^** | **N (%) using** | **Mean**  **Days/**  **Contacts^1^** | **Mean Costs (SD)^2^** | **Mean Costs of total sample (SD)^3^** | **N (%) using** | **Mean**  **Days/**  **Contacts^1^** | **Mean Costs (SD)^2^** | **Mean Costs of total sample (SD)^3^** | **N (%) using** | **Mean**  **Days/**  **Contacts^1^** | **Mean Costs (SD)^2^** | **Mean Costs of total sample (SD)^3^** |
| Acute psychiatric ward | 23  (17) | 35·0  (30·2) | 14,822  (12,812) | 2,563  (7,682) | 8  (12) | 48·6  (44·4) | 20,591  (18,815) | 2,615  (9,36) | 16  (14) | 51·4  (42·2) | 21,810  (17,898) | 3,201  (10,228) | 8  (12) | 37·5  (25·7) | 15,900 (10,909) | 2,019  (6,474) |
| Psychiatric Rehabilitation ward | 1  (1) | 30·0  (0) | 12,720  (-) | 96  (1,103) | 0  (0) | - | - | 0  (0) | 2  (2) | 14·5  (19·1) | 6,148 (8,095) | 113  (1,137) | 0  (0) | - | 0  (0) | - |
| Long-stay ward | 2  (1) | 61·0  (53·7) | 25,864  (22,786) | 389  (3,730) | 0  (0) | - | - | 0  (0) | 1  (1) | 31·0  (-) | 13,144  (-) | 121  (1,259) | 0  (0) | - | 0  (0) | - |
| Emergency/Crisis centre | 4  (3) | 85·0  (80·9) | 36,051  (34,296) | 1,084  (8,058) | 0  (0) | - | - | 0  (0) | 0  (0) | - | - | - | 0  (0) | - | 0  (0) | - |
| General medical ward | 2  (1) | 2·5  (2·1) | 1,505  (1,277) | 23  (2145) | 0  (0) | - | - | 0  (0) | 5  (4) | 2·4  (1·3) | 1,445 (808) | 66  (341) | 2  (3) | 2·0  (1·4) | 1,204 (851) | 38  (239) |
| Other | 0  (0) | - | - | - | 0  (0) | - | - | 0  (0) | 1  (1) | 92·0  (-) | 39,008  (-) | 358  (3,736) | 0  (0) | - | - | - |
| **Total inpatient costs across all services^4^** | 32 | 40·7  (42·2) | 17,267  (17,848) | 4,155  (11,389) | 8 | 48·6  (44·4) | 20,591  (18,815) | 2,615  (9,366) | 25 | 39·5  (41·0) | 16,825  (17,305) | 3,859  (10,819) | 10 | 30·4  (27·2) | 12,961  (11,447) | 2,057  (6,466) |
| **Outpatient hospital services** |  |  |  |  |  |  |  |  |  |  |  |  |  |  |  |  |
| Psychiatric outpatient visit | 8  (6) | 4·4  (4·4) | 591  (599·) | 36  (197) | 6  (9) | 19·7  (41·9) | 2,655  (5,650) | 261  (1,816) | 8  (7) | 3·2  (2·1) | 430  (289) | 32  (135) | 4  (6) | 3·8  (5·5) | 506  (743) | 31  (202) |
| Other hospital outpatient visit (including A&E) | 28  (21) | 1·9  (2·5) | 251  (344) | 53  (186) | 10  (15) | 4·2  (7·8) | 567  (1,047) | 93  (458) | 24  (21) | 1·3  (0·7) | 180  (95) | 40  (87) | 11  (17) | 2·3  (2·8) | 307  (373) | 52  (187) |
| Day hospital | 3  (2) | 9·0  (8·5) | 3,618  (3435) | 82  (685) | 2  (3) | 48·5  (16·3) | 19,497  6,538 | 639  (3,601) | 2  (2) | 1·0  (0·0) | 402  (0) | 7  (54) | 2  (3) | 1·5  (0·7) | 603  (284) | 19  (111) |
| Clozapine clinic | 2  (1) | 6·0  (0·0) | 810  (0) | 12  (99) | 2  (3) | 12·0  (0·0) | 1,620  (0) | 53  (291) | 2  (2) | 11·0  (7·1) | 1,485  (955) | 28  (221) | 1  (2) | 1·0  (-) | 135  (-) | 2  (17) |
| Other | 5  (4) | 4·8  (5·0) | 648  (671) | 24  (170) | 3  (5) | 2·0  (1·7) | 270  (234) | 13  (73) | 1  (1) | 1·0  (-) | 135  (-) | 1  (13) | 2  (3) | 13·0  (15·6) | 1,755  (2,100) | 54  (403) |
| **Total Outpatient costs across all services^4^** | 42 | 3·6  (4·5) | 654  (1,244) | 206  (757) | 22 | 13·0  (26·2) | 2,938  (6,373) | 1,060  (4,030) | 34 | 2·4  (3·3) | 343  (438) | 108  (291) | 18 | 3·9  (6·5) | 570  (912) | 158  (536) |

^1 Mean number of days/ contacts for those who had at least one episode/ contact.^

^2 Mean costs for those who had at least one episode/contact.^

^3 Mean costs for the whole sample – (if not missing; code 888 or 999) (Inpatient admissions; Group CR (n=133), Independent CR (n=63), One-to-One CR (n=109), Treatment as Usual (n=63)); (Outpatient contacts; (Group CR (n=133), Independent CR (n=61), One-to-One CR (n=108), Treatment as Usual (n=65)).^

^4 Mean total costs for those that used a service across all services.^ **Baseline Resource Use: Community-based day services and primary and care contacts**

| **Community-based day services** | **Group CR (n=134)** | | | | **Independent CR (n=65)** | | | |
| --- | --- | --- | --- | --- | --- | --- | --- | --- |
|  | **N (%) using** | **Mean**  **Days/**  **Contacts^1^** | **Mean Costs (SD)^3^** | **Mean Costs of total sample (SD)^4^** | **N (%) using** | **Mean**  **Days/**  **Contacts^1^** | **Mean Costs (SD)^3^** | **Mean Costs of total sample (SD)^4^** |
| Community mental health centre | 17  (13) | 5·1  (3·2) | 197  (241) | 25  (107) | 17  (26) | 10·5  (8·9) | 488  (443) | 130  (311) |
| Day care centre | 1  (1) | 14·0  (-) | 1,735  (-) | 13  (150) | 1  (2) | 24·0  (-) | 17  (-) | 0  (2) |
| Sheltered workshop | 0  (0) | - | - | 0  (0) | 0  (0) | - | - | 0  (0) |
| Specialist education / Recovery college | 9  (7) | 3·1  (2·3) | 343  (519) | 23  (154) | 3  (5) | 24·7  (26·1) | 876  (1,486) | 41  (324) |
| **Total Community-based day services costs across all services^5^** | 24 | 5·4  (3·8) | 340  (472) | 61  (237) | 20 | 13·9  (14·3) | 547  (648) | 171  (438) |
| **Primary and care contacts** |  |  |  |  |  |  |  |  |
| Psychiatrist | 97  (72) | 4·1  (7·0) | 342  (764) | 248  (667) | 44  (68) | 3·8  (2·6) | 417  (575) | 287  (514) |
| Psychologist | 51  (38) | 7·9  (13·9) | 433  (806) | 165  (537) | 26  (40) | 8·8  (8·7) | 526  (498) | 214  (408) |
| GP | 66  (49) | 2·7  2·4) | 175  (176) | 86  (151) | 34  (52) | 4·0  (4·3) | 276  (345) | 146  (285) |
| District nurse | 5  (4) | 22·6  (43·4) | 2,071  (4,324) | 77  (847) | 4  (6) | 1·3  (0·5) | 12  (3) | 1  (3) |
| Crisis team member | 10  (7) | 9·1  (9·9) | 173  (335) | 13  (98) | 7  (11) | 9·1  (10·9) | 113  (105) | 12  (48) |
| Community psychiatric nurse | 115  (86) | 11·3  (7·9) | 433  (483) | 372  (472) | 46  (71) | 13·6  (9·3) | 556  (650) | 400  (604) |
| Social Worker | 7  (5) | 10·0  (9·7) | 418  (571) | 22  (153) | 8  (12) | 11·6  (8·8) | 405  (431) | 51  (197) |
| Occupational therapist | 14  (10) | 7·5  (7·0) | 235  (249) | 25  (106) | 7  (11) | 13·6  (10·7) | 455  (403) | 50  (190) |
| Support worker | 24  (18) | 25·5  (26·9) | 813  (1,128) | 146  (564) | 22  (34) | 16·7  (10·1) | 525  (452) | 180  (362) |
| **Total Primary and care costs across all services^5^** | 133 | 19·3  (18·1) | 1,161  (1,714) | 1,153  (1,710) | 63 | 20·9  (14·5) | 1,362  (1,306) | 1,340  (1,306) |

^1 Mean number of days/ contacts for those who had at least one episode/ contact.^

^2 Mean duration of attendance/contacts for those who had at least one attendance/ contact.^

^3 Mean costs for those who had at least one attendance/contact.^

^4 Mean costs for the whole sample – (if not missing; code 888 or 999) (Community-based day services; Group CR (n=133), Independent CR (n=64), One-to-One CR (n=107), Treatment as Usual (n=64)); Primary and care contacts; (Group CR (n=134), Independent CR (n=64), One-to-One CR (n=110), Treatment as Usual (n=66)).^

^5 Mean total costs for those that used a service across all services.^

**Baseline Resource Use: Community-based day services and primary and care contacts (continued)**

| **Community-based day services** | **One-to-One CR (n=112)** | | | | **Treatment as Usual (n=66)** | | | |
| --- | --- | --- | --- | --- | --- | --- | --- | --- |
|  | **N (%) using** | **Mean**  **Days/**  **Contacts^1^** | **Mean Costs (SD)^3^** | **Mean Costs of total sample (SD)^4^** | **N (%) using** | **Mean**  **Days/**  **Contacts^1^** | **Mean Costs (SD)^3^** | **Mean Costs of total sample (SD)^4^** |
| Community mental health centre | 12  (11) | 12·5  (14·9) | 449  (592) | 50  (238) | 9  (14) | 10·0  (8·7) | 714  (1,100) | 100  (465) |
| Day care centre | 1  (1) | 1·0  (-) | 83  (-) | 1  (8) | 1  (2) | 3·0  (-) | 124  (-) | 2  (15) |
| Sheltered workshop | 0  (0) | - | - | 0  (0) | 0  (0) | - | - | 0  (0) |
| Specialist education / Recovery college | 7  (6) | 13·0  (16·5) | 1,505  (1,863) | 98  (580) | 4  (6) | 11·8  (12·6) | 853  (1,122) | 53  (321) |
| **Total Community-based day services costs across all services^5^** | 18 | 13·4  (15·8) | 889  (1,304) | 150  (620) | 12 | 11·7  (10·9) | 830  (1,105) | 156  (566) |
| **Primary and care contacts** |  |  |  |  |  |  |  |  |
| Psychiatrist | 86  (77) | 3·1  (2·7) | 218  (247) | 171  (236) | 53  (80) | 3·4  (3·2) | 317  (387) | 255  (369) |
| Psychologist | 35  (31) | 6·9  (6·2) | 405  (377) | 129  (283) | 24  (36) | 8·4  (7·5) | 446  (376) | 162  (311) |
| GP | 56  (50) | 2·8  (2·9) | 193  (237) | 98  (194) | 34  (52) | 4·1  (3·3) | 252  (230) | 130  (207) |
| District nurse | 2  (2) | 21·5  (29·0) | 345  (482) | 6  (65) | 2  (3) | 1·5  (0·7) | 14  (3) | 0  (2) |
| Crisis team member | 6  (5) | 13·0  (23·1) | 219  (428) | 12  (104) | 8  (12) | 6·3  (9·9) | 79  (89) | 10  (39) |
| Community psychiatric nurse | 87  (78) | 12·6  (7·7) | 432  (388) | 342  (387) | 57  (86) | 11·7  (8·7) | 530  (742) | 458  (713) |
| Social Worker | 8  (7) | 30·8  (61·6) | 379  (281) | 28  (122) | 3  (5) | 7·3  (5·0) | 334  (507) | 15  (113) |
| Occupational therapist | 8  (7) | 18·9  (18·4) | 438  (441) | 32  (160) | 6  (9) | 6·5  (4·8) | 178  (194) | 16  (74) |
| Support worker | 24  (21) | 13·8  (20·1) | 233  (204) | 51  (135) | 10  (15) | 10·8  (8·8) | 320  (321) | 49  (166) |
| **Total Primary and care contacts^5^** | 109 | 19·5  (21·0) | 876  (673) | 868  (675) | 66 | 19·1  (13·7) | 1,095  (975) | 1,095  (975) |

^1 Mean number of days/ contacts for those who had at least one episode/ contact.^

^2 Mean duration of attendance/contacts for those who had at least one attendance/ contact.^

^3 Mean costs for those who had at least one attendance/contact.^

^4 Mean costs for the whole sample – (if not missing; code 888 or 999) (Community-based day services; Group CR (n=133), Independent CR (n=64), One-to-One CR (n=107), Treatment as Usual (n=64)); Primary and care contacts; (Group CR (n=134), Independent CR (n=64), One-to-One CR (n=110), Treatment as Usual (n=66)).^

^5 Mean total costs for those that used a service across all services.^ **Baseline Resource Use: Criminal justice services**

|  | **Group CR (n=134)** | | | | **Independent CR (n=65)** | | | | **One-to-One CR (n=112)** | | | | | **Treatment as Usual (n=66)** | | | |
| --- | --- | --- | --- | --- | --- | --- | --- | --- | --- | --- | --- | --- | --- | --- | --- | --- | --- |
|  | **N (%) using** | **Mean**  **Days/**  **Contacts^1^** | **Mean Costs (SD)^2^** | **Mean Costs of total sample (SD)^3^** | **N (%) using** | **Mean**  **Days/**  **Contacts^1^** | **Mean Costs (SD)^2^** | **Mean Costs of total sample (SD)^3^** | **N (%) using** | **Mean**  **Days/**  **Contacts^1^** | **Mean Costs (SD)^2^** | **Mean Costs of total sample (SD)^3^** | **N (%) using** | | **Mean**  **Days/**  **Contacts^1^** | **Mean Costs (SD)^2^** | **Mean Costs of total sample (SD)^3^** |
| **Over the last 6 months, has the patient been in contact with the criminal justice services?** | | | | | | | | | | | | | | | | | |
| How many contacts with the police?  (contact=interview or stay of some hours but not overnight) | 9  (7) | 1·7 (1·3) | 105  (83) | 7  (34) | 3  (5) | 1·3 (0·6) | 84  (36) | 3·94  (19·06) | 10  (9) | 1·3  (0·5) | 82  (30) | 7  (25) | 5*  (8) | | 2·2  (1·2) | 139  (82) | 11  (43) |
| How many nights spent in a police cell or prison? | 4  (3) | 22·8  (41·5) | 2,907  (3,609) | 87  (738) | 1  (2) | 1·0  (-) | 1,044  (-) | 16·32  (130·53) | 4  (4) | 1·8  (1·5) | 880  (329) | 32  (174) | 2  (3) | | 2·5  (2·1) | 715  (465) | 22  (138) |
| How many psychiatric assessments whilst in custody? | 4  (3) | 1·3  (0·5) | 326  (131) | 10  (59) | 0  (0) | - | - | 0  (0) | 7  (6) | 1·0  (0·0) | 261  (-) | 17  (64) | 4  (6) | | 2·0  (1·4) | 522  (369) | 32  (148) |
| How many criminal court appearances? | 2  (1) | 1·5  (0·7) | 65  (31) | 1  (8) | 0  (0) | - | - | 0  (0) | 1  (1) | 1·0  (-) | 44  (-) | 0  (4) | 1  (2) | | 1·0  (-) | 44  (-) | 1  (5) |
| How many civil court appearances? | 1  (1) | 3·0  (-) | 131  (-) | 1  (11) | 2  (3) | 1·0  (0) | 44  (0) | 1·36  (7·66) | 1  (1) | 1·0  (-) | 44  (-) | 0  (4) | 0  (0) | | - | - | 0  (0) |
| **Total** | 10 | - | 1,414  (2,685) | 106  (795) | 5 | - | 277  (466) | 21·62  (139·16) | 10 | - | 625  (559) | 57  (242) | 6 | | - | 709  (648) | 64  (273) |

^1 Mean number of days/ contacts for those who had at least one episode/ contact.^

^2 Mean costs for those who had at least one episode/contact.^

^3 Mean costs for the whole sample-(Group CR (n=133), Independent CR (n=64), One-to-One CR (n=110), Treatment as Usual (n=65)).^

^*1 patient missing data^**Post CR Resource Use: Inpatient hospital services and outpatient hospital services**

|  | **Group CR (n=110)** | | | | **Independent CR (n=50)** | | | | **One-to-One CR (n=96)** | | | | **Treatment as Usual (n=57)** | | | |
| --- | --- | --- | --- | --- | --- | --- | --- | --- | --- | --- | --- | --- | --- | --- | --- | --- |
|  | **N (%) using** | **Mean**  **Days/**  **Contacts^1^** | **Mean Costs (SD)^2^** | **Mean Costs of total sample (SD)^3^** | **N (%) using** | **Mean**  **Days/**  **Contacts^1^** | **Mean Costs (SD)^2^** | **Mean Costs of total sample (SD)^3^** | **N (%) using** | **Mean**  **Days/**  **Contacts^1^** | **Mean Costs (SD)^2^** | **Mean Costs of total sample (SD)^3^** | **N (%) using** | **Mean**  **Days/**  **Contacts^1^** | **Mean Costs (SD)^2^** | **Mean Costs of total sample (SD)^3^** |
| **Inpatient hospital services** |  |  |  |  |  |  |  |  |  |  |  |  |  |  |  |  |
| Acute psychiatric ward | 1  (1) | 10·0  (-) | 4,240  (-) | 44  (431) | 2  (4) | 30·0  (-) | 12,720  (0) | 592  (2,710) | 2  (2) | 31·0  (35·4) | 13,144  (14,991) | 289  (2,500) | 4  (7) | 41·3  (32·7) | 17,490  (13,871) | 1,345  (5,785) |
| Psychiatric Rehabilitation ward | 0  (0) | - | - | 0  (0) | 0  (0) | - | - | 0  (0) | 0  (0) | - | - | 0  (0) | 0  (0) | - | - | 0  (0) |
| Long-stay ward | 1  (1) | 15·0  (-) | 6,360  (-) | 66  (646) | 1  (2) | 17·0  (-) | 7,208  (-) | 168  (1,099) | 0  (0) | - | - | 0  (0) | 0  (0) | - | - | 0  (0) |
| Emergency/Crisis centre | 1  (1) | 15·0  (-) | 6,360  (-) | 66  (646) | 0  (0) | - | - | 0  (0) | 0  (0) | - | - | 0  (0) | 0  (0) | - | - | 0  (0) |
| General medical ward | 2  (2) | 9·5  (9·2) | 5,719  (5,534) | 118  (993) | 3  (6) | 7·0  (8·7) | 4,214  (5,248) | 294  (1,578) | 1  (1) | 2·0  (-) | 1,204  (-) | 13  (126) | 0  (0) | - | - | 0  (0) |
| Other | 1  (1) | 15·0  (-) | 6,360  (-) | 66  (646) | 0  (0) | - | - | 0  (0) | 0  (0) | - | - | 0  (0) | 0  (0) | - | - | 0  (0) |
| **Total inpatient costs across all services^4^** | 4 | 18·5  (10·0) | 8,690  (4,479) | 358  (1,909) | 5 | 19·6  (11·9) | 9,058  (4,993) | 1,053  (3,318) | 3 | 21·3  (30·1) | 9,164  (12,644) | 302  (2,502) | 4 | 41·3  (32·7) | 17,490  (13,871) | 1,345  (5,785) |
| **Outpatient hospital services** |  |  |  |  |  |  |  |  |  |  |  |  |  |  |  |  |
| Psychiatric outpatient visit | 2  (2) | 2·2  (1·4) | 270  (191) | 6  (43) | 0  (0) | - | - | 0  (0) | 1  (1) | 1·0  (-) | 135  (-) | 2  (14) | 3  (5) | 1·3  (0·6) | 180  (78) | 10  (45) |
| Other hospital outpatient visit (including A&E) | 11  (10) | 1·2  (0·6) | 160  (81) | 18  (57) | 5  (10) | 1·6  (0·5) | 216  (74) | 25  (73) | 9  (9) | 2·1  (1·7) | 285  (228) | 29  (110) | 7  (12) | 1·7  (1·1) | 231  (150) | 31  (95) |
| Day hospital | 1  (1) | 1·0  (-) | 402  (-) | 4  (41) | 0  (0) | - | - | 0  (0) | 1  (1) | 12·0  (-) | 4,824  (-) | 54  (508) | 1  (2) | 10·0  (-) | 4,020  (-) | 77  (557) |
| Clozapine clinic | 3  (3) | 3·7  (1·2) | 495  (156) | 15  (89) | 3  (6) | 10·3  (2·9) | 1,395  (390) | 95  (365) | 2  (2) | 6·5  (6·4) | 878  (859) | 20  (159) | 1  (2) | 2·0  (-) | 270  (-) | 5  (37) |
| Other | 3  (3) | 1·0  (0·0) | 135  (0) | 4  (23) | 1  (2) | 2·0  (-) | 270  (-) | 6  (41) | 2  (2) | 2·0  (1·4) | 270  (191) | 6  (45) | 0  (0) | - | - | 0  (0) |
| **Total Outpatient costs across all services^4^** | 19 | 1·7  (1·3) | 241  (23) | 47  (138) | 9 | 4·6  (4·6) | 615  (619) | 126  (366) | 13 | 3·8  (4·4) | 755  (1,398) | 109  (579) | 12 | 2·3  (2·6) | 538  (1,103) | 124  (561) |

^1 Mean number of days/ contacts for those who had at least one episode/ contact.^

^2 Mean costs for those who had at least one episode/contact.^

^3 Mean costs for the whole sample – (if not missing; code 888 or 999) (Inpatient admissions; Group CR (n=97), Independent CR (n=43), One-to-One CR (n=91), Treatment as Usual (n=52)); (Outpatient contacts; (Group CR (n=98), Independent CR (n=44), One-to-One CR (n=90), Treatment as Usual (n=52)).^

^4 Mean total costs for those that used a service across all services.^

**Post CR Resource Use: Community-based day services and primary and care contacts**

|  | **Group CR (n=110)** | | | | **Independent CR (n=50)** | | | |
| --- | --- | --- | --- | --- | --- | --- | --- | --- |
| **Community-based day services** | **N (%) using** | **Mean**  **Days/**  **Contacts^1^** | **Mean Costs (SD)^3^** | **Mean Costs of total sample (SD)^4^** | **N (%) using** | **Mean**  **Days/**  **Contacts^1^** | **Mean Costs (SD)^3^** | **Mean Costs of total sample (SD)^4^** |
| Community mental health centre | 4  (4) | 2·5  (1·7) | 86  (56) | 4  (20) | 2  (4) | 3·0  (0) | 186  (88) | 9  (42) |
| Day care centre | 0  (0) | - | - | 0  (0) | 0  (0) | - | - | 0  (0) |
| Sheltered workshop | 0  (0) | - | - | 0  (0) | 0  (0) | - | - | 0  (0) |
| Specialist education / Recovery college | 2  (2) | 1·5  (0·7) | 432  (204) | 9  (66) | 1  (2) | 16·0  (-) | 768  (-) | 18  (119) |
| **Total Community-based day services costs across all services^5^** | 6 | 2·2  (1·5) | 201  (205) | 13  (69) | 3 | 7·3  (7·5) | 380  (342) | 27  (125) |
| **Primary and care contacts^5^** |  |  |  |  |  |  |  |  |
| Psychiatrist | 66  (60) | 2·1  (1·7) | 168  (183) | 106  (167) | 31  (62) | 1·5  (0·9) | 97  (83) | 62  (81) |
| Psychologist | 31  (28) | 3·8  (3·8) | 186  (200) | 55  (138) | 7  (14) | 6·4  (3·9) | 373  (224) | 54  (155) |
| GP | 35  (32) | 1·8  (1·1) | 144  (169) | 48  (119) | 14  (28) | 1·6  (1·1) | 96  (63) | 28  (55) |
| District nurse | 2*  (2) |  | 27  (32) | 1  (5) | 0  (0) | - | - | 0  (0) |
| Crisis team member | 1  (1) | 1·0  (-) | 36  (-) | 0  (4) | 3  (6) | 2·0  (1·7) | 13  (10) | 1  (4) |
| Community psychiatric nurse | 86  (78) | 5·3  (4·2) | 257  (762) | 213  (700) | 36  (72) | 4·8  (4·8) | 193  (306) | 144  (277) |
| Social Worker | 1  (1) | 4·0  (-) | 51  (-) | 0  (5) | 4  (8) | 4·5  (5·1) | 204  (272) | 17  (89) |
| Occupational therapist | 1  (1) | 12·0  (-) | 216  (-) | 2  (21) | 0  (0) | - | - | 0  (0) |
| Support worker | 14  (13) | 5·3  (3·9) | 174  (219) | 23  (98) | 16  (32) | 5·6  (4·7) | 129  (147) | 43  (104) |
| **Total Primary and care contacts^5^** | 98 | 8·9  (6·1) | 477  (750) | 450  (737) | 47 | 8·5  (7·3) | 357  (371) | 350  (371) |

^1 Mean number of days/ contacts for those who had at least one episode/ contact.^

^2 Mean duration of attendance/contacts for those who had at least one attendance/ contact.^

^3 Mean costs for those who had at least one attendance/contact.^

^4 Mean costs for the whole sample – (if not missing; code 888 or 999) (Community-based day services; Group CR (n=94), Independent CR (n=42), One-to-One CR (n=90), Treatment as Usual (n=51)); Primary and care contacts; (Group CR (n=104), Independent CR (n=48), One-to-One CR (n=92), Treatment as Usual (n=54)).^

^5 Mean total costs for those that used a service across all services.^

**Post CR: Resource Use: Community-based day services and primary and care contacts (continued)**

| **Community-based day services** | **One-to-One CR (n=96)** | | | | **Treatment as Usual (n=57)** | | | |
| --- | --- | --- | --- | --- | --- | --- | --- | --- |
|  | **N (%) using** | **Mean**  **Days/**  **Contacts^1^** | **Mean Costs (SD)^3^** | **Mean Costs of total sample (SD)^4^** | **N (%) using** | **Mean**  **Days/**  **Contacts^1^** | **Mean Costs (SD)^3^** | **Mean Costs of total sample (SD)^4^** |
| Community mental health centre | 7  (7) | 4·3  (4·0) | 1,130  (2,582) | 88  (736) | 1  (2) | 3·0  (-) | - | - |
| Day care centre | 2  (2) | 21·0  (21·2) | 1,859  (526) | 41  (281) | 0  (0) | - | - | 0  (0) |
| Sheltered workshop | 2  (2) | 6·5  (7·8) | 888  (1,188) | 20  (182) | 0  (0) | - | - | 0  (0) |
| Specialist education / Recovery college | 4  (4) | 2·6  (2·3) | 276  (201) | 12  (68) | 0  (0) | - | - | 0  (0) |
| **Total Community-based day services costs across all services** | 13 | 7·3  (11·2) | 1,116  (1,967) | 161  (823) | 0  (0) | - | - | 0  (0) |
| **Primary and care contacts^5^** |  |  |  |  |  |  |  |  |
| Psychiatrist | 54  (56) | 1·8  (1·2) | 140  (109) | 82  (109) | 26  (46) | 2·3  (1·2) | 185  (130) | 89  (129) |
| Psychologist | 23  (24) | 4·2  (3·1) | 251  (186) | 63  (143) | 6  (11) | 2·7  (1·0) | 155  (60) | 17  (52) |
| GP | 28  (29) | 2·3  (2·1) | 199  (209) | 61  (147) | 22  (38) | 3·0  (3·8) | 287  (565) | 117  (383) |
| District nurse | 3  (3) | 1·7  (1·2) | 159  (244) | 5  (46) | 1  (2) | 2·0  (-) | 49  (-) | 1  (7) |
| Crisis team member | 4  (4) | 5·3  (6·1) | 153  (167) | 7  (44) | 3  (5) | 3·0  (2·6) | 90  (65) | 5  (24) |
| Community psychiatric nurse | 67  (70) | 5·7  (5·2) | 292  (682) | 212  (595) | 35  (61) | 5·5  (4·2) | 170  (158) | 110  (151) |
| Social Worker | 6  (6) | 13·2  (25·5) | 349  (655) | 23  (176) | 3  (5) | 1·7  (1·2) | 79  (66) | 4  (22) |
| Occupational therapist | 3  (3) | 8·7  (4·2) | 240  (110) | 8  (46) | 3  (5) | 5·3  (5·9) | 1966  (3,124) | 109  (758) |
| Support worker | 20  (21) | 5·0  (3·6) | 211  (360) | 46  (186) | 6  (11) | 3·3  (2·1) | 66  (53) | 7  (27) |
| **Total Primary and care contacts^5^** | 84 | 10·3  (10·5) | 554  (780) | 507  (762) | 52 | 7·4  (5·8) | 478  (1,104) | 460  (1,087) |

^1 Mean number of days/ contacts for those who had at least one episode/ contact.^

^2 Mean duration of attendance/contacts for those who had at least one attendance/ contact.^

^3 Mean costs for those who had at least one attendance/contact.^

^4 Mean costs for the whole sample – (if not missing; code 888 or 999) (Community-based day services; Group CR (n=94), Independent CR (n=42), One-to-One CR (n=90), Treatment as Usual (n=51)); Primary and care contacts; (Group CR (n=104), Independent CR (n=48), One-to-One CR (n=92), Treatment as Usual (n=54)).^

^5 Mean total costs for those that used a service across all services.^**Post CR Resource Use: Criminal justice services**

|  | **Group CR (n=110)** | | | | **Independent CR (n=50)** | | | | **One-to-One CR (n=96)** | | | | **Treatment as Usual (n=57)** | | | |
| --- | --- | --- | --- | --- | --- | --- | --- | --- | --- | --- | --- | --- | --- | --- | --- | --- |
|  | **N (%) using** | **Mean**  **Days/**  **Contacts^1^** | **Mean Costs (SD)^2^** | **Mean Costs of total sample (SD)^3^** | **N (%) using** | **Mean**  **Days/**  **Contacts^1^** | **Mean Costs (SD)^2^** | **Mean Costs of total sample (SD)^3^** | **N (%) using** | **Mean**  **Days/**  **Contacts^1^** | **Mean Costs (SD)^2^** | **Mean Costs of total sample (SD)^3^** | **N (%) using** | **Mean**  **Days/**  **Contacts^1^** | **Mean Costs (SD)^2^** | **Mean Costs of total sample (SD)^3^** |
| **Over the last 6 months, has the patient been in**  **contact with the criminal justice services?** |  |  |  |  |  |  |  |  |  |  |  |  |  |  |  |  |
| How many contacts with the police?  (contact=interview or stay of some hours but not overnight) | 3  (3) | 2·0  (1·0) | 126  (63) | 4  (24) | 4  (8) | 1·0  (0) | 63  (0) | 6  (18) | 3  (3) | 1·0  (0) | 63  (0) | 2  (12) | 5  (9) | 1·6  (1·3) | 101  (85) | 11  (40) |
| How many nights spent in a police cell or prison? | 1  (1) | 4·0  (-) | 386  (-) | 4  (39) | 1  (2) | 1·0  (-) | 1,044  (-) | 24  (157) | 0  (0) | - | - | - | 3  (5) | 62·7  (50·0) | 6,050  (4,824) | 378  (1,784) |
| How many psychiatric assessments whilst in custody? | 1  (1) | 1·0  (-) | 261  (-) | 3  (26) | 0  (0) | - | - | 0  (0) | 1  (1) | 1·0  (-) | 261  (-) | 3  (28) | 2  (4) | 3·0  (0) | 783  (0) | 33  (158) |
| How many criminal court appearances? | 1  (1) | 1·0  (-) | 44  (-) | 0  (4) | 0  (0) | - | - | 0  (0) | 1  (1) | 1·0  (-) | 44  (-) | 1  (5) | 4  (7) | 1·8  (1·0) | 76  (42) | 6  (24) |
| How many civil court appearances? | 0  (0) | - | - | 0  (0) | 0  (0) | - | - | 0  (0) | 0  (0) | - | - | - | 0  (0) | - | - | 0  (0) |
| **Total** | 3 | - | 356  (404) | 11  (85) | 4 | - | 324  (522) | 29  (167) | 3 | - | 165  (140) | 6  (37) | 5 | - | 4,106  (5281) | 428  (1,995) |

^1 Mean number of days/ contacts for those who had at least one episode/ contact.^

^2 Mean costs for those who had at least one episode/contact.^

^3 Mean costs for the whole sample-(Group CR (n=98), Independent CR (n=44), One-to-One CR (n=86), Treatment as Usual (n=48)).^**6 Month Follow-up Resource Use: Inpatient hospital services and outpatient hospital services**

| **Inpatient hospital services** | **Group CR (n=97)** | | | | **Independent CR (n=46)** | | | | **One-to-One CR (n=86)** | | | | **Treatment as Usual (n=55)** | | | |
| --- | --- | --- | --- | --- | --- | --- | --- | --- | --- | --- | --- | --- | --- | --- | --- | --- |
|  | **N (%) using** | **Mean**  **Days/**  **Contacts^1^** | **Mean Costs (SD)^2^** | **Mean Costs of total sample (SD)^3^** | **N (%) using** | **Mean**  **Days/**  **Contacts^1^** | **Mean Costs (SD)^2^** | **Mean Costs of total sample (SD)^3^** | **N (%) using** | **Mean**  **Days/**  **Contacts^1^** | **Mean Costs (SD)^2^** | **Mean Costs of total sample (SD)^3^** | **N (%) using** | **Mean**  **Days/**  **Contacts^1^** | **Mean Costs (SD)^2^** | **Mean Costs of total sample (SD)^3^** |
| Acute psychiatric ward | 3  (3) | 57·3  (35·1) | 24,309  (14,872) | 848  (5,033) | 1  (2) | 43·0  (-) | 18,232  (-) | 445  (2,847) | 4  (5) | 35·0  (26·7) | 14,840  (11,330) | 761  (3,982) | 4  (7) | 40·0  (55·2) | 16,960  (23,391) | 1,384  (7,497) |
| Psychiatric Rehabilitation ward | 0  (0) | - | - | 0  (0) | 0  (0) | - | - | 0  (0) | 0  (0) | - | - | 0  (0) | 0  (0) | - | - | 0  (0) |
| Long-stay ward | 2  (2) | 60·5  (43·1) | 25,652  (18,289) | 597  (4,366) | 0  (0) | - | - | 0  (0) | 1  (1) | 28·0  (-) | 11,872  (-) | 152  (1,344) | 0  (0) | - | - | 0  (0) |
| Emergency/Crisis centre | 0  (0) | - | - | 0  (0) | 0  (0) | - | - | 0  (0) | 1  (1) | 1  (-) | 424  (-) | 5  (48) | 0  (0) | - | - | 0  (0) |
| General medical ward | 1  (1) | 1·0  (-) | 602  (-) | 7  (65) | 2  (4) | 17·5  (23·3) | 10,535  (14,047) | 514  (3,196) | 2  (2) | 2·5  (2·1) | 1,505  (1,277) | 39  (280) | 1  (2) | 3·0  (-) | 1,806  (-) | 37  (258) |
| Other | 1  (1) | 2·0  (-) | 848  (-) | 10  (91) | 0  (0) | - | - | 0  (0) | 0  (0) | - | - | 0  (0) | 0  (0) | - | - | 0  (0) |
| **Total inpatient costs across all services^4^** | 7 | 42·3  (38·7) | 17,955  (16,382) | 1,461  (6,583) | 3 | 26·0  (22·1) | 13,101  (10,882) | 959  (4,225) | 6 | 29·0  (24·4) | 12,444  (10,287) | 957  (4,244) | 5 | 32·6  (50·6) | 13,929  (21,360) | 1,421  (7,495) |
| **Outpatient hospital services** |  |  |  |  |  |  |  |  |  |  |  |  |  |  |  |  |
| Psychiatric outpatient visit | 2  (2) | 4·5  (2·1) | 608  (286) | 14  (98) | 2  (4) | 2·5  (2·1) | 338  (286) | 16  (86) | 0  (0) | - | - | 0  (0) | 2  (4) | 18·5  (7·8) | 2,498  (1,050) | 100  (517) |
| Other hospital outpatient visit (including A&E) | 13  (13) | 1·2  (0·6) | 166  (81) | 25  (67) | 3  (7) | 1·0  (0) | 135  (0) | 10  (36) | 10  (12) | 1·2  (0·6) | 162  (85) | 21  (63) | 8  (15) | 1·9  (1·7) | 253  (233) | 41  (129) |
| Day hospital | 0  (0) | - | - | 0  (0) | 2  (4) | 18·0  (17·0) | 7,236  (6822) | 353  (1,912) | 2  (2) | 6·5  (7·8) | 2,613  (3,127) | 69  (555) | 2  (4) | 9·5  (12·0) | 3,819  (4,832) | 153  (1,024) |
| Clozapine clinic | 2  (2) | 6·0  (0) | 810  (0) | 19  (124) | 2  (4) | 12·5  (16·3) | 1,688  (2,196) | 82  (506) | 2  (2) | 13·5  (9·2) | 1,823  (1,241) | 48  (327) | 0  (0) | - | - | 0  (0) |
| Other | 3  (3) | 2·7  (2·9) | 360  (390) | 13  (90) | 1  (2) | 1·0  (-) | 135  (-) | 3  (21) | 4  (5) | 1·5  (0·6) | 203  (78) | 11  (48) | 2  (4) | 43·0  (18·4) | 5,805  (2,482) | 232  (1,203) |
| **Total Outpatient costs across all services^4^** | 17 | 2·6  (2·4) | 357  (324) | 71  (202) | 10 | 7·0  (10·8) | 1,906  (3,739) | 465  (1,958) | 15 | 3·9  (8·2) | 753  (1,923) | 149  (884) | 13 | 12·1  (16·6) | 2,021  (2,717) | 525  (1,615) |

^1 Mean number of days/ contacts for those who had at least one episode/ contact.^

^2 Mean costs for those who had at least one episode/contact.^

^3 Mean costs for the whole sample – (if not missing; code 888 or 999) (Inpatient admissions; Group CR (n=86), Independent CR (n=41), One-to-One CR (n=78), Treatment as Usual (n=49)); (Outpatient contacts; (Group CR (n=85), Independent CR (n=41), One-to-One CR (n=76), Treatment as Usual (n=50)).^

^4 Mean total costs for those that used a service across all services.^

**6 Month Follow-up Resource Use: Community-based day services and primary and care contacts**

|  | **Group CR (n=97)** | | | | **Independent CR (n=46)** | | | |
| --- | --- | --- | --- | --- | --- | --- | --- | --- |
| **Community-based day services** | **N (%) using** | **Mean**  **Days/**  **Contacts^1^** | **Mean Costs (SD)^3^** | **Mean Costs of total sample (SD)^4^** | **N (%) using** | **Mean**  **Days/**  **Contacts^1^** | **Mean Costs (SD)^3^** | **Mean Costs of total sample (SD)^4^** |
| Community mental health centre | 3  (3) | 7·7  (7·6) | 317  (315) | 12  (78) | 3  (7) | 21·0  (24·3) | 3,993  (3,965) | 299  (1,393) |
| Day care centre | 1  (1) | 5·0  (-) | 1,033  (-) | 13  (115) | 0  (0) | - | - | 0  (0) |
| Sheltered workshop | 0  (0) | - | - | 0  (0) | 1  (2) | 24·0  (-) | 2,765  (-) | 69  (437) |
| Specialist education / Recovery college | 0  (0) | - | - | 0  (0) | 2  (4) | 14·5  (13·4) | 2,352  (68) | 118  (519) |
| **Total Community-based day services costs across all services^5^** | 4 | 7·0  (6·4) | 496  (441) | 24  (138) | 4 | 29·0  (29·8) | 4,862  (3,650) | 486  (1,791) |
| **Primary and care contacts** |  |  |  |  |  |  |  |  |
| Psychiatrist | 55  (57) | 2·7  (3·0) | 228  (337) | 141  (286) | 26  (57) | 3·5  (4·5) | 196  (178) | 113  (166) |
| Psychologist | 26  (27) | 7·0  (6·2) | 737  (2,347) | 215  (1,296) | 11  (24) | 7·7  (8·8) | 302  (404) | 74  (233) |
| GP | 39  (40) | 1·9  (1·6) | 110  (77) | 48  (75) | 20  (43) | 2·9  (2·9) | 141  (98) | 63  (96) |
| District nurse | 1  (1) | 12·0  (-) | 196  (-) | 2  (21) | 0  (0) | - | - | 0  (0) |
| Crisis team member | 8  (8) | 4·8  (5·1) | 103  (125) | 9  (46) | 1  (2) | 10·0  (-) | 360  (-) | 8  (54) |
| Community psychiatric nurse | 64  (66) | 7·4  (6·2) | 286  (436) | 206  (391) | 32  (70) | 9·0  (8·2) | 415  (857) | 295  (744) |
| Social Worker | 1  (1) | 2·0  (-) | 102  (-) | 1  (11) | 4  (9) | 4·8  (4·9) | 147  (107) | 13  (51) |
| Occupational therapist | 2  (2) | 4·0  (4·2) | 129  (174) | 3  (27) | 3  (7) | 13·0  (10·5) | 396  (409) | 26  (133) |
| Support worker | 18  (19) | 9·1  (9·1) | 358  (858) | 72  (404) | 14  (30) | 8·6  (5·4) | 226  (185) | 70  (146) |
| **Total Primary and care contacts^5^** | 83 | 13·3  (10·8) | 748  (1,721) | 698  (1,672) | 43 | 16·5  (15·3) | 693  (1,070) | 662  (1,055) |

^1 Mean number of days/ contacts for those who had at least one episode/ contact.^

^2 Mean duration of attendance/contacts for those who had at least one attendance/ contact.^

^3 Mean costs for those who had at least one attendance/contact.^

^4 Mean costs for the whole sample – (if not missing; code 888 or 999) (Community-based day services; Group CR (n=81), Independent CR (n=40), One-to-One CR (n=75), Treatment as Usual (n=48)); Primary and care contacts; (Group CR (n=89), Independent CR (n=45), One-to-One CR (n=82), Treatment as Usual (n=51)).^

^5 Mean total costs for those that used a service across all services.^

**6 Month Follow-up Resource Use: Community-based day services and primary and care contacts (continued)**

|  | **One-to-One CR (n=86)** | | | | **Treatment as Usual (n=55)** | | | |
| --- | --- | --- | --- | --- | --- | --- | --- | --- |
| **Community-based day services** | **N (%) using** | **Mean**  **Days/**  **Contacts^1^** | **Mean Costs (SD)^3^** | **Mean Costs of total sample (SD)^4^** | **N (%) using** | **Mean**  **Days/**  **Contacts^1^** | **Mean Costs (SD)^3^** | **Mean Costs of total sample (SD)^4^** |
| Community mental health centre | 3  (3) | 1·3  (0·6) | 35  (42) | 1  (10) | 2  (4) | 13·0  (9·9) | 1,0734  (818) | 45  (247) |
| Day care centre | 0  (0) | - | - | 0  (0) | 0  (0) | - | - | 0  (0) |
| Sheltered workshop | 1  (1) | 1·0  (-) | 48  (-) | 1  (6) | 0  (0) | - | - | 0  (0) |
| Specialist education / Recovery college | 0  (0) | - | - | 0  (0) | 0  (0) | - | - | 0  (0) |
| **Total Community-based day services costs across all services** | 4 | 1·3  (0·5) | 38  (35) | 2  (11) | 2 | 13·0  (9·9) | 1,074  (818) | 45  (247) |
| **Primary and care contacts** |  |  |  |  |  |  |  |  |
| Psychiatrist | 52  (60) | 2·7  (2·5) | 173  (210) | 110  (187) | 24  (44) | 2·7  (2·2) | 187  (189) | 88  (159) |
| Psychologist | 20  (23) | 7·6  (6·1) | 459  (372) | 112  (268) | 10  (18) | 10·1  (4·7) | 583  (274) | 114  (261) |
| GP | 30  (35) | 1·9  (1·6) | 155  (124) | 57  (106) | 26  (47) | 8·3  (9·9) | 1136  (1,948) | 579  (1,492) |
| District nurse | 2  (2) | 1·0  (0) | 14  (3) | 0  (2) | 1  (2) | 2·0  (-) | 98  (-) | 2  (14) |
| Crisis team member | 4  (5) | 5·0  (3·6) | 84  (62) | 4  (22) | 0  (0) | - | - | 0  (0) |
| Community psychiatric nurse | 68  (79) | 9·0  (8·2) | 354  (521) | 294  (493) | 35  (64) | 6·2  (4·9) | 239  (242) | 164  (229) |
| Social Worker | 4  (5) | 3·8  (5·5) | 174  (292) | 9  (68) | 7  (13) | 7·9  (9·4) | 267  (272) | 37  (132) |
| Occupational therapist | 2  (2) | 5·0  (1·4) | 28  (29) | 1  (5) | 4  (7) | 2·0  (0·8) | 81  (34) | 6  (24) |
| Support worker | 13  (15) | 7·2  (7·2) | 318  (601) | 50  (259) | 6  (11) | 7·2  (8·6) | 129  (125) | 15  (58) |
| **Total Primary and care contacts** | 77 | 14·3  (11·2) | 677  (696) | 636  (694) | 48 | 14·7  (14·1) | 1,068  (1,754) | 1,005  (1,719) |

^1 Mean number of days/ contacts for those who had at least one episode/ contact.^

^2 Mean duration of attendance/contacts for those who had at least one attendance/ contact.^

^3 Mean costs for those who had at least one attendance/contact.^

^4 Mean costs for the whole sample – (if not missing; code 888 or 999) (Community-based day services; Group CR (n=81), Independent CR (n=40), One-to-One CR (n=75), Treatment as Usual (n=48)); Primary and care contacts; (Group CR (n=89), Independent CR (n=45), One-to-One CR (n=82), Treatment as Usual (n=51)).^

^5 Mean total costs for those that used a service across all services.^

**6 Month Follow-up Resource Use: Criminal justice services**

|  | **Group CR (n=97)** | | | | **Independent CR (n=46)** | | | | **One-to-One CR (n=86)** | | | | **Treatment as Usual (n=55)** | | | |
| --- | --- | --- | --- | --- | --- | --- | --- | --- | --- | --- | --- | --- | --- | --- | --- | --- |
|  | **N (%) using** | **Mean**  **Days/**  **Contacts^1^** | **Mean Costs (SD)^2^** | **Mean Costs of total sample (SD)^3^** | **N (%) using** | **Mean**  **Days/**  **Contacts^1^** | **Mean Costs (SD)^2^** | **Mean Costs of total sample (SD)^3^** | **N (%) using** | **Mean**  **Days/**  **Contacts^1^** | **Mean Costs (SD)^2^** | **Mean Costs of total sample (SD)^3^** | **N (%) using** | **Mean**  **Days/**  **Contacts^1^** | **Mean Costs (SD)^2^** | **Mean Costs of total sample (SD)^3^** |
| **Over the last 6 months, has the patient been in**  **contact with the criminal justice services?** |  |  |  |  |  |  |  |  |  |  |  |  |  |  |  |  |
| How many contacts with the police?  (contact=interview or stay of some hours but not overnight) | 4  (4) | 1·5  (1·0) | 95  (63) | 5  (24) | 2  (4) | 1·5  (0·7) | 95  (45) | 5  (22) | 2  (2) | 1·0  (0) | 63  (0) | 2  (10) | 5  (9) | 1·4  (0·5) | 88  (35) | 10  (31) |
| How many nights spent in a police cell or prison? | 2  (2) | 2·5  (2·1) | 715  (465) | 17  (122) | 2  (4) | 5·5  (2·1) | 531  (205) | 25  (119) | 0  (0) | - | - | 0  (0) | 2  (4) | 45·0  (21·2) | 4,345  (2,048) | 202  (978) |
| How many psychiatric assessments whilst in custody? | 2  (2) | 1·0  (0) | 261  (0) | 6  (41) | 1  (2) | 2·0  (-) | 522  (-) | 12  (81) | 2  (2) | 1·0  (0) | 261  (0) | 7  (43) | 1  (2) | 2·0  (-) | 522  (-) | 12  (12) |
| How many criminal court appearances? | 2  (2) | 2·0  (1·4) | 87  (62) | 2  (15) | 0  (0) | - | - | 0  (0) | 0  (0) | - | - | 0  (0) | 3  (5) | 2·0  (1·0) | 87  (44) | 6  (24) |
| How many civil court appearances? | 0  (0) | - | - | 0  (0) | 0  (0) | - | - | 0  (0) | 0  (0) | - | - | 0  (0) | 0  (0) | - | - | 0  (0) |
| **Total** | 5 | - | 501  (504) | 31  (165) | 2 | - | 887  (209) | 42  (194) | 2 | - | 324  (0) | 89  (53) | 6 | - | 1,653  (2,605) | 231  (1,069) |

^1 Mean number of days/ contacts for those who had at least one episode/ contact.^

^2 Mean costs for those who had at least one episode/contact.^

^3 Mean costs for the whole sample-(Group CR (n=82), Independent CR (n=42), One-to-One CR (n=74), Treatment as Usual (n=43)).^

Table 23s show all service use data for each trial arm: The main cost drivers were inpatient hospital service use.

**Table 24s Health economic primary and secondary outcomes at each time point**

| **Table 24s:**  **EQ5D-3L Scores, GAS scores at each timepoint** | | | | | | | | |
| --- | --- | --- | --- | --- | --- | --- | --- | --- |
| **Outcome** | **Group CR (n=134)** | | **Independent CR (n=65)** | | **One-to-One CR (n=112)** | | **Treatment as Usual (n=66)** | |
|  | **N (%)** | **Mean (SD)** | **N (%)** | **Mean (SD)** | **N (%)** | **Mean (SD)** | **N (%)** | **Mean (SD)** |
| **Baseline**  **EQ5D-3L** | 134 (100) | 0·7505 (0·2146) | 64 (98) | 0·7509 (0·2377) | 112 (100) | 0·7077 (0·2309) | 66 (100) | 0·7892 (0·2270) |
| **Baseline GAS** | 130 (97) | 33·46 (4·58) | 64 (98) | 33·93 (4·37) | 110 (98) | 32·48 (5·11) | 66 (100) | 33·78 (4·39) |
| **Post Therapy EQ5D-3L** | 89 (66) | 0·8051 (0·1742) | 34 (52) | 0·7285 (0·2343) | 82 (73) | 0·7353 (0·2930) | 42 (64) | 0·8013 (0·2377) |
| **Post Therapy GAS** | 92 (69) | 52·14 (11·06) | 36 (55) | 46·74 (9·64) | 83 (74) | 50·87 (12·01) | 43 (65) | 46·60 (12·20) |
| **Follow-up EQ5D-3L** | 75 (56) | 0·7955 (0·1686) | 32 (49) | 0·8076 (0·2342) | 61 (54) | 0·7779 (0·2438) | 38 (58) | 0·7205 (0·2703) |
| **Follow-up GAS** | 77 (57) | 53·73 (12·06) | 31 (48) | 49·97 (11·52) | 65 (58) | 52·96 (11·43) | 35 (53) | 50·67 (16·28) |

Table 24s shows the EQ5D-3L Scores, GAS scores at each timepoint. Follow-up EQ5D-3L scores were higher than baseline EQ5D-3L scores for all arms except TAU. Follow-up GAS scores were higher in all arms compared to baseline GAS scores.

| **Table 25s**  **Complete case analysis: Broader costs, outcome, and cost effectiveness results** | | | |
| --- | --- | --- | --- |
| **QALY Outcome** | | | |
| **Group CR vs** **One-to-One CR** | (n=58) | (n=55) | Incremental cost/QALY gained (95%CI)^2^ |
| Costs (Broader perspective)^5^ | 7,209 (15,252) | 6,663 (7,871) | -265 (-1,429 to 1,309) |
| QALYs^3^ | 0·5979 (0·1064) | 0·5682 (0·1484) | 0·0068 (-0·0246 to 0·0366) |
| Broader perspective: costs (£) per QALY gain (ICER)^4,5^ | | | **Group CR Dominates One-to-One CR*** |
| **Group CR vs TAU** | (n=58) | (n=28) | Incremental cost/ QALY gained (95%CI)^2^ |
| Costs (Broader perspective)^5^ | 7,209 (15,252) | 6,175 (9,119) | -57 (-2,284 to 2,244) |
| QALYs^3^ | 0·5979 (0·1064) | 0·4928 (0·1970) | 0·0690 (0·01945 to 0·1083) |
| Broader perspective: costs (£) per QALY gain (ICER)^4,5^ | | | **Group CR Dominates TAU*** |
| **One-to-One CR vs TAU** | (n=55) | (n=28) | Incremental cost/QALY gained (95%CI)^2^ |
| Costs (Broader perspective)^5^ | 6,663 (7,871) | 6,175 (9,119) | 421 (-1,666 to 2,421) |
| QALYs^3^ | 0·5682 (0·1484) | 0·4928 (0·1970) | 0·0860 (0·0438 to 0·1232) |
| Broader perspective: costs (£) per QALY gain (ICER)^4,5^ | | | **4,899** |
| **GAS Score Outcome** | | | |
| **Group CR vs One-to-One CR** | (n=62) | (n=57) | Incremental cost/GAS score unit gained^2^ |
| Costs (Broader perspective)^5^ | 7,342 (14,841) | 6,384 (7,695) | -38 (-1,219 to 1,412) |
| GAS Score | 55·45 (11·48) | 53·34 (11·37) | 1·96 (-1·92 to 5·83) |
| Broader perspective: costs (£) per additional unit on the GAS (ICER)^4,5^ | | | **Group CR Dominates One-to-One CR*** |
| **Group CR vs TAU** | (n=62) | (n=28) | Incremental cost/ GAS score unit gained^2^ |
| Costs (Broader perspective)^5^ | 7,342 (14,841) | 6,087 (9,152) | 251 (-2,015 to 2,692) |
| GAS Score | 55·45 (11·48) | 52·43 (16·51) | 3·45 (-3·22 to 10·33) |
| Broader perspective: costs (£) per additional unit on the GAS (ICER)^4,5^ | | | **73** |
| **One-to-One CR vs TAU** | (n=57) | (n=28) | Incremental cost/GAS score unit gained^2^ |
| Costs (Broader perspective)^5^ | 6,384 (7,695) | 6,087 (9,152) | 300 (-1,610 to 2,329) |
| GAS Score | 53·34 (11·37) | 52·43 (16·51) | 0·4275 (-7·28 to 8·13) |
| Broader perspective: costs (£) per additional unit on the GAS (ICER)^4,5^ | | | **701** |

1 NHS PSS costs (includes inpatient hospital services, outpatient hospital services, community-based day services, primary and care contacts).

2 Adjusted for baseline costs (or baseline utility), treatment arm, site, and period.

3 Using QALYS from crosswalk.

4 ICER = Incremental cost-effectiveness ratio (difference in costs divided by the difference in outcome of the two groups).

5 Total costs (NHS PSS costs, criminal justice system costs, time spent in employment).

*Using point estimates, the dominant treatment option is both less costly and results in greater health outcomes than the comparator group.

Table 25s shows the results from the complete case analysis adopting a broader perspective.

**Group CR vs One-to-One:**

Group CR had lower costs compared to One-to-One but the difference in costs was not significant -£265 (-£1,429 to £1,309). The difference in QALYs was also not significant 0.0068 (-0.0246 to 0.0366), although Group CR had higher QALYs than One-to-One. Group CR therefore dominates One-to-One as it is less expensive and more effective. Group CR also dominates One-to-One when using the GAS score outcome measure, although no significant differences in costs or GAS scores were observed between groups.

**Group CR vs TAU:**

Group CR had lower costs compared to TAU but the difference in costs was not significant -£57 (-£2,284 to £2,244). The difference in QALYs was significant between groups 0.0690 (0.01945 to 0.1083). Group CR therefore dominates TAU as it is less expensive and more effective. Group CR had higher costs compared to TAU when using the GAS Score outcome measure, but the difference in costs was not significant. The difference in GAS score was also not significant between groups although Group CR had higher GAS scores compared to TAU.

**One-to-One vs TAU:**

One-to-One had higher costs compared to TAU but the difference in costs was not significant £421 (-£1,666 to £2,421). The difference in QALYs was significant between groups 0.0860 (0.0438 to 0.1232) (One-to-One had higher QALYs than TAU). The ICER was £4,899 per QALY; lower than the threshold used by NICE in recommending interventions. It was the same trend for the GAS score; however, differences were not significant.


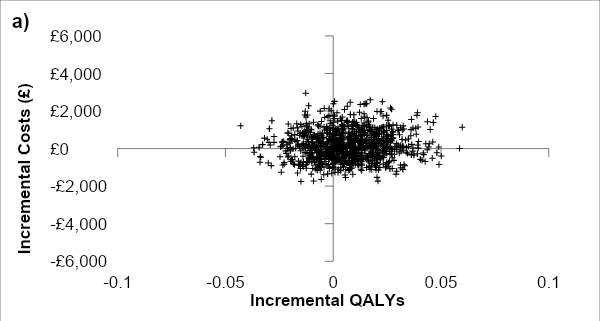

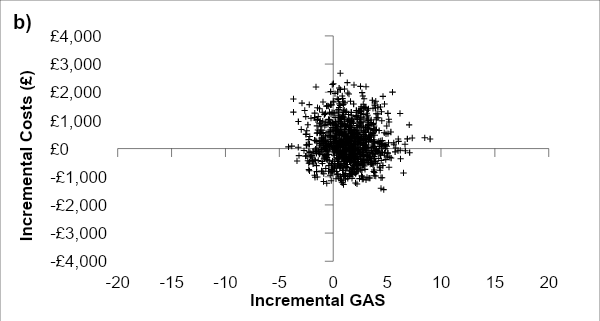


Figure 2s Bootstrapped replicates of incremental cost and incremental outcomes (Group CR vs One-to-One CR) a) QALY outcome b) GAS Score Outcome
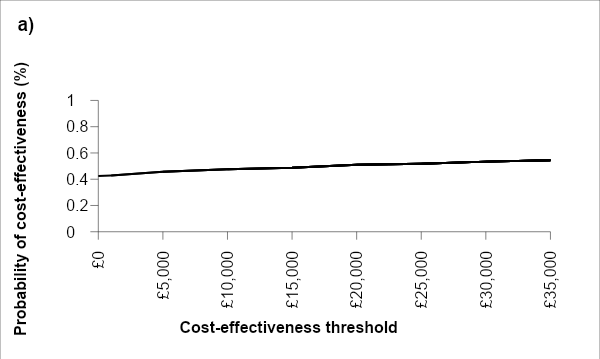


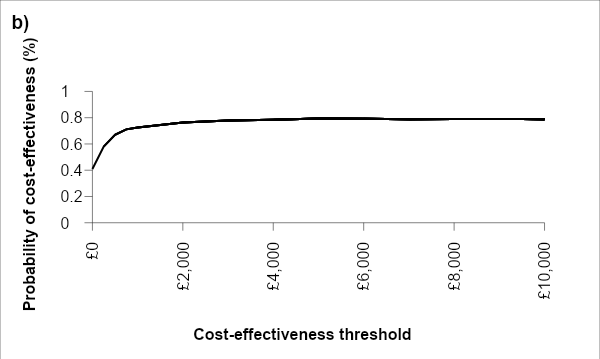


Figure 3s Cost-effectiveness acceptability curve (Group CR vs One-to-One CR) a) QALY outcome b) GAS Score Outcome


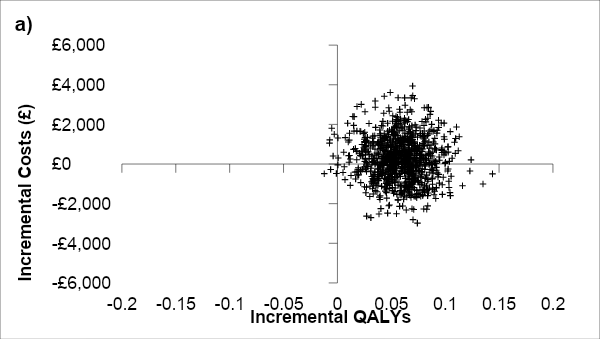

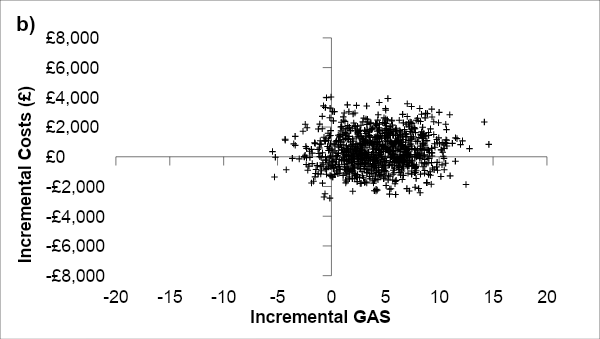


##

## Figure 3s Bootstrapped replicates of incremental cost and incremental outcomes (Group CR vs TAU) a) QALY outcome b) GAS Score Outcome


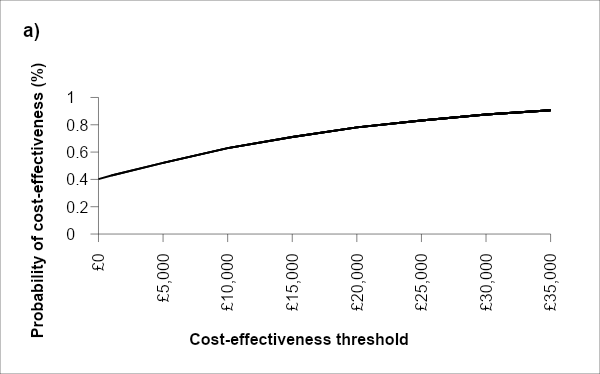

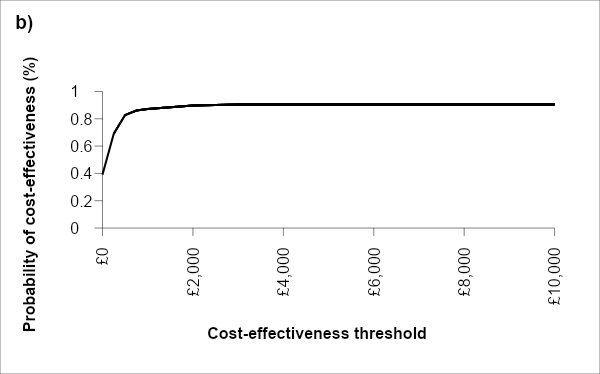


## Figure 3s Cost-effectiveness acceptability curve (Group CR vs TAU) a) QALY outcome b) GAS Score Outcome


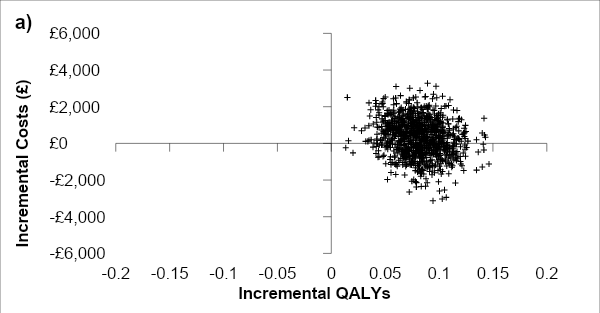

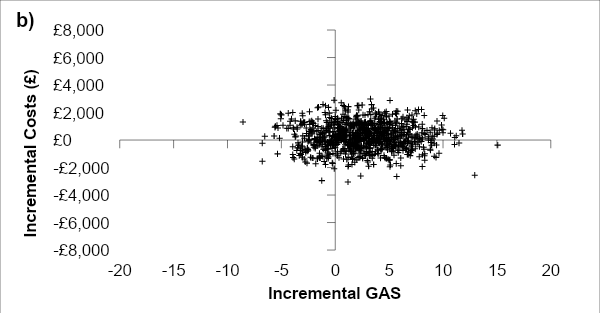


Figure 2s Bootstrapped replicates of incremental cost and incremental outcomes (One-to-One CR vs TAU) a) QALY outcome b) GAS Score Outcome
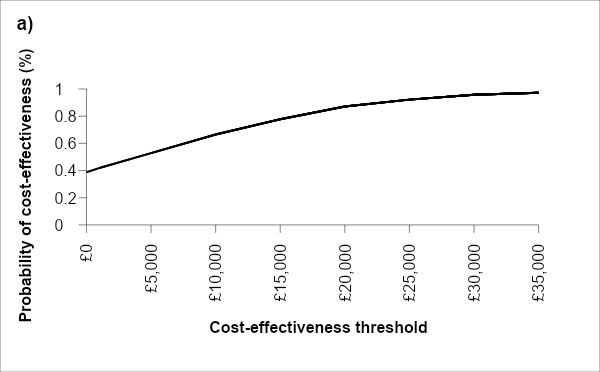


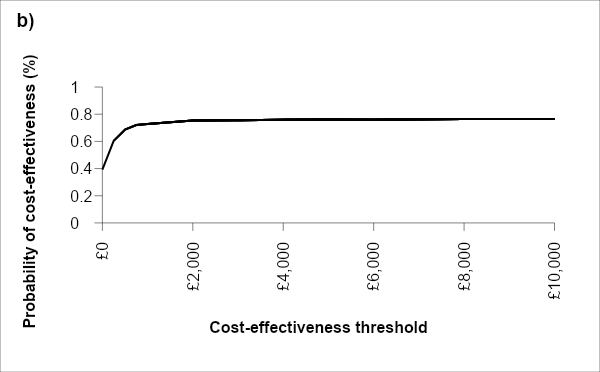


Figure 3s Cost-effectiveness acceptability curve (One-to-One CR vs TAU) a) QALY outcome b) GAS Score Outcome

Group CR vs One-to-One and Group CR vs TAU: The replications on the cost effectiveness plane are not confined to one quadrant, indicating uncertainty around the results.

One-to-One vs TAU: The majority of the replications are confined to the north-east and south-east quadrant, demonstrating that One-to-One produces more QALYs than TAU. When using the GAS score measure, the replications are not confined to one quadrant, indicating uncertainty around the results indicating around the results.

**
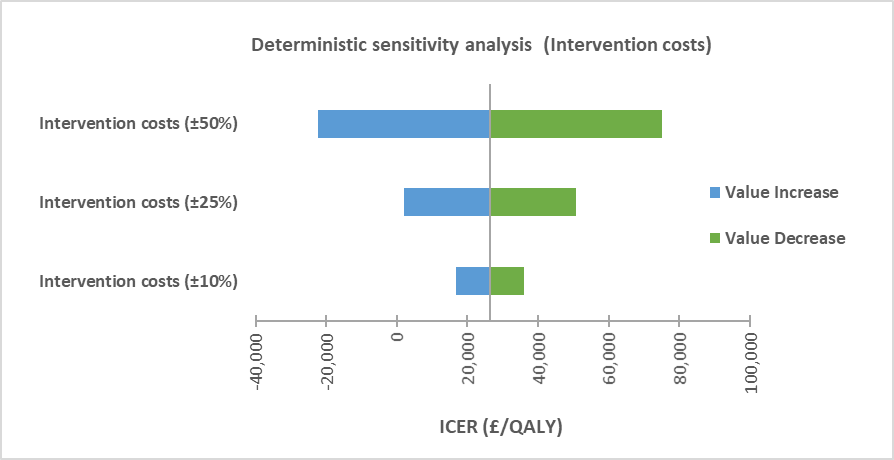
Group CR vs One-to-One CR**

Figure 4s: Deterministic sensitivity analysis; varying the intervention costs upwards and downwards by 10, 25, and 50%. (Base case = £26,383)

Table 26s: One-way sensitivity analysis of cost-effectiveness results for Group CR vs One-to-One CR

| **Table 12s** | | | | |
| --- | --- | --- | --- | --- |
| **Scenario** | **Description** | **Difference in Costs (£)** | **Difference in QALYs** | **ICER (£/QALY)** |
| Base case |  | 150 | 0·0057 | 26,383 |
| Scenario 1 | Therapist Band 5 | 60 | 0·0057 | 10,649 |
| Scenario 2 | Training 20 hours | 150 | 0·0057 | 26,383 |
| Scenario 3 | Training 25 hours | 150 | 0·0057 | 26,383 |
| Scenario 4 | Band 8a | 150 | 0·0057 | 26,383 |
| Scenario 5 | Circuits access £19 | 150 | 0·0057 | 26,383 |
| Scenario 6 | Circuits access £25 | 150 | 0·0057 | 26,383 |
| Scenario 7 | ED-5D-5L ratings | 150 | 0·0042 | 35,863 |

For Group CR vs One-to-One CR, the ICER was most sensitive in scenario 1 and scenario 7. In scenario 1, the ICER decreased to £10,649 when the unit cost of a therapist was increased to a Band 5 therapist of (£36 per hour). In addition, the value of the ICER increased to £35,863 in scenario 7 when using the 5L tariffs for the EQ-5D-5L.

**Group CR vs TAU**

**
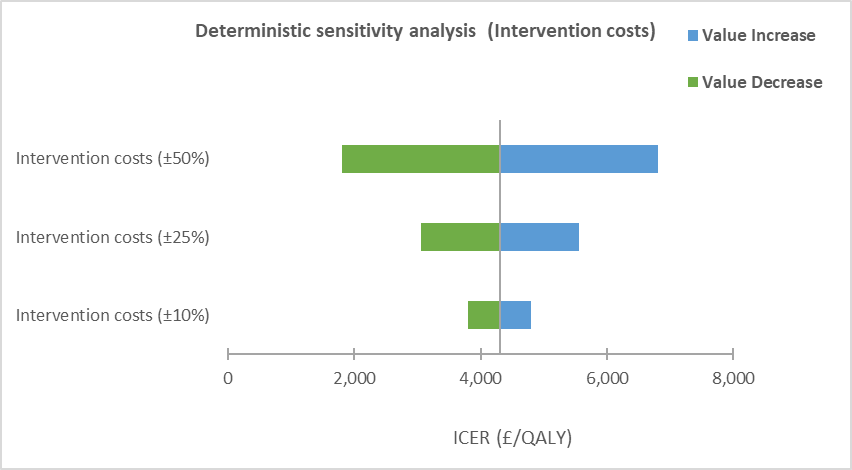
**

Figure 4s: Deterministic sensitivity analysis; varying the intervention costs upwards and downwards by 10, 25, and 50%. (Base case = £4,306)

Table 27s: One-way sensitivity analysis of cost-effectiveness results for Group CR vs TAU

| **Scenario** | **Description** | **Difference in Costs (£)** | **Difference in QALYs** | **ICER (£/QALY)** |
| --- | --- | --- | --- | --- |
| Base case |  | 257 | 0·0597 | 4,306 |
| Scenario 1 | Therapist Band 5 | 287 | 0·0597 | 4,816 |
| Scenario 2 | Training 20 hours | 251 | 0·0597 | 4,209 |
| Scenario 3 | Training 25 hours | 261 | 0·0597 | 4,371 |
| Scenario 4 | Band 8a | 248 | 0·0597 | 4,159 |
| Scenario 5 | Circuits access £19 | 253 | 0·0597 | 4,248 |
| Scenario 6 | Circuits access £25 | 259 | 0·0597 | 4,348 |
| Scenario 7 | ED-5D-5L ratings | 257 | 0·0443 | 5,801 |

For Group CR vs TAU, the ICER was most sensitive in scenario 7. The value of the ICER increased to £5,801 when using the 5L tariffs for the EQ-5D-5L.

**One-to-One CR vs TAU**


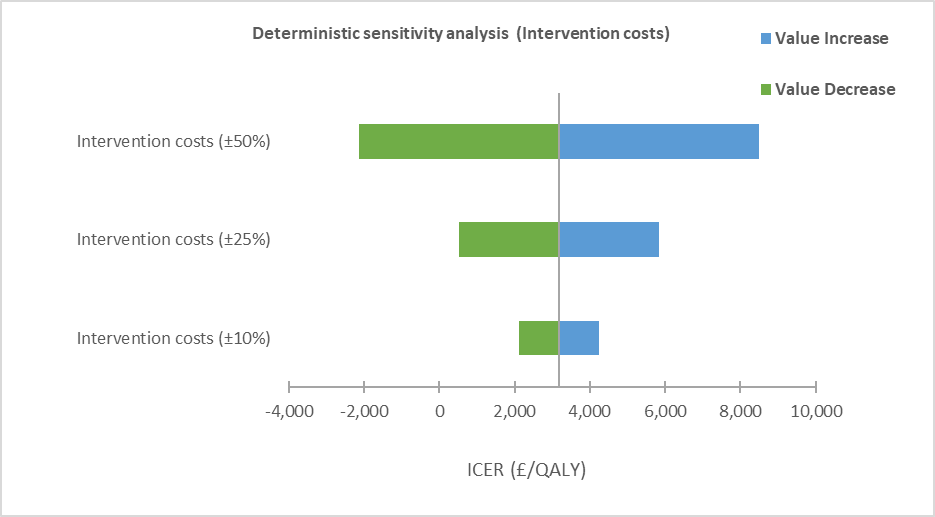


Figure 4s: Deterministic sensitivity analysis; varying the intervention costs upwards and downwards by 10, 25, and 50%. (Base case = £3,170)

Table 28s: One-way sensitivity analysis of cost-effectiveness results for One-to-One CR vs TAU

| **Scenario** | **Description** | **Difference in Costs (£)** | **Difference in QALYs** | **ICER (£/QALY)** |
| --- | --- | --- | --- | --- |
| Base case |  | 260 | 0·0821 | 3,170 |
| Scenario 1 | Therapist Band 5 | 383 | 0·0821 | 4,671 |
| Scenario 2 | Training 20 hours | 254 | 0·0821 | 3,100 |
| Scenario 3 | Training 25 hours | 264 | 0·0821 | 3,218 |
| Scenario 4 | Band 8a | 251 | 0·0821 | 3,063 |
| Scenario 5 | Circuits access £19 | 257 | 0·0821 | 3,128 |
| Scenario 6 | Circuits access £25 | 263 | 0·0821 | 3,201 |
| Scenario 7 | ED-5D-5L ratings | 260 | 0·0579 | 4,494 |

For One-to-One CR vs TAU, the ICER was most sensitive in scenario 1 and scenario 7. In scenario 1, the ICER increased to £4,671when the unit cost of a therapist was increased to a Band 5 therapist of (£36 per hour). Additionally, the value of the ICER increased to £4,494 in scenario 7 when using the 5L tariffs for the EQ-5D-5L.

**11. e-references**

Barnett JH, Robbins TW, Leeson VC, Sahakian BJ, Joyce EM, Blackwell AD. Assessing cognitive function in clinical trials of schizophrenia. Neuroscience & Biobehavioral Reviews. 2010 Jul 1;34(8):1161-77.

Beecham, J. and M. Knapp (1995). The client service receipt inventory, Discussion Paper 1492. Personal Social Services Research Unit, University of Kent at Canterbury.

Blanchard, J. J., K. R. Bradshaw, C. P. Garcia, H. A. Nasrallah, P. D. Harvey, D. Casey, C. T. Csoboth, J. I. Hudson, L. Julian and E. Lentz (2017). "Examining the reliability and validity of the Clinical Assessment Interview for Negative Symptoms within the Management of Schizophrenia in Clinical Practice (MOSAIC) multisite national study." Schizophrenia research **185**: 137-143.

Blanchard, J. J., R. E. Gur, W. P. Horan and A. M. Kring (2012). "Manual for the clinical assessment interview for Negative symptoms (CAINS)." CANSAS Collaborative Group.

Bouwens et al. Review of Goal Attainment Scaling as a Useful Outcome Measure in Psychogeriatric Patients with Cognitive Disorders. Dement Geriatr Cogn Disord 2008;26:528–540

Burton et al. Clinical and cost-effectiveness of a new psychosocial intervention to support independence in Dementia (NIDUS-family) for family carers and people living with dementia in their own homes: a randomised controlled trial. Trials (2021) 22:865.

Chew et al Outcomes of a multimodal cognitive and physical rehabilitation program for persons with mild dementia and their caregivers: a goal-oriented approach. Clinical Interventions in Aging 2015:10 1687–1694

Evans, 2012 Goal setting during rehabilitation early and late after acquired brain injury Curr Opin Neurol 2012, 25:651–655

EuroQol Group. EuroQol-a new facility for the measurement of health-related quality of life. Health policy. 1990;16(3):199-208

Freidlin, B., E. L. Korn, R. Gray and A. Martin (2008). "Multi-arm clinical trials of new agents: some design considerations." Clinical Cancer Research **14**(14): 4368-4371.

Heaton RK, C. G., Talley JL, Kay GG, Curtiss G. (1993). Wisconsin Card Sorting Test manual: revised and expanded. . Odessa, Psychological Assessment Resources.

Kay, S. R., A. Fiszbein and L. A. Opler (1987). "The positive and negative syndrome scale (PANSS) for schizophrenia." Schizophrenia bulletin **13**(2): 261-276.

Kiresuk, T. J., A. Smith and J. E. Cardillo (1994). Goal attainment scaling: Applications, theory, and measurement. Hillsdale, NJ, England, Lawrence Erlbaum, Inc.

Lader, D., S. Short and J. Gershuny (2006). "The time use survey, 2005." Office for National Statistics, London.

Lejeune et al. A Meta-analysis of Cognitive Remediation for Schizophrenia: Efficacy and the Role

of Participant and Treatment Factors. Schizophrenia Bulletin vol. 47 no. 4 pp. 997–1006, 2021

Lowe, C. and P. Rabbitt (1998). "Test\re-test reliability of the CANTAB and ISPOCD neuropsychological batteries: theoretical and practical issues." Neuropsychologia **36**(9): 915-923.

McCue et al. Using the Goal Attainment Scale adapted for depression to better understand treatment outcomes in patients with major depressive disorder switching to vortioxetine: a phase 4,

single-arm, open-label, multicenter study BMC Psychiatry (2021) 21:622

Morosini, P. L., L. Magliano, L. a. Brambilla, S. Ugolini and R. Pioli (2000). "Development, reliability and acceptability of a new version of the DSM‐IV Social and Occupational Functioning Assessment Scale (SOFAS) to assess routine social funtioning." Acta Psychiatrica Scandinavica **101**(4): 323-329.

Rey, A. and P. A. Osterreith (1993). "Translations of excerpts from Andre Rey’s Psychological examination of traumatic encephalopathy and PA Osterrieth’s The complex figure copy test." Clin Neuropsychol **7**(1): 4-21.

Rannisto et al. The use of goal attainment scaling in neuropsychological rehabilitation in multiple sclerosis Disabil Rehabil, 2015; 37(21): 1984–1991

Rockwood et al. Use of goal attainment scaling in measuring clinically important change in cognitive rehabilitation patients. J Clin Epidemiol 1997; 50:581–8.

Rockwood et al., Responsiveness of goal attainment scaling in a randomized controlled trial of comprehensive geriatric assessment Journal of Clinical Epidemiology 56 (2003) 736–743

Rockwood et al. Attainment of treatment goals by people with Alzheimer’s disease receiving galantamine: a randomized controlled trial. CMAJ 2006;174(8):1099

Rosenberg, M. (1965). "Rosenberg self-esteem scale (RSE)." Acceptance and commitment therapy. Measures package **61**(52): 18.

Stolee e al. An Individualized Approach to Outcome Measurement in Geriatric Rehabilitation

Journal of Gerontology: MEDICAL SCIENCES 1999, Vol. 54A, No. 12, M641-M647

Turner-Stokes, L. (2009). "Goal attainment scaling (GAS) in rehabilitation: a practical guide." Clinical rehabilitation **23**(4): 362-370.

Wechsler, D. (1997). WASI-II, Psychological Corporation San Antonio, TX.

Wechsler, D. (1997). Wechsler Adult Intelligence Scale (3rd Ed). San Antonio, TX, Harcourt Assessment.

Wechsler, D. (2001). Wechsler Test of Adult Reading (WTAR). San Antonio, TX, The Psychological Corporation.

Wykes, T. & Reeder C. (2005). Cognitive Remediation Therapy for Schizophrenia: Theory and Practice. Routledge.

ECLIPSE (CIRCUITS study)

A randomised controlled trial of cognitive remediation therapy in patients with non-affective psychosis

Statistical Analysis Plan for End of Trial

Version 1.0

Version 1.0 started: 27/07/2020

ISRCTN: ISRCTN14678860

Trial Statistician: Dominic Stringer

Signature
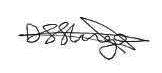
 Date: 23/09/2020

Chief Investigator: Professor Dame Til Wykes

Signature:
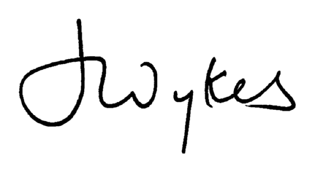
 Date: 23^rd^ September 2020

Trial Steering Committee Chair: Professor David Kingdon

Signature............
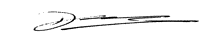
..... Date....26/08/20....................

This document details the presentation and analysis strategy for the primary paper reporting results from the ECLIPSE (CIRCuiTS study) trial. It is intended that the results reported in these papers will follow this strategy; subsequent papers of a more exploratory nature will not be bound by this strategy but will be expected to follow the broad principles laid down for the principal paper(s). These principles are not intended to curtail exploratory analysis or to prohibit sensible statistical and reporting practices but are intended to establish the strategy that will be followed as closely as possible, when analysing and reporting the trial. Reference was made to the trial protocol (ECLIPSE Study 9 Research Protocol V1.5 18 12 2018), ICH [1] guidelines on Statistical Principles (E9) and CONSORT [2] guidelines.

**12. Statistical Analysis Plan**

# QUANTITATIVE ANALYSIS PLAN

Investigators

Dr Clare Reeder

Dr Diana Rose

Dr Matteo Cella

Dr Gregory Aarons

Prof Sue Dopson

Prof Sonia Johnson

Prof Max Birchwood

Prof David Fowler

Dr Jesus Perez

Prof Andrew Pickles

Prof Kathy Greenwood

Prof Rachel Upthegrove

Dr Andrew Thompson

Dr Rosa Ritunnano

Joint Chief investigators

Prof Til Wykes

Prof Eileen Joyce

Trial manager

Dr Emese Csipke

Trial statistician

Prof Andrew Pickles

Dominic Stringer

Trial health economist

Prof Paul McCrone

## Description of the trial

ECLIPSE is a three-year trial of computerised cognitive remediation (CIRCUITS). This will be conducted in the Early Intervention Services (EIS) of NHS Mental Health Trusts with catchment areas ranging from high density urban to rural. Three different methods of providing CR will be evaluated: One-to-One, group, independent and a comparator of treatment as usual.

### Principal research objectives to be addressed

Primary objectives

To determine the best way of introducing cognitive remediation therapy (CR) for psychosis into NHS early intervention services in order to optimise individual functional outcomes.

To do this we will conduct a randomised controlled trial of 3 CR implementation methods and treatment as usual to evaluate:

1. The effectiveness of each CR method in the achievement of personal goals as measured by the total score on the Goal Attainment Scale.

Secondary objectives

1. The effectiveness of each CR method in improving individual components that might contribute to goal attainment (cognition, social function, self-esteem and symptoms)
2. The cost-effectiveness of each implementation method by combining service costs (derived from administrative data and the Client Service Receipt Inventory) and quality-adjusted life years (measured by EQ-5D)
3. To compare service use and costs at 15 weeks post-randomisation (post-therapy assessment)

Secondary objectives 3 and 4 are objectives of the economic analysis and will not be covered in this document.

This SAP relates only to the main paper resulting from the main outcome (GAS) results of study 9. This trial is part of a larger programme grant on the advice that can be given to the NHS to implement cognitive remediation. Information gleaned from the costs of treatment, cost-effectiveness as well as participant and staff satisfaction will contribute to the overall final recommendations and conclusions. How this recommendation will be formed is not part of this SAP.

### Trial design including blinding

ECLIPSE is a randomised four-arm trial of three different implementation modes of Cognitive Remediation Therapy (CR) compared to Treatment as Usual in people presenting with non-affective psychosis. However, from 19/02/2019, the Independent and Treatment arms were dropped, and the trial is continuing to randomise to the Group CR and One-to-One CR arms only.

Clinicians and research workers completing baseline and follow up assessments (including outcome assessment) will be blind to group allocation. Blinding will be maintained by ensuring patient data are stored in separate offices, locating research and therapy staff in separate offices and, where possible, third party management of appointments to avoid appointment clashes. In the case of a research worker becoming unblinded, another researcher will be sent to the site to complete the rest of the assessments and this will be recorded.

Therapists and patients will necessarily not be blind to group allocation (following randomisation).

The trial has four groups, although the Independent and TAU arms were dropped as of 19/02/2019 and the trial will randomise only to the One-to-One CR and Group CR arms from this date.

1. One-to-One CR

(a) All participants are offered 10.5 weeks of twice weekly individual therapy.

(b) Sessions (60-180 minutes duration) are in 3 parts: (1) 20-60 minutes of CR with a therapist; (2) 20-60 minutes of in vivo transfer work (i.e. putting CR strategies into real life) with a therapist; (3) 20-60 minutes of independent CR, set up by the therapist on site, or done off-site in the patient’s own time.

(c) Each patient receives up to 42 hours of CR (21 with therapist, 21 independently) and 21 hours of in vivo transfer work (with a therapist).

2.Group CR

(a) All participants attend 14 weeks of three times weekly group therapy (up to 42 hours of CR in total).

(b) Group sessions last 1 hour, with attendance for at least 20 minutes considered have completed a session.

(c) Participants will join the group as soon as possible following randomisation. Each group will have one therapist.

(d) Group sessions begin and end with group activities, relating to goal-setting and metacognition. During the rest of the session, patients work independently on CIRCuiTS tasks (at the same time) with the therapist offering help and support to individuals on an as-needed basis.

3. Independent CR

(a) All participants are offered one individual session to get started.

(b) Following this, participants are offered up to 41 independent sessions (up to 42 hours of CR in total).

(c) To support the independent sessions, the therapist offers telephone contact and/or attendance at daily drop-in sessions on an as needed basis (estimated average therapist time: 1 hour per fortnight).

4. Treatment as usual

This is defined as multi-modal treatment and will consist of different therapies as defined as necessary by the treating team.

**Figure 3 Trial design flow diagram**


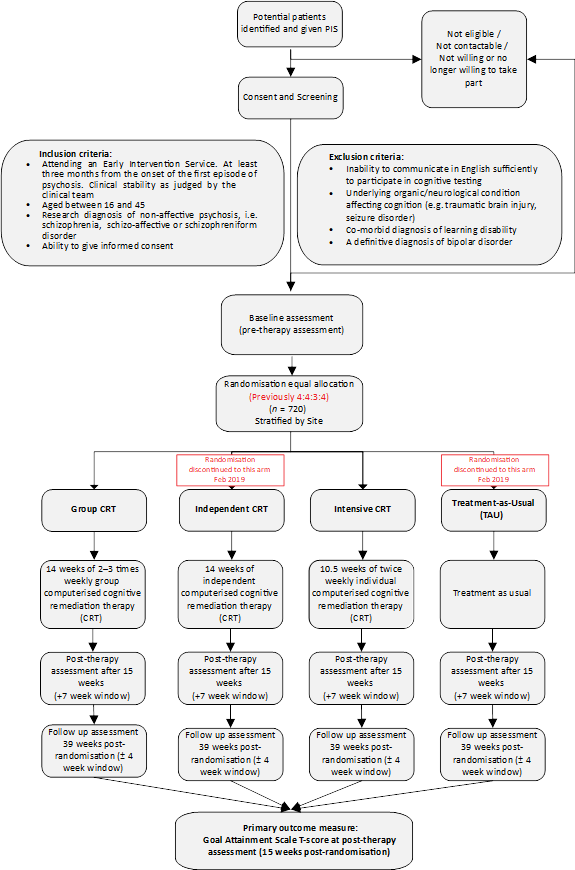


### Method of allocation of groups

Originally, consented patients were randomised in blocks of 15 stratified by research site with randomisation in proportions 4:4:3:4 (group CR/independent CR/ One-to-One CR/ treatment-as-usual). Alternative proportions to 4:4:3:4 were used for blocks of less than 15 participants.

Following difficulties in timely recruitment of complete blocks of patients, the treatment allocation process was then changed to randomisation of individual patients with equal allocation to the 4-arms, stratified by site, using a random sequence of blocks of variable size. This was further changed to be randomisation to 2-arms (otherwise with the same specification) when the independent and treatment as usual arms were dropped.

This was implemented using an independent web-based randomisation service at the UKCRC registered King's Clinical Trials Unit (CTU).  Participant allocation will be communicated only to therapists by email.

### Duration of the treatment period

The treatment period will be for 14 weeks from 0 weeks post-randomisation to 14 weeks post-randomisation. Participants in the One-to-One CR arm are offered 10.5 weeks of twice weekly individual therapy. Participants in the Group CR arm attend 14 weeks of three times weekly group therapy.

Participants who were previously randomised the Independent CR arm were offered up to 41 independent sessions over the 14 weeks. Participants who were previously randomised the Treatment as usual arm continued to receive treatment as usual over the 14 weeks.

### Frequency and duration of follow-up

Participants will complete follow up measures following therapy (15 weeks post-randomisation) and at 6 months post-therapy (39 weeks post-randomisation).

### Visit windows

The following visit windows apply to the assessments carried out in the trial:

- Pre-therapy (baseline) assessment; 12 to 0 weeks pre-randomisation.
- Post-therapy assessment; 15 weeks post randomisation +4-week window, i.e. 15- and 22-weeks post-randomisation.
- 6-month post-therapy assessment; 39 weeks post-randomisation with +/- 4-week window i.e. 35 to 44 weeks post-randomisation.

We will however utilise all available data in the analyses, regardless of when visit actually occurred. A sensitivity analysis will be carried out on the effect of using only data from assessments that complied with the visit windows (see Section 3.14)

### Data collection

#### Eligibility screening

Inclusion criteria

- Attending an Early Intervention Service or any individual within 5 years of their first episode of psychosis. At least three months from the onset of the first episode of psychosis. Clinical stability as judged by the clinical team;
- Aged between 16 and 45;
- A research diagnosis of non-affective psychosis, i.e. schizophrenia, schizo-affective or schizophreniform disorder;
- Ability to give informed consent.

Exclusion criteria

- Inability to communicate in English sufficiently to participate in cognitive testing;
- Underlying organic/neurological condition affecting cognition (e.g. traumatic brain injury, seizure disorder);
- Co-morbid diagnosis of learning disability.
- A definitive diagnosis of bipolar disorder

#### Measures

A timeline of data collected is given in the Schedule of Assessments and Measures (section B of this document). What follows is a brief overview to aid understanding of the analysis plan.

Measures collected at Baseline only

- Eligibility and Consent
- Mini International Neuropsychiatric Interview (MINI)
- Sociodemographics (ethnicity, employment, living situation, and relationship status)
- Background Trauma and Risk Information
- Duration of Untreated Psychosis (DUP) assessed using the relevant sections of the Nottingham Onset Schedule (NOS)
- The Wechsler Test of Adult Reading
- Wechsler Abbreviated Scale of Intelligence II

Primary outcome measure at 15 weeks post-randomisation (also collected at Baseline)

- The Goal Attainment Scale (GAS)

Secondary outcome measures at 15 weeks and 39 weeks post-randomisation (also collected at Baseline)

**The following measures will form part of the Primary outcome paper:**

- The Goal Attainment Scale (GAS) (at 39 weeks)
- Social and Occupational Functioning Assessment Scale (SOFAS)
- The Time Use Survey – total hours in structured activity
- CAINS total score
- Composite Cognitive score as measured using the Cambridge Neuropsychological Test Automated Battery (CANTAB). The included CANTAB measures will be:

1. Motor Screening Task: MOT

2. Reaction Time: RTI

3. Paired Associated Learning: PAL

4. Spatial Working Memory

5. One-touch Stockings of Cambridge: OTS

6. Emotion Recognition Task: ERT

7. Rapid Visual Information Processing: RVP

8. Attention Switching Task: AST

- Self-esteem as measured using the Rosenberg Self Esteem Scale

**The following measures are process outcomes (not collected in TAU arm) rather than necessarily secondary outcomes and will be compared between arms in the primary paper:**

- Bespoke satisfaction measure (service users)
- Bespoke satisfaction measure (staff)
- Engagement with therapy

**The following measures relate to the Economic analyses to be carried out by the Health economists (will not be covered by this SAP but will be in primary paper):**

- The Client Service Receipt Inventory
- The EQ-5D

**The following measures will be exploratory or potential moderators/mediators and will not be analysed in the primary outcome paper (will not be covered by this SAP)**

- Adapted History of Substance Use (alcohol and Drugs)
- Computerised Wisconsin Card Sorting Task (WCST)
- Digit Span task
- Rey Auditory Verbal Learning Test
- Rey Osterrieth Complex Figure
- IoPPN Narrative Metacognition Task
- Metacognition Assessment Scale for the Rey Osterrieth Complex Figure
- Self-appraisal Scale (devised for the purposes of this study).
- The IoPPN Narrative Metacognition Task and the Self-Appraisal scale will also be done after the Spatial Working Memory (SWM) subtest of the CANTAB
- Measure of Insight into Cognition - Self Report (MIC-SR)
- Cognitive reserve (measured from WTAR and WASI 11)
- Duration of untreated psychosis (NOS)
- Premorbid social adjustment (PAS) (removed in most recent visit schedule so not collected on all participants)
- Working alliance inventory
- Brief Core Schema Scale (removed in most recent visit schedule so not collected on all participants)
- Calgary Depression Scale total score (removed in most recent visit schedule so not collected on all participants)
- Frontal Systems Behavior Scale

**Adverse events**

- Reported adverse events

**Additional post-randomisation (follow-up) measures (to be described only)**

- Withdrawal from follow up
- Psychiatric medication being taken at time of assessment
- PANSS total score

### Sample size estimation (including clinical significance)

Sample size

A total of 720 first episode psychosis patients will be recruited.

Power

We have the capacity to recruit 900 patients (from 1500 attending 10 services for 3 years) and have allowed for a 20% drop-out pre-randomisation.

Using a design with parallel arms of equal size with 180 patients per arm provides approximately 80% power for a simple group ES difference of 0.3 with alpha=0.05. This increases to 91% for outcomes that correlate 0.5 with baseline (both calculated using sampsi in Stata).

This power calculation was based on comparisons between each of the active arms and TAU. While testing against TAU would be expected to require only a modest sample size, the effect size differences among the active arms are likely to be less substantial.

This power calculation was not controlled for multiple comparisons. Friedlin et al [3] suggest no great advantage in accounting for multiple testing in a multi-arm trial, and also that the advantages of a larger TAU arm are more slight than commonly assumed. Interaction among patients in group delivery is very slight so no allowance for clustering was thought necessary.

The power calculation is based on arms of equal size; the difference in power as a result of the initial unequal allocation is likely to be small as the use of modestly unequal randomisation ratios only very slightly reduces the power of a study ([4]).

### Interim analysis

Planned interim analysis at start of trial

A planned interim analysis was to be undertaken by the health economist after recruitment of the first 195 patients (using the post-therapy data at 15 weeks post-randomisation). The economist was not to be blind to trial arm because the costs were to be specific to the intervention provided. That analysis might have resulted in one of the trial arms being closed, with an immediate impact on the randomisation of the next patients.

The decision was to depend on the cost of therapy and the outcomes achieved from it. The relevant outcomes were to be time use, goal attainment, and service user satisfaction. The costs were to be confined to those of the direct therapy inputs and not the cost of other services derived from the Client Service Receipt Inventory. The direct therapy costs were to be calculated from data on the number and length of sessions, number of attendees (for group therapy), and unit costs based on staff grade and overheads. Missing outcome data were to be handled as described for the main analysis. Cost-effectiveness planes were to be generated by plotting 1000 incremental cost-outcome combinations derived using bootstrapping from the sample and comparing each pair of therapies. This would tell us the probability that one therapy had (i) lower costs and better outcomes, (ii) lower costs and worse outcomes, (iii) higher costs and better outcomes, and (iv) higher costs and better outcomes than a comparator. Repeated for each outcome measure, a decision to drop an arm would have been taken if the probability of a good result fell below 25% for each.

Actual interim analysis and contingent recruitment decisions

In the light of recruitment falling short of the target for the interim analysis, and for the need to concentrate remaining recruitment time on fewer arms, an ad-hoc interim analysis was undertaken. Undertaken in November 2018 on the 100 participants with endpoint data at that time, evidence for each trial arm was reviewed by the DMC against the following criteria

1. Treatment engagement – an arm that has more than 50% of individuals receiving therapy for less than 5 hours.
2. Cost-effectiveness –more than **£500 increase in costs in an arm** per one point increase in cognition (visual and verbal memory) or for one hour of structured activity
3. Participant satisfaction *–*25% of participants disagree with the statement, “Overall I was satisfied with the CIRCuiTS therapy”

Treatment engagement and satisfaction had a high clinical value when considering dropping an arm. Cost-effectiveness was be considered but a key question for the NHS is how to differentiate between the two more costly arms (group treatment and One-to-One treatment) so the costs for improvement would have to be much greater between these two comparison arms to be confident in dropping one of them. Cost-effectiveness would therefore have less clinical value in making a decision than the other two criteria. If the two high value criteria differ in their conclusions (i.e. suggest different arms to drop) then a clinical judgement would favour making a decision on the basis of user led direct information – satisfaction.

The DMEC recommendations were as follows:

1. Drop Independent CR arm
2. Provide power calculations for dropping the Independent CR arm and retaining TAU as well as dropping both and seek opinion from NIHR regarding dropping or retaining TAU on this basis. If NIHR require a clear opinion from the DMEC then the balance of opinion was in favour of retaining TAU.

Programme Steering Committee and Patient Advisory Board involvement

The draft report was sent to the Patient Advisory Board and the Eclipse Programme Steering Committee who supported the proposal to **drop both Independent arm and TAU** which was the preferred option of the study team and was subsequently approved by NIHR.

Revised Power Calculation

Potential sample sizes were calculated following alternative decisions using an overall expected sample size of 438 participants. For the scenario with retaining the Group and One-to-One arms, this gave an expected total of 158 and 141 participants in these arms respectively at the end of the trial. For the contrast of Group vs One-to-One, assuming 80% with endpoint and follow-up data, with plausible correlation structure (correlation between follow up measures=0.5 and correlation between baseline and follow up =0.2), but making no allowance for clustering and retaining the effect size of 0.3 for a comparison of active-arms gave nominal 79% power (two-tailed alpha=.05).

### Impact of the ad-hoc interim analysis on the proposed main analysis

The primary outcome for the main analysis was to be the Goal-Attainment Scale (GAS). While it would be usual to account for the impact of an interim analysis on trial analysis undertaken at the end of recruitment, the relationship between the ad-hoc interim analysis and final analysis is not one that could be easily formalised. We therefore propose undertaking a naïve analysis of the trial, that assumes that decisions made on arm-specific recruitment stoppage were made independent of GAS scores on the available sample. Additionally, two sensitivity simulations would then be undertaken, based on the baseline data, and the observed missing data pattern.

## Data analysis plan – Data description

### Recruitment and representativeness of recruited patients

A CONSORT flow chart will be constructed [2] – see Figure 2. This will include the number of eligible patients, number of patients agreeing to enter the trial, number of patients refusing, numbers randomised to each treatment arm: the number of patients who received at least 1 therapy session, the number continuing through the trial, the number withdrawing, the number lost to follow-up and the numbers excluded/analysed.

Figure 2. Template CONSORT diagram for ECLIPSE trial


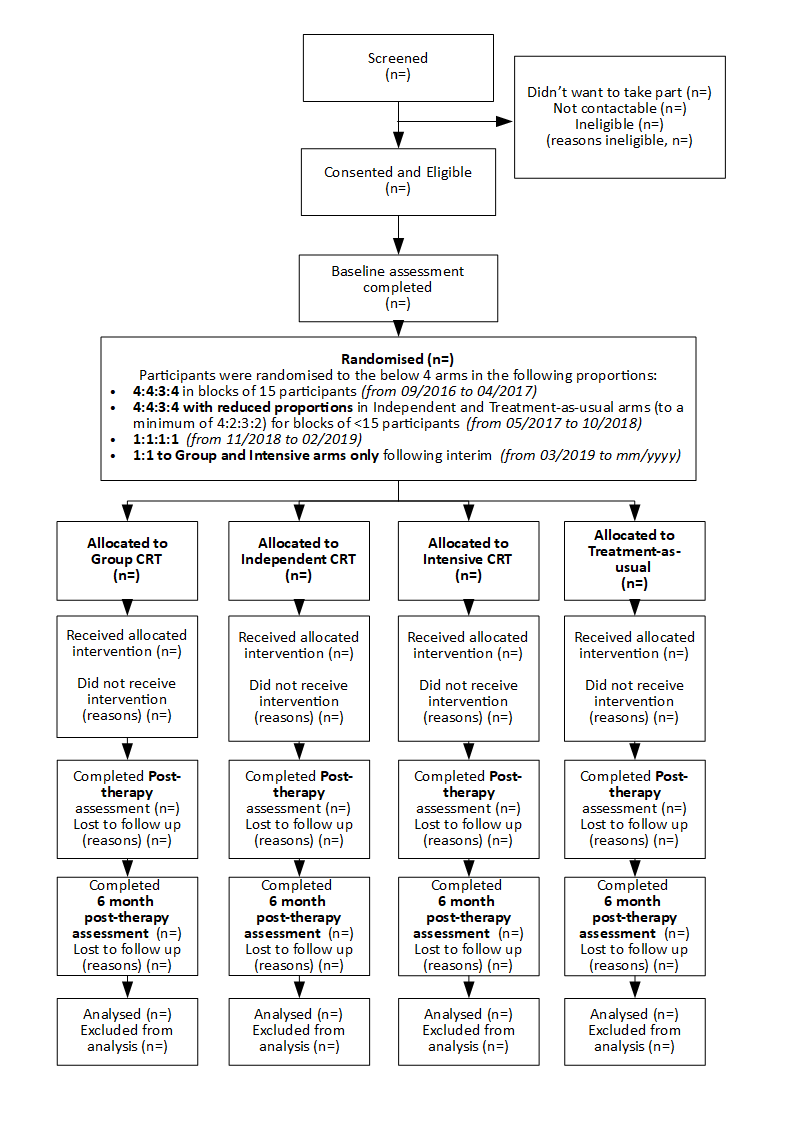


### Baseline comparability of randomised groups

Baseline descriptions of participants by treatment arm and overall: means and standard deviation or numbers and proportions as appropriate. No significance testing will be used to test baseline differences between the randomised treatment groups.[5]

All baseline variables listed under measures in section 1.7 will be reported overall and by trial arm. Baseline values of primary and secondary outcomes will also be summarised overall and by trial arm.

### Adherence to allocated treatment and treatment fidelity

Compliance will be a continuous measure of number of valid sessions the participant attended and will be described by treatment arm and in terms of baseline variables. Compliance will be ascertained from the therapy audit forms.

### Loss to follow-up and other missing data

It is the aim of the trial to minimise withdrawal of participants from treatment and follow-up. Completion of a withdrawal from trial is regarded as withdrawal from data collection/follow-up in this analysis.

Withdrawal from trial will be reported by intervention group. The proportions of participants missing each variable will be summarised in each arm and at each time point. The numbers, proportions and reasons for withdrawal from trial will be summarised by treatment arm. The distribution of times between randomisation and withdrawal from follow-up will be summarised using a histogram.

The baseline characteristics of those missing follow-up will be compared to those with complete follow-up. The relationship between baseline characteristics and missing data will be investigated graphically.

### Adverse event reporting

Adverse events (AE) and serious adverse events (SAE) will be summarised by treatment arm.

### Assessment of outcome measures (unblinding)

Outcome assessors (research workers) and the senior trial statistician are being kept blind to treatment allocation.

### Descriptive statistics for outcome measures

Each of the outcome measures will be described by treatment group. Means and standard deviations or medians and interquartile ranges will be used for continuous variables; Q-Q plots will be used to assess whether the distribution of a variable is normal. Frequencies and proportions will be used to describe categorical variables.

## Data analysis plan – Inferential analysis

### Main analysis of treatment differences

All analyses will use the intention-to-treat population unless otherwise specified (i.e. all randomised participants included according to allocated randomised trial arm irrespective of treatment received). The formal statistical analyses will use the following sequence of contrasts for the primary and all secondary outcomes:

- 1. Group versus One-to-One
  2. Independent versus TAU
  3. Group+One-to-One vs TAU

Estimates of differences between groups and associated 95% confidence intervals will be reported.

The significance level will be 5% (two-sided) for all outcomes. No adjustment will be made for the multiple contrasts. Sensitivity analyses will be used to assess the robustness of conclusions to non-ignorable missing outcome data.

The senior trial statistician will remain blind until the main analyses are completed. Any analyses that cannot be performed blind will be done at the end of the final analysis in order to preserve blinding for as long as possible.

### Analysis of primary outcomes

The analysis population will include all patients and intended to recover ITT estimates of effects. Patients with missing baseline measurements will be included using the mean imputation and dummy variable approach (White and Horton [7]).

The primary outcome is Goal Attainment Scale (GAS) T-score at 15 weeks post-randomisation (post-therapy assessment). The Goal Attainment Scale T-score is a standardized measure which will be calculated from the goals set in the GAS weighted by importance and difficulty using a formula as specified in the GAS Practical Guide [6] and as detailed in Appendix 1. If goals are set in an unbiased fashion, we would expect this measure to be normally distributed [6].

A linear mixed model will be used to estimate difference in mean GAS T-score at 15 weeks between arms. Linear mixed modelling utilises all available information (including the 40-week follow-up assessment), leading to more precise estimates of the treatment effect. This technique will allow the simultaneous modelling of the repeated outcome time points.

In such models the outcome variable measured at the post treatment time points (here post-therapy and follow up) features as the dependent variable, with treatment arm, time (post-therapy or follow up), a time by treatment arm interaction, baseline GAS T-score and the randomization stratifier (Site) included as independent variables and a random patient-specific intercept. We will additionally include period as a covariate as described below in section 3.5.

All arms will be modelled simultaneously, and post-estimation commands used to obtain separate estimates for each of the specified contrasts at the post-therapy and follow up timepoints.

### Analysis of secondary outcomes

Treatment effects on all secondary outcomes that were measured repeatedly over the follow-up period will lend themselves to the same analysis as described above for the primary outcome. These secondary outcomes will be assessed using similar modelling techniques, employing generalisations to non-normal data where necessary or transformation of the outcome variable. Where secondary outcomes are only measured once over the follow up period, generalised linear models will be used.

### Time points

The primary and secondary analyses use all available data from post-therapy (15 weeks post-randomisation), post therapy (39 weeks post-randomisation) and baseline assessments. Deviations of measurements from planned time points will be summarised by treatment group.

### Stratification and clustering

Randomisation is stratified by site; therefore, this variable will be included as a factor in the modelling process as detailed in 3.2. There is limited interaction expected between participants in the group therapy arm and so we will not account for any clustering effects within the group arm; however, a sensitivity analysis may be conducted to test this assumption if data allows.

We will additionally adjust for period as a factor in all of the models. Period will be a binary variable of whether participant was randomised before or when the Independent and TAU arms were dropped (following the interim analysis). This is primarily as randomised participants in the two periods may not be exchangeable under their respective null hypotheses, as well as for reasons of potential bias as outlined further in Section 3.12.

### Missing items in scales and subscales

The number (%) with complete data will be reported. Where present, missing value guidance provided for scales will be used. Where this is not possible, scales will be pro-rated for an individual if 20% or fewer items are missing. For example, in a scale with 10 items, prorating will be applied to individuals with 1 or 2 items missing. The average value for the 8 or 9 complete items will be calculated for that individual and used to replace the missing values. The scale score will be calculated based on the complete values and these replacements.

### Missing baseline data

Missing baseline data should not be an issue for the primary analysis. Some extensions to this analysis may use other baseline variables; if these contain missing data, the number with complete data will be reported and they will be imputed using a method suitable to the variable as per the recommendations of White and Thompson [7] .

### Missing outcome data

Analyses will be undertaken assuming outcomes are missing-at-random and using all available data. This allows drop-out to be related to treatment group, stratification factors, period and baseline severity. Sensitivity analyses will be carried out to assess (1) the association of drop-out with baseline demographic variables and their inclusion as additional covariates (2) last observation carried forward as an alternative assumption.

### Method for handling multiple comparisons

No formal adjustment of p-values for multiple testing or as a consequence of multiple comparisons will be made, However, care will be given to the interpretation of inference for the numerous secondary outcomes and with respect to multiple contrasts. The absence of any correction will be reported.

### Method for handling non-compliance (per protocol/CACE analyses)

In addition to the primary intention-to-treat analysis the effect of actually receiving treatment will be estimated. Compliance will be a continuous measure of the number of valid therapy sessions attended. Local average or complier average treatment effects per hour of active CR compared to TAU will be estimated. On an assumption of a common effect per hour of active CR, evidence for differences in treatment mode not explained by treatment hours of active CR will be presented.

### Model assumption checks

The models assume normally distributed outcomes; residual plots will be checked for normality and outliers and if substantial departures occur, transformations will be applied.

### Sensitivity to interim analysis

Sensitivity analyses will be carried out to assess the impact of dropping the Independent and TAU arms consequent to the interim analysis. There are at least 3 mechanisms by which this may affect these results:

- The interim analysis was partially based on the primary outcome. This could theoretically lead to bias in the treatment estimate as the primary outcome at the interim for the dropped arms may have been lower than the continuing arms by chance.
- Participants interested in participating and consequently recruited to the 2 arm version of the trial may differ in characteristics from than those that were interested in participating and were recruited to the 4 arm version (e.g. the latter included a Treatment as usual arm which may have dissuaded participants from participating).
- There may be a “time of recruitment” effect as comparisons between continuing arms and dropped arms will include non-contemporaneous participants.

We will report simulation results under two scenarios:

1. Simulate all 4 arms under the global null hypothesis to the interim point
   1. Select those scenarios where the 2 arms selected are the best two according to the primary outcome. Whilst this does not reflect the decision criteria that was used at the interim analysis, this should provide an upper bound on the degree of bias. Progress simulation to end of recruitment. Estimate effects as per SAP
   2. Continue simulation to end of recruitment for all scenarios. Estimate effects as per SAP
   3. Compare distributions of the average treatment effect estimates under a and b
2. As above but simulated under parameters of the naïve analysis. This is equivalent to simulating under a “global alternative hypothesis”.

### Sensitivity to circumstances surrounding COVID-19 pandemic

We believe it is unlikely that the effects of the pandemic/lockdown (which has led to changes in the way outcomes were recorded) will introduce bias for the comparison of contemporaneous arms, as any effects are unlikely to differ by arm.

We expect the pandemic/lockdown will affect comparisons between the arms that were dropped at interim (TAU and Independent CR) and the ongoing arms (One-to-One and Group CR). This is because the ongoing arms will have participants whose follow up measures were affected by the pandemic and the dropped arms won’t. However, as described in Section 3.12, there are already other potential sources of bias in these comparisons. We are already attempting to account for these by including period (before or after interim) as a covariate in the analysis and assessing further impact by carrying out simulations. Any potential for bias in these comparisons due to the pandemic will be assessed in the same way.

### Sensitivity to visit windows

As per Section 1.6, visit windows are defined in the protocol for the post-therapy and 6-month post-therapy follow up assessments anchored to date of randomisation. However, where visits have occurred outside these windows, data has still been recorded and will be used for the primary analysis. A sensitivity analysis will be carried out (on the primary outcome only) using only data from visits that occurred within the visit windows.

### Planned subgroup analyses

No subgroup analyses are planned for the primary paper. The study is not powered to investigate interaction effects.

### Exploratory analyses

This analysis plan does not cover exploratory analyses. Exploratory mediator and moderator analyses may be performed after the primary trial data analysis.

## Software

Data management: An online data collection system for clinical trials (MACRO; InferMed Ltd) will be used. This is hosted on a dedicated server at KCL and managed by the KCTU. The KCTU will extract data periodically as needed and provide these in comma separated (.csv) format.

Statistical analysis: Stata 15 [8] will be used for data description and inferential analyses. R [9] may additionally be used for data description and production of graphs, tables and reports.

| SCHEDULE OF ASSESSMENTS AND MEASURES |
| --- |

| **#** | **Form** | **Screening assessment** | **Pre- therapy assessment** | **Post- therapy assessment** | **6-month Follow-up** | **Type** | **Administered to** | **Administration time** |
| --- | --- | --- | --- | --- | --- | --- | --- | --- |
| **MAIN DATABASE** | | | | | | | | |
| 1 | **Eligibility form** | x |  |  |  | Examination of case notes | Completed by RW | n/a  (completed by RW) |
| 2 | **Mini International Neuropsychiatric Interview (MINI)** | x |  |  | x | Diagnostic checklist | Participant/Notes | **5 min**  (3 sections only) |
| 3 | **Registration and Demographics Form** | x |  |  |  | Questionnaire | Participant | **2min** |
| 4 | **Nottingham Onset Schedule (NOS)** | x |  |  |  | Semi-structured interview | Participant *(&case notes)* | **5min** |
| 5 | **Social and Occupational Functioning Assessment Scale (SOFAS)** |  | x | x | x | Rating scale | Completed by RW | n/a  (completed by RW) |
| 6 | **Adapted Substance Misuse Questionnaire** |  | x | x | x | Questionnaire | Participant | **5min** |
| 7 | **Positive and Negative Symptom Scale (PANSS)** |  | x | x | x | Semi-structured interview | Participant | **20-30min** |
| 8 | **Clinical Assessment Interview for Negative Symptoms (CAINS)** |  | x | x | x | Semi-structured interview | Participant | **20min** |
| 9 | **The Time Use Survey** |  | x | x | x | Questionnaire | Participant | **5-10min** |
| 10 | **The Client Service Receipt Inventory** |  | x | x | x | Questionnaire | Participant | **10-15min** |
| 11 | **EQ-5D-5L** |  | x | x | x | Questionnaire | Self-report | **2min** |
| 12 | **Rosenberg Self Esteem Scale** |  | x | x | x | Questionnaire | Self-report | **2 min** |
| 13 | **CANTAB - Motor Screening Task: MOT** |  | x | x | x | Computerised test | Participant | **7min** |
| 14 | **CANTAB - Reaction Time: RTI** |  | x | x | x | Computerised test | Participant | **8min** |
| 15 | **CANTAB - Paired Associates Learning (PAL)** |  | x | x | x | Computerised test | Participant | **10min** |
| 16 | **CANTAB – Spatial Working Memory** |  | x | x | x | Computerised test | Participant | **5min** |
| 17 | **CANTAB - One-touch Stockings of Cambridge: OTS** |  | x | x | x | Computerised test | Participant | **6min** |
| 18 | **CANTAB - Emotion Recognition Test (ERT)** |  | x | x | x | Computerised test | Participant | **10min** |
| 19 | **CANTAB - Rapid Visual Information Processing: RVP** |  | x | x | x | Computerised test | Participant | **10min** |
| 20 | **CANTAB - Attention Switching Task: AST** |  | x | x | x | Computerised test | Participant | **10min** |
| 21 | **Computerised Wisconsin Card Sorting Task (WCST)** |  | x | x | x | Computerised test | Participant | **10min** |
| 22 | **Digit Span task** |  | x | x | x | Cognitive test | Participant | **5min** |
| 23 | **Rey Auditory Verbal Learning Test** |  | x | x | x | Cognitive test | Participant | **10-15min**  (30min delay) |
| 24 | **Rey Osterrieth Complex Figure** |  | x | x | x | Cognitive test | Participant | **10-15min**  (copy/trial, 3min later, 30 min later) |
| 25 | **The Wechsler Test of Adult Reading** |  | x |  |  | Cognitive test | Participant | **10min** |
| 26 | **Wechsler Abbreviated Scale of Intelligence II** |  | x |  |  | Cognitive test | Participant | **20-30min** |
| 27 | **Frontal Systems Behavior Scale** |  | x | x | x | Questionnaire | Completed by a relative/carer | n/a  (completed by relative/carer) |
| 28 | **IoPPN Narrative Metacognition Task** |  | 2x  Completed twice  (after Rey & SWM) | 2x  Completed twice  (after Rey & SWM) | x | Semi-structured interview | Participant | **2min** |
| 29 | **Metacognition Assessment Scale for the Rey Osterrieth Complex Figure** |  | x | x | x | Questionnaire | Self-report | **1min** |
| 30 | **Self-appraisal Scale** |  | 2x  Completed twice  (after Rey & SWM) | 2x  Completed twice  (after Rey & SWM) | x | Questionnaire | Self-report | **1min** |
| 31 | **Measure of Insight into Cognition - Self Report (MIC-SR)** |  | x | x | x | Questionnaire | Self-report | **1min** |
| 32 | **Goal Attainment Scale** |  | x  (goals set) | x  (rated) | x  (rated) | Rating scale | Participant | **10-15min (?)** |
| 33 | **Adverse events** | When required | | | | n/a | Completed by RW | n/a |
| 34 | **Withdrawal form** | When required | | | | n/a | Completed by RW | n/a |
| **THERAPY DATABASE and Other measures** | | | | | | | |  |
| 35 | **Therapy audit** | Ongoing | | |  | n/a | Completed by the therapist | n/a |
| Other 36 | **Satisfaction measure** |  |  | x |  | Questionnaire | Self-report/Online survey | **10min** |
| Other 37 | **Working alliance inventory** |  |  | x |  | Questionnaire | Self-report/Online survey | **10min** |

# REFERENCE LIST

Cella, Matteo, Clementine Edwards, Sarah Swan, Kay Elliot, Clare Reeder, and Til Wykes. 2019. 'Exploring the effects of cognitive remediation on metacognition in people with schizophrenia', *Journal of Experimental Psychopathology*, 10: 2043808719826846.

de Beurs, Edwin, Alfred Lange, Roland WB Blonk, Peter Koele, Anton JLM van Balkom, and Richard Van Dyck. 1993. 'Goal attainment scaling: an idiosyncratic method to assess treatment effectiveness in agoraphobia', *Journal of Psychopathology and Behavioral Assessment*, 15: 357-73.

Drake, Richard James, CJ Day, R Picucci, J Warburton, W Larkin, Nusrat Husain, C Reeder, T Wykes, and M Marshall. 2014. 'A naturalistic, randomized, controlled trial combining cognitive remediation with cognitive–behavioural therapy after first-episode non-affective psychosis', *Psychological Medicine*, 44: 1889-99.

Hiekkala-Tiusanen, Laura, Minna Halunen, Tuukka Mehtälä, and Tuula Kieseppä. 2019. 'Psykososiaaliset menetelmät skitsofrenian hoidossa ja kuntoutuksessa-sivuosasta tähtinäyttelijäksi?', *Duodecim*.

Hyde, Bronwyn, Matthew Thomas, Judith Gullifer, and Frances Dark. 2020. 'Trial implementation of CIRCuiTS cognitive remediation therapy for people with schizophrenia in rural Australia: Therapists experiences', *Journal of Psychosocial Rehabilitation and Mental Health*, 7: 5-14.

Palumbo, Davide, Armida Mucci, Giulia Maria Giordano, Giuseppe Piegari, Carmen Aiello, Daria Pietrafesa, Nicola Annarumma, Marcello Chieffi, Matteo Cella, and Silvana Galderisi. 2019. 'The efficacy, feasibility and acceptability of a remotely accessible use of CIRCuiTS, a computerized cognitive remediation therapy program for schizophrenia: A pilot study', *Neuropsychiatric Disease and Treatment*, 15: 3103.

Sheehan, David V, Yves Lecrubier, K Harnett Sheehan, Patricia Amorim, Juris Janavs, Emmanuelle Weiller, Thierry Hergueta, Roxy Baker, and Geoffrey C Dunbar. 1998. 'The Mini-International Neuropsychiatric Interview (MINI): the development and validation of a structured diagnostic psychiatric interview for DSM-IV and ICD-10', *Journal of clinical psychiatry*, 59: 22-33.

Shefler, Gaby, Laura Canetti, and Hadas Wiseman. 2001. 'Psychometric properties of goal‐attainment scaling in the assessment of mann's time‐limited psychotherapy', *Journal of Clinical Psychology*, 57: 971-79.

Strawbridge, Rebecca, Dimosthenis Tsapekos, John Hodsoll, Tim Mantingh, Nefize Yalin, Paul McCrone, Janet Boadu, Karine Macritchie, Matteo Cella, and Clare Reeder. 2021. 'Cognitive remediation therapy for patients with bipolar disorder: A randomised proof‐of‐concept trial', *Bipolar Disorders*, 23: 196-208.

Tabak, Naomi T, Peter C Link, Jason Holden, and Eric Granholm. 2015. 'Goal attainment scaling: tracking goal achievement in consumers with serious mental illness', *American Journal of Psychiatric Rehabilitation*, 18: 173-86.

Thomas, Matt, and Kim Rusten. 2019. 'Trial implementation of CIRCuiTS cognitive remediation therapy for people with schizophrenia in Orange, New South Wales', *Australian Journal of Rural Health*, 27: 463-68.

Van Duin, Daniëlle, Lars De Winter, Hans Kroon, Wim Veling, and Jaap Van Weeghel. 2021. 'Effects of IPS plus cognitive remediation in early psychosis: 18-month functioning outcomes of a randomized controlled trial', *Schizophrenia Research*, 236: 115-22.

EMEA, “ICH Topic E 9 Statistical Principles for Clinical Trials.” 1998.

K. F. Schulz, D. G. Altman, and D. Moher, “CONSORT 2010 Statement: updated guidelines for reporting parallel group randomised trials,” *BMJ*, vol. 340, p. c332, Mar. 2010.

B. Freidlin, E. L. Korn, R. Gray, and A. Martin, “Multi-Arm Clinical Trials of New Agents: Some Design Considerations,” *Clin. Cancer Res.*, vol. 14, no. 14, pp. 4368–4371, Jul. 2008.

S. J. Pocock, *Clinical trials: a practical approach*. Chichester: John Wiley and Sons, 1995.

S. F. Assmann, S. J. Pocock, L. E. Enos, and L. E. Kasten, “Subgroup analysis and other (mis)uses of baseline data in clinical trials,” *Lancet Lond. Engl.*, vol. 355, no. 9209, pp. 1064–1069, Mar. 2000.

L. Turner-Stokes, “Goal attainment scaling (GAS) in rehabilitation: a practical guide,” *Clin. Rehabil.*, vol. 23, no. 4, pp. 362–370, Apr. 2009.

I. R. White and S. G. Thompson, “Adjusting for partially missing baseline measurements in randomized trials,” *Stat. Med.*, vol. 24, no. 7, pp. 993–1007, Apr. 2005.

StataCorp, *Stata Statistical Software: Release 15.* College Station, TX: StataCorp LLC, 2017.

R Development Core Team, *R: A language and environment for statistical computing*. R Foundation for Statistical Computing, Vienna, Austria, 2008.

T. J. Kiresuk and R. E. Sherman, “Goal attainment scaling: A general method for evaluating comprehensive community mental health programs,” *Community Ment. Health J.*, vol. 4, no. 6, pp. 443–453, Dec. 1968.

# Appendix 1 – GAS Scoring for primary outcome

We will calculate the GAS weighted T-score by applying the following formula (as specified in the GAS Practical Guide [6]):

$$\frac{10\sum{(w}_{i}x_{i})}{{[\left( 1-\rho\right)\sum{w_{i}}^{2}+ \rho\left( {\sum{(w}_{i})}^{2} \right)]}^{\frac{1}{2}}}$$

Where:

$w_{i}$ = the weight assigned to the *i*th goal

$x_{i}$ = the numerical value achieved (between -2 and +2)

$\rho$ = the expected correlation of the goal scales, we will use 0.3 as recommended by Kirusek and Sherman [10] as this is most common approximation.

**Data Sharing Statement**

Wykes. Etc…. paper title and publication date

**Data**

***Data available***: Yes

***Data types***: Only data which does not allow participant identification

***How to access data***: Data requests must be accompanied by a prespecified analysis plan that should be publicly posted prior to receipt of any data. Please send requests for data to til.wykes@kcl.ac.uk

***When available***: With publication

**Supporting Documents**

***Document types***: N/A

***Additional Information***: study design and data analysis plan is published here: Open Science ***Framework pre-registration link***: OPEN SCIENCE LINK

***How to access documents***: Open Science Framework preregistration link:

***When available***:

**Additional Information**

***Who can access the data***: Researchers whose proposed use of the data has been approved by Eileen Joyce, Til Wykes and Dominic Stringer

***Types of analyses***: Analysis of negative emotion ratings and fMRI BOLD signal data

***Mechanisms of data availability***: researchers whose proposed use of the data has been approved
